# Long-term unsupervised recalibration of cursor-based intracortical brain–computer interfaces using a hidden Markov model

**DOI:** 10.1038/s41551-025-01536-z

**Published:** 2025-12-08

**Authors:** Guy H. Wilson, Elias A. Stein, Foram Kamdar, Donald T. Avansino, Tsam Kiu Pun, Ronnie Gross, Tommy Hosman, Tyler Singer-Clark, Anastasia Kapitonava, Leigh R. Hochberg, John D. Simeral, Krishna V. Shenoy, Shaul Druckmann, Jaimie M. Henderson, Francis R. Willett

**Affiliations:** 1 Neurosciences Graduate Program, Stanford University, Stanford, CA USA; 2 Department of Electrical Engineering, Stanford University, Stanford, CA USA; 3 Department of Neurosurgery, Stanford University, Stanford, CA USA; 4 Howard Hughes Medical Institute, Stanford University, Stanford, CA USA; 5 School of Engineering and Carney Institute for Brain Science, Brown University, Providence, RI USA; 6 VA RR&D Center for Neurorestoration and Neurotechnology, Rehabilitation Research and Development Service, Providence VA Medical Center, Providence, RI USA; 7 Department of Neurology, Harvard Medical School, Boston, MA USA; 8 Center for Neurotechnology and Neurorecovery, Department of Neurology, Massachusetts General Hospital, Boston, MA USA; 9 Wu Tsai Neurosciences Institute and Bio-X Institute, Stanford University, Stanford, CA USA; 10 Department of Neurobiology, Stanford University, Stanford, CA USA; 11 Department of Bioengineering, Stanford University, Stanford, CA USA

**Keywords:** Brain-machine interface, Computational neuroscience

## Abstract

Intracortical brain–computer interfaces (iBCIs) require frequent recalibration to maintain robust performance due to changes in neural activity that accumulate over time, which result in periods when users cannot use their device. Here we introduce a hidden Markov model to infer which targets users are moving towards during iBCI use and we retrain the system using these inferred targets, enabling unsupervised adaptation to changing neural activity. Our approach outperforms distribution alignment methods in large-scale, closed-loop simulations over two months, as well as in a closed loop with a human iBCI user over one month. Leveraging an offline dataset spanning five years of iBCI recordings, we show how target inference recalibration methods appear capable of long-term unsupervised recalibration, whereas recently proposed data-distribution-matching approaches appear to accumulate compounding errors over time. We show offline that our approach performs well on freeform datasets of a person using a home computer with an iBCI. Our results demonstrate the use of task structure to bootstrap a noisy decoder into a highly performant one, thereby overcoming one of the major barriers to clinically translating BCIs.

## Abstract (web summary)

A hidden Markov model that uses probabilistic retrospective inference allows for up to one month of unsupervised recalibration in an online cursor-based intracortical brain–machine interface.

## Main

Brain–computer interfaces (BCIs) can enable people with paralysis to interact with computers by directly translating neural activity into command signals. Non-invasive methods such as magnetoencephalography, electroencephalography and functional magnetic resonance imaging allow minimal-risk access to brain signals but are limited by low spatial or temporal resolution^1,2^. In contrast, intracortical BCIs (iBCIs) benefit from unparalleled spatial- and temporal-resolution recordings of local neuronal ensembles, resulting in some of the highest-performance communication systems to date^3,4^.

Despite these advances, iBCIs often require daily recalibration to maintain high performance in the face of signal nonstationarities^3,5,6^. Neural features exhibit drift over time, reflecting (among other causes) array movements^7^, device degradation^8^, physiological changes in single neurons^9–11^ and the influence of varying behaviour^12,13^. This drift operates on multiple timescales. Single- and multi-unit baseline firing rates can change even after a few minutes^5,9^, whereas the selectivity of their responses often varies on the order of days^12^ (but see ref. ^14^). As changes compound, decoders fit to a particular time period progressively worsen, resulting in a need for repeated recalibration. Recent efforts at building intrinsically robust decoders^15,16^ have shown promise for counteracting these changes, but nevertheless only delay the time needed until recalibration is needed. Standard recalibration procedures require ground-truth target labels for supervised training; consequently, an iBCI user has to carry out a predefined sequence of training examples and cannot engage in personal use of their device during these times.

An alternative approach that has been explored is the use of electrocorticography (ECoG) signals, which involves recording electrical activity from the surface of the brain with coarser electrode grids^2^. Owing to the larger electrode sizing, ECoG signals tend to have a robustness advantage over intracortical recordings^17–19^ and several studies have demonstrated the potential of ECoG for longer-term use in BCIs. For example, ECoG signals have enabled a patient with brainstem stroke to control a cursor on a computer screen over roughly one month^17^. However, ECoG signals do not have the same spatial resolution as intracortical recordings, with each electrode recording the summed activity of many thousands of neurons^20^. They therefore result in lower-bandwidth cursor BCI control^3,21,22^.

To maintain the benefits of intracortical recordings with a high signal-to-noise ratio (SNR) while mitigating signal instability, many groups have examined unsupervised recalibration methods for decoders^5,6,23–28^. These are algorithms that recalibrate using the neural features only and do not require ground-truth knowledge of where the targets are located, which one was cued or even the user’s intentions. Recent work has focused on domain mapping strategies^6,25,27–29^, where a function *f* is sought such that the distribution of neural features from an unlabelled test set *P*_test_ matches that from a training period *P*_train_ when mapped through *f* (that is, $f\left({P}_{\mathrm{test}}\right) \sim {P}_{\mathrm{train}}$). In this case, the assumption is that with proper choice of *f* the subsequent mapping from features to targets is preserved. For noisy high-dimensional neural recordings, *f* usually operates in a low-dimensional subspace that encodes the majority of task-related modulation^6,11,30,31^. In particular, Factor Analysis (FA) Stabilization uses an FA model to identify task subspaces on two different days. A Procrustes realignment within these spaces is then used to realign new data so that old decoders, trained on the old subspace, can work. More recently, a method known as the adversarial domain adaptation network (ADAN) improved on FA stabilization by leveraging deep learning for nonlinear alignment^25^.

In this work, we propose an alternative approach. Instead of stabilizing the feature distribution *P(x)* through a domain mapping strategy, we make use of *P(y)*, which is the previous knowledge of task structure, by leveraging a probabilistic model to infer user intentions during two-dimensional (2D) cursor control. This is analogous to how language models denoise earlier stages of automatic speech recognition systems, making use of linguistic structure to guide inferred speech estimates. Our method provides inferred target labels (pseudo-labels) at each timestep, alongside confidence estimates, which are used to recalibrate the cursor decoder using standard optimization approaches. This strategy allows for repeated recalibrations across time, enabling adaptation to accumulating neural changes that might otherwise prevent long-term decoder use. As we model target information using decoder outputs (instead of neural signals or latent representations in a decoding pipeline), our approach is agnostic to the choice of decoder or input signal. This approach is similar to the self-training strategies explored in previous BCI work^24,26^, where a decoder’s outputs are used to correct itself. Here we extend the framework from discrete classification^24^ and non-human primates (NHPs)^24,26^ to a continuous regression setting with a human user. Our model is inspired by retrospective target inference (RTI)^5,32^, a method that infers targets from user selections to then recalibrate velocity decoders. Here we build on this intuition by leveraging cursor behaviour in a structured, probabilistic framework that enables confidence estimates associated with model predictions across all timesteps using a hidden Markov model (HMM). Henceforth, we refer to this approach as probabilistic retrospective inference of targets (PRI-T).

Since PRI-T models target information using decoder outputs (instead of leveraging latent representations in neural data), they do not require a dimensionality bottleneck in decoder architectures that risks tossing out task-relevant information (although this may be ameliorated by more powerful dimensionality reduction techniques^27,28,33^). PRI-T also enables arbitrary retraining of multi-layer models, while neural latent space methods currently update only early components of a decoder (for instance, a single layer in FA stabilization, an autoencoder network with ADAN and an alignment network in NoMAD^28^). Finally, PRI-T is signal agnostic: since it uses structure in cursor outputs it can theoretically be deployed with BCIs that leverage other control signals, such as local field potentials^34,35^, ECoG^17,36^ and electroencephalography^37^.

To develop the approach, we used 73 recording sessions spanning five years from a participant (referred to as T5) enrolled in the BrainGate2 pilot clinical trial. In the Results, we show that PRI-T, FA stabilization^6^ and ADAN^25^ all perform equivalently when applied offline to pairs of days, but with a crucial caveat: all methods fail when day pairs are separated by large amounts of time. We further study this in large-scale simulations^38^ and demonstrate that unsupervised recalibration procedures require iterative or chained applications across subsequent sessions to maintain robust performance across time. Despite modifying FA stabilization for iterative deployment, we nevertheless find that only target-labelling strategies are able to effectively deliver long-term control in simulation; domain mapping with FA stabilization appears to accumulate error until eventually diverging. Hence, PRI-T appears similar to existing distribution alignment approaches in pairwise recalibrations, but outperforms in iterative, longer-term settings. We then use PRI-T to obtain one month of closed-loop control in participant T5 without any supervised retraining. Finally, we leverage data from a second clinical trial participant (referred to as T11) to demonstrate PRI-T’s viability in personal use settings where users are engaging in everyday tasks, such as checking emails and browsing the Internet.

## Results

### Characterization of nonstationarities across five years

We first analysed 73 closed-loop cursor control sessions from T5 to characterize the magnitude and timescale of the neural nonstationarities present in the recordings and their potential impact on decoding performance. These results set the stage for the rest of this work and inform the design of methods that aim to accommodate these nonstationarities.

**Fig. 1:**
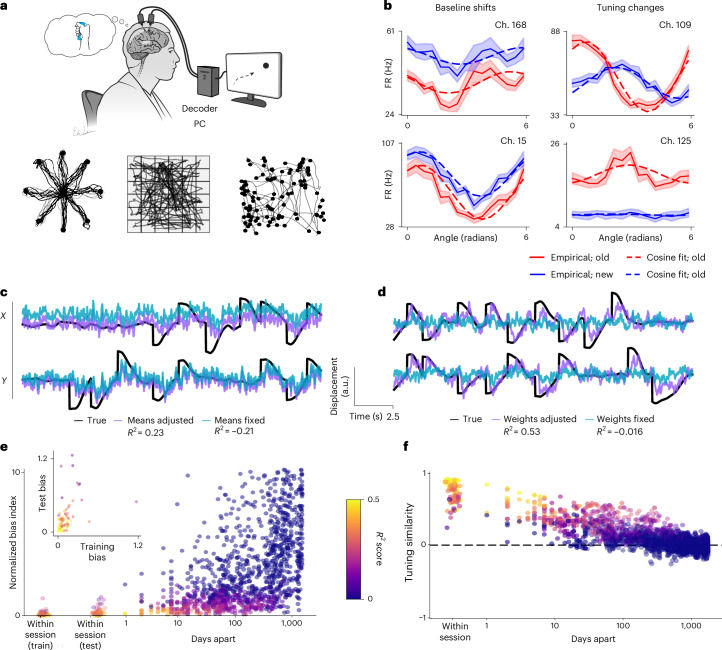
Neural nonstationarities and their impact on cursor control. **a**, During BCI cursor control, participant T5 attempted to move his hand as though controlling a joystick. Meanwhile, recorded neural activity was used to drive a velocity decoder. Cursor control was assessed with different target configurations, such as radial-8, grids and random target locations (left to right insets, respectively). Cursor trajectories are plotted for random trials from each target configuration. **b**, Example neural nonstationarities from two distinct sessions (two days apart). The dotted lines correspond to cosine tuning models fit within each session and the solid lines are empirical firing rates (FRs) for different angles with respect to the target position. The shading denotes bootstrapped empirical 95% confidence intervals (CIs). **c**, Comparison of an offline velocity decoder with and without adaptive mean subtraction. Miscalibrated mean estimates result in low performance on held-out data (*R*^2^ = −0.21). Predictions are still correlated with the ground truth, implying that weak performance is due to a strong bias rather than a tuning mismatch. After adjusting for mean shifts, the performance rises to *R*^2^ = 0.23. **d**, Performance when neural tuning has changed greatly. An old decoder fails to work (blue; *R*^2^ = −0.016) despite mean updates, whereas a new decoder works well (purple; *R*^2^ = 0.53). **e**, Bias as a function of time between decoder fitting and prediction. Each dot corresponds to a pair of sessions, where a decoder is trained on the first and deployed on a second; the new session’s data are centred using the mean estimates from the last block of the old session. Points are coloured by corresponding *R*^2^ values. On the left, we also include within-session bias estimates when using adaptive decoders on both training and holdout blocks. Bias estimates tended to rise in holdout blocks despite bias mitigation (inset; *P* < 2 × 10^−6^; two-sided Wilcoxon signed-rank test). **f**, Cosine of the angle between decoder tuning weights across pairs of sessions. Values of 1 indicate complete overlap (up to scaling), whereas 0 indicates orthogonal weights. The points are coloured by offline *R*^2^ on new sessions after mean recalibration. Credit: **a**, Erika Woodrum Art, ltd.

During these sessions, T5 attempted to move a computer cursor to a series of targets while neural activity was recorded from two 96-channel silicon microelectrode arrays (Blackrock Neurotech) implanted in the hand knob area of dorsal motor cortex (Fig. 1a). A linear decoder translated the neural activity into cursor velocity signals, thus enabling control of 2D graphical user interfaces with a computer mouse. Much like a mouse enables able-bodied users to issue discrete clicks on parts of a screen, the BCI system also enabled selections through neurally derived clicks, which are inferred from a separate decoder, or through a dwell-based approach where items are selected if the mouse hovers for sufficient time over them. In these data, both approaches for selecting buttons (targets) were present.

We first investigated two classes of neural nonstationarity: baseline shifts and preferred direction (PD) changes under cosine tuning (which captures linear tuning to the user’s intended movement direction; Methods and Fig. 1b). Baseline shifts maintain a channel’s response specificity to direction but alter its mean activity, whereas PD changes are changes in the PD angle or modulation strength of the tuning curve. Functionally, their impacts on decoder performance also differ. Mean shifts preserve correlations between the predicted and ground-truth velocity with a linear decoder when evaluated offline (see, for example, ref. ^9^ for a discussion of closed-loop effects). In an example dataset, we show how a decoder with miscalibrated mean estimates performs poorly on newer data (light blue traces in Fig. 1c; means fixed). After adjusting for the bias via updated means, the performance rises (purple traces in Fig. 1c; means adjusted). In contrast, PD changes cause the decoded predictions to become decorrelated from ground truth (Fig. 1d). Using a stale decoder (that is, a decoder from a previous day, potentially months or even years in the past) with updated means fails to save performance in the face of PD changes (light blue traces in Fig. 1d; weights fixed), whereas performance after supervised recalibration is much higher (purple traces in Fig. 1d; weights adjusted).

We examined the time courses of these changes using two functional metrics that reflect decoder performance: a normalized bias index and a tuning similarity measure (Methods). The normalized bias index is a ratio of the bias vector magnitude compared with the decoded control signal’s average magnitude when far from the target (when BCI users engage in maximal push)^38^. A value close to 0 indicates low bias, whereas values greater than 1 mean that the bias is greater than the user’s maximum effort. We measured normalized bias across all pairs of sessions by training an offline linear decoder on a reference session and then measuring its normalized bias index on a test session (Fig. 1e). We also include bias estimates within the session when using decoders on both training and holdout blocks, which had average normalized bias values of 0.118 $\pm$ 0.154 and 0.241 $\pm$ 0.259, respectively (mean $\pm$ s.d.). After 1–2 weeks, median bias values rose to around 0.79, which is high enough to render control almost impossible.

We then evaluated PD changes by examining the cosine angle between linear decoder weights on different pairs of sessions (that is, the subspace drift). This metric provides a global measure of all PD changes occurring across the neural population (Methods), with a value close to 1 when readout dimensions are highly aligned and a value close to 0 when they are orthogonal. We then plotted the cosine angle as a function of the gap between sessions (Fig. 1f). The median tuning similarity within the session was around 0.75 (a 41° difference), but dropped with 1–2 weeks of separation between sessions (71° difference). The median coefficient of determination (*R*^2^) correspondingly moved from 0.39 to 0.21, highlighting the impact of nonstationarities on downstream task performance. These results highlight how nonstationarities can quickly accumulate and impact decoder performance. Although mean shifts can easily be accounted for with adaptive offset-correction methods^5^, PD changes present a more advanced challenge to long-term robustness, which motivated us to design PRI-T.

### Probabilistic target inference via a HMM

**Fig. 2:**
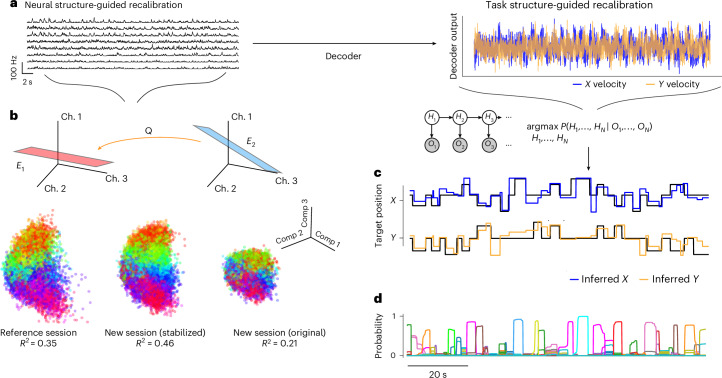
FA stabilization and PRI-T overviews. **a**, Neural firing rates on a new day are fed through an old decoder, resulting in a noisy cursor velocity command time series. **b**, FA stabilization identifies an orthogonal map *Q* that realigns a new day’s subspace *E*_2_ with the reference day, *E*_1_. State-space plots show the neural activity in top FA components, coloured by the cursor displacement angle. A decoder trained in the reference latent space provides decent performance, but drops in performance when fed data from a new session (original). Applying stabilization via *Q* increases the performance by aligning the subspaces (stabilized). **c**, PRI-T instead operates on decoder outputs, by identifying the most likely (Viterbi) sequence of targets {*H*_*t*_} given the observed velocities and cursor position {*O*_*t*_}, which closely matches the ground truth. **d**, We also query the model for its confidence in its predictions by obtaining the marginal probability of the target state *P*(*H*_*t*_ | *O*_*1*_, …, *O*_*N*_), plotted here as coloured lines (one for each possible target state), and use this to weight the importance of each pseudo-target label during recalibration.

Strategies such as FA stabilization (Fig. 2a) and ADAN aim to stabilize decoders in the face of PD changes by taking advantage of a subset of consistent neural activity patterns that may persist across days. In the case of FA stabilization, this approach involves training an FA subspace decoder on some reference day and then using a Procrustes alignment to realign the FA model from a new day (Fig. 2b).

Alternatively, one might use task-related structure to perform unsupervised recalibration (Fig. 2a, right) by inferring the user’s intended targets from noisy decoder outputs and then using those inferred targets to retrain the decoder. To optimize this process, we would ideally like a labelling model that provides both (1) pseudo-labels for retraining our cursor decoder; and (2) an associated measure of confidence in its outputs.

RTI is one such task-structure-related approach, and assumes that user selections happen when the cursor is hovering over a target^5^. Data from a preceding time window, when the user was presumably moving towards estimated target locations, are used to update the decoder after the target selection occurs. RTI is hence unsupervised, but its pseudo-labels do not have associated confidence estimates, which might be helpful for weighting the importance of a given target estimate to the overall recalibration. Furthermore, there is additional information in the cursor trajectory that cannot be leveraged by examining the user’s intended clicks alone. For example, if one observes a cursor moving steadily towards a given corner of the screen, we might infer that a user is aiming towards a target there. This provides complementary information that can be exploited for better target labelling. RTI also requires the user to be actively selecting targets to enable retrospective retraining, rendering the approach unsuitable for situations where a user might passively hover over parts of the screen, such as drop-down menus and browsing.

We leverage continuous cursor information and optionally discrete selections by linking them to the target state via PRI-T, a probabilistic model (Methods). PRI-T uses an HMM similar to that in ref. ^39^ to obtain target estimates in a principled way, enabling continuous predictions (Fig. 2c) alongside confidence estimates (Fig. 2d) that allow us to differentially weight the importance of different target estimates for recalibration (Methods and Supplementary Fig. 1). Using a probabilistic model unlocks a wider array of downstream algorithms (for example, weighted least squares) and easy integration of auxiliary signals such as click when available.

### Sensitivity of existing strategies to long-term PD changes

To benchmark PRI-T against existing approaches, we examined robustness across multiple timescales via offline analysis of session pairs. Since neural nonstationarities generally increase with larger gaps between sessions (Fig. 1e,f), it is possible that recalibration systems might perform well in the short term, when such changes are small, but fail in the long term.

**Fig. 3:**
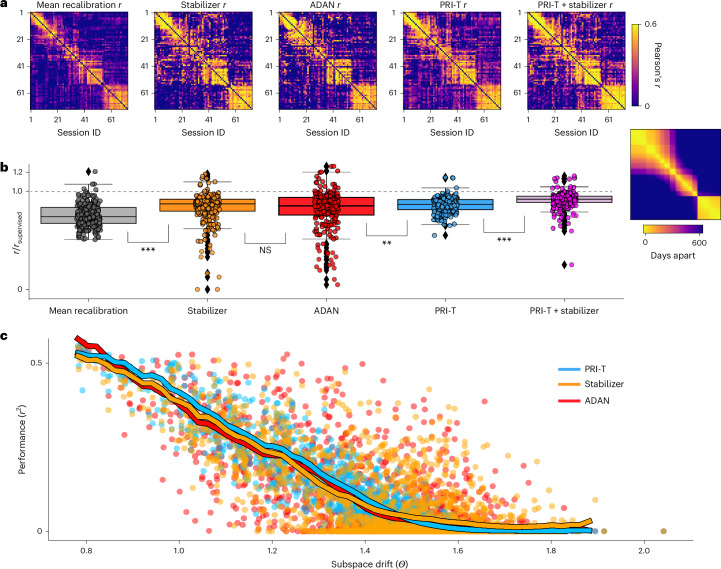
All tested methods fail with sufficient tuning drift between sessions. **a**, Performance of various approaches across different pairs of sessions, plotted as heatmaps. The colours indicate decoder output correlations with ground-truth target-minus-cursor displacement vectors after recalibration. The rightmost lower heatmap shows the time between session pairs. **b**, Box plots showing the relative performance compared with supervised recalibration (the values at the dotted line are equivalent to supervised). The central lines, box limits, whiskers and diamond points represent median values, upper and lower quartiles, 1.5× the interquartile range and outliers, respectively. Pairwise comparisons were performed using a two-sided Wilcoxon rank-sum test (*n* = 239 pairs). Differences for mean recalibration versus stabilizer (*P* < 0.001), ADAN versus PRI-T (*P* = 0.0015) and PRI-T versus PRI-T + stabilizer (*P* < 0.001) were all significant. **c**, Performance (*r*^2^; Methods) as a function of the angle in radians between supervised decoder weights on reference and new days. Solid lines correspond to moving averages (sliding window size of 0.1 and a stride of 0.02; data points were re-weighted with a Gaussian kernel with a variance of 0.5).

We analysed this possibility using a cursor control dataset from participant T5 spanning roughly five years and generated pairs of sessions (Methods) for evaluating recalibration performance. For each pair, a decoder was trained on the earlier session and tested on the newer session after separately applying five methods: supervised recalibration, mean recalibration, FA stabilization, ADAN and PRI-T (Supplementary Fig. 2 and Extended Data Table 1). Plotting performance across pairs of sessions as a heatmap (Fig. 3a; note the uneven sampling of days along the axes—an additional heatmap on the right shows the time between session indexes), we observed similar overall performance patterns across approaches. For a more direct comparison, we normalized scores against the performance of supervised recalibration and plotted the data side by side (Fig. 3b). Here we subselected days where mean recalibration alone achieved a minimum level of baseline performance (median (interquartile range) normalized *r*^2^ = 0.78 (0.70–0.87)) (Methods). All methods generally outperformed mean recalibration (*P* $<$ 0.001; Wilcoxon rank-sum test), implying that all methods were successfully accounting for some of the PD changes as well as mean changes on this subset of days. FA stabilization, ADAN and PRI-T all clustered around 0.89–0.91 in median scores, but with variable spread. Both latent space methods appeared to have more variability at the extremes, albeit with similar IQRs (normalized *r*^2^ for stabilizer = 0.83–0.96; normalized *r*^2^ for ADAN = 0.89–0.98; normalized *r*^2^ for PRI-T = 0.85–0.96). PRI-T and FA stabilization had similar median performances (*P* $=$ 0.069; Wilcoxon rank-sum test). We also tested a simple ensembling model (normalized *r*^2^ = 0.96 (0.93–0.99); Fig. 3b) that averages decoder predictions from PRI-T and FA stabilization, which provided the highest relative performance across methods (all *P* $<$ 0.001; Wilcoxon rank-sum test) in this single recalibration setting. Pairwise comparisons across methods had similar findings, with all unsupervised methods outperforming mean recalibration and otherwise appearing equivalent aside from the ensembled approach (Methods and Supplementary Fig. 3). These results were consistent when examining *R*^2^ as well (Supplementary Fig. 4).

Interestingly, all approaches had similar failures, underperforming on day pairs that were further apart in time (upper right corners in Fig. 3a) compared with when sessions were closer to one another (banding pattern along the diagonal in Fig. 3a). Since far-apart sessions probably have a much larger degree of signal nonstationarity compared with closer sessions, we reasoned that this trend may reflect an inability to compensate for long-term signal drift. We explicitly tested this hypothesis by analysing performance as a function of subspace drift. The results showed that PRI-T, ADAN and FA stabilization all perform poorly in the face of large rotations of the subspace in which neural tuning to movement exists, and seem to fail completely for rotations larger than roughly 90° (albeit with differing variability; Fig. 3c).

These initial offline findings indicate that when performing unsupervised recalibration using only a single pair of days all approaches appear unsuitable for maintaining long-term cursor control in our participant. On a shorter timescale, however, performance was somewhat more stable. We therefore reasoned that iterative recalibration approaches that use chains of days might work better to counter non-stationary neural activity. Our intuition here was that chopping up long-term neural changes into a series of smaller day-to-day changes would ultimately be more tractable, owing to the less variable differences between nearby days.

### Building a realistic closed-loop model of neural drift

To examine the hypothesis that daily, unsupervised recalibration fares better against long-term signal changes, we wanted to obtain long stretches of consistent cursor control data without major time gaps. Since we were also interested in evaluating various recalibration strategies, we required a tractable approach that did not require too much valuable session time with clinical trial participants.

Consequently, we turned to a closed-loop simulation environment to enable large-scale, systematic performance comparisons across many recalibration strategies. We used a variant of the piecewise linear model (PLM) simulator from ref. ^38^, with an additional neural tuning model, and matched SNR characteristics to T5’s neural data (Supplementary Fig. 5a–d and Methods). We also matched the overall course of subspace drift, defined as changes in the encoding subspace orientation across time, by fitting an exponential decay model to encoding subspace drift over two weeks for the *X* and *Y* subspaces separately (Supplementary Fig. 5c, left). This yielded decay constants of 0.91 d^−1^ and 0.93 d^−1^ for the *X* and *Y* subspaces, respectively. As these values are relatively close, we opted to use the lower of the two values for both *X* and *Y* subspace drift (Supplementary Fig. 5c, right).

This latter step ensures similar average drift between simulated and observed neural activity, but leaves unspecified the variance of this drift. We chose to simulate low variance in the amount of day-to-day drift, motivated by progress in next-generation BCI systems^40–42^. As channel counts scale, iBCIs will probably acquire a degree of signal redundancy that provides some protection from nonstationarities (as noted in ref. ^43^). More plainly, this idea captures the intuition that, for example, a decoder built from five channels will have more variable performance when two channels shift than a cursor decoder built from 500 channels where 200 shift. Each channel’s drift perturbs the overall encoding subspace, but their combined effect tends to wash out extreme scenarios^9,43^. This implies that high-channel-count BCI systems should exist in a regimen where consistent drift occurs across time but with limited variability, making the case for adaptive algorithms that can compensate for these changes. We demonstrate this in a toy model with a simple linear encoding system (Methods), where we show empirically and with a rough proof that variance falls off with increasing channel counts (Supplementary Fig. 5e–g).

### Only target labelling maintains long-term control in silico

**Fig. 4:**
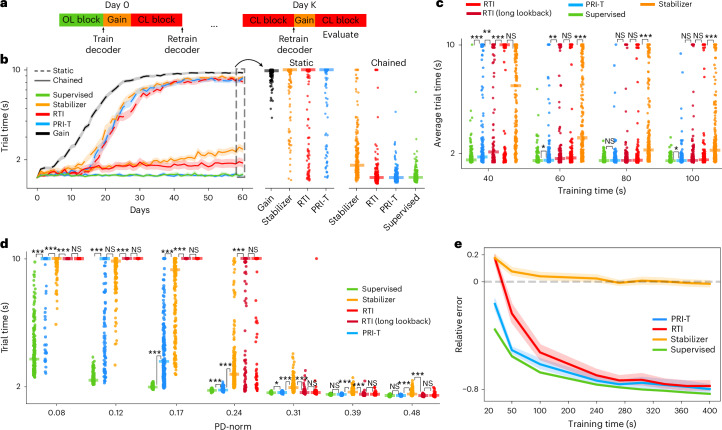
Comparison of recalibration strategies in simulation. **a**, Overview of the multiday recalibration experiment. Decoders were trained on open-loop (OL) and closed-loop (CL) data on a reference day, then tested in a closed-loop simulation on a new day. Recalibration was then applied, followed by a gain adjustment, before measuring the performance on another closed-loop block. **b**, Left, average trial times across sessions for different methods (400 s or 6.7 min of data per block). The shading corresponds to standard error over 200 independent runs. Right, comparison of trial time values between methods at 60 days out. **c**, Performance of RTI, PRI-T and supervised recalibration as a function of training data size with 100 independent runs each per data size tested (two-sided Wilcoxon rank-sum test; *P* = 0.013 for supervised versus PRI-T at 60 s; *P* = 0.0017 for PRI-T versus RTI (long lookback) at 60 s; *P* = 0.044 for supervised versus PRI-T at 100 s). All of the other tests were either significant at *P* < 0.001 (triple asterisks) or not significant (NS). The trial times were measured at 30 days out. **d**, Performance at 30 days out as a function of the simulator’s SNR (as quantified by PD-norm). We simulated 200 repetitions for each method. Statistical significance was determined by two-sided Wilcoxon rank-sum test (*P* = 0.026 for supervised versus PRI-T at PD-norm = 0.31). All other tests were either significant at *P* < 0.001 (triple asterisks) or not significant. **e**, Performance after letting stabilizer errors accumulate. Each method was then run for five days across different dataset sizes and the relative decoder error was then plotted. Lines and shaded regions represent means and bootstrapped 95% CIs.

We then simulated closed-loop control with dwell-based target selections over two months in the presence of a drifting encoding subspace. For all decoders, we optimized the cursor gain within each day (Fig. 4a) so that we could more closely measure performance related to how well the decoder tracked this subspace (versus, for example, variability arising from high-speed cursors overshooting targets; Methods). As changes compound, a decoder fit on the first day (black curve in Fig. 4b) grows increasingly noisy until it is no longer usable (mean ± s.d. trial time = 9.57 ± 0.76 s per trial at 60 days out). This breakdown happens despite gain optimization within each day, implying that poor performance is due to low-quality decoder weights. In contrast, using supervised recalibration (green curve) yields higher and more consistent performance over time with an average trial time of 1.54 $\pm$ 0.32 s at 60 days (*P* $<$ 1 × 10^−60^; two-sided Wilcoxon rank-sum test).

We also simulated three unsupervised recalibration procedures: PRI-T, RTI and FA stabilization (with hyperparameter searches for all; Supplementary Fig. 6 and Extended Data Table 2). We found that all procedures were prone to failure as time passed between the reference and new day’s data, causing poor performance at the 60-day mark (8.58 $\pm$ 2.62 s for stabilization (dotted orange line); 8.37 $\pm$ 3.09 s for RTI (dotted red line); 7.91 $\pm$ 3.36 s for PRI-T (dotted blue line); Fig. 4b). The median performance was statistically significantly better than gain optimization for RTI (*P* $<$ 0.05; Wilcoxon rank-sum test) but not for FA stabilization (*P* $=$ 0.57) or PRI-T (*P* $=$ 0.42).

To avoid this issue, we reasoned that on a short timescale drift is minimal and a sufficient number of stable channels should exist to make the alignment problem more tractable. We therefore implemented a daisy-chained FA subspace stabilizer, which leverages relatively easy day-to-day mappings to stabilize neural data with respect to the reference day (solid orange curve in Fig. 4b). Rather than mapping a new session’s activity to the original subspace, we instead learn a set of easier day-to-day maps and compose them to obtain a transformation into the original subspace (Supplementary Fig. 6a). This approach proved to be far more robust over long time spans compared with the standard stabilizer (*P* $<$ 1 × 10^−44^; Wilcoxon rank-sum test), but still yielded suboptimal control, with some sessions being totally uncontrollable (session dots with average trial time values at ~10 s; Fig. 4b) and a 2× slower average trial time than supervised retrained decoders.

In contrast, chaining PRI-T (solid blue line in Fig. 4b) and RTI (solid red line) enabled consistently stable control and outperformed daisy-chained subspace stabilization (*P* $<$ 1 × 10^−25^ for both; two-sided Wilcoxon rank-sum test). Both approaches had similar performance (1.78 $\pm$ 1.41 s for RTI and 1.50 $\pm$ 0.19 s for PRI-T; *P* $=$ 0.1) and, encouragingly, did not differ significantly from supervised recalibration at the end of the simulated 60 day stretch (*P* $>$ 0.15 for both; two-sided Wilcoxon rank-sum test). Hence, PRI-T and RTI were the only two strategies that enabled high-quality, long-term control for the simulated user.

### In silico robustness of PRI-T and RTI

As RTI and PRI-T were close in performance, we then looked at the data efficiency of each method by measuring performance after 30 days of unsupervised recalibration while varying the amount of training data provided (Fig. 4c). Both self-training methods were far more robust compared with subspace stabilization, with significantly faster trial times across all of the tested dataset sizes (*P* $<$ 0.001 for 60–100 s; Wilcoxon rank-sum test), except for the smallest dataset size. At this point, the stabilizer showed higher median performance than RTI (albeit not statistically significant), but was still worse than PRI-T (*P* $<$ 0.001). We reasoned that this may be due to RTI’s lookback window being too small, resulting in insufficient data for retraining. We therefore introduced a modified RTI with a larger lookback window (RTI (long lookback); dark red in Fig. 4c; Methods) and found that it outperformed subspace stabilization (*P* $<$ 0.001) but still fared worse than PRI-T at 40 s (*P* = 0.048).

In contrast, PRI-T resulted in lower average trial times at every dataset size compared with other unsupervised methods (Fig. 4c). PRI-T showed statistically significant gains compared with RTI at several sizes (*P* $<$ 0.05 at 40, 60 and 100 s), as well as the long lookback variant (*P* $<$ 0.05 at 40 and 60 s). Supervised training generally outperformed PRI-T (*P* $<$ 0.05 at 40, 60 and 100 s), albeit with relatively small gains. For instance, PRI-T maintained qualitatively similar control to supervised recalibration, even at an 80% reduction in the amount of training data (80-s training set duration; *P* $=$ 0.35; two-sided Wilcoxon rank-sum test).

We also considered how robust each model was to the simulated neural population’s SNR (Fig. 4d). We fixed the population tuning matrix norm at different strengths (Methods) while running the simulator for 30 days, then measuring performance on the last day. Interestingly, at a critical point, RTI worsens significantly compared with subspace stabilization and PRI-T (PD-norm = 0.24). This reveals a possible failure mode of RTI: as the SNR drops, the number of dwell-based clicks drops due to a lack of successful trials. At this point, subspace stabilization becomes preferable to RTI (*P* $<$ 0.001 up until the smallest SNR setting). However, since PRI-T uses velocity information, it is much more robust to this issue and maintains a lead over subspace stabilization (*P* $<$ 0.001, except for PD-norm = 0.12, which was not statistically significant). Note that T5’s median SNR corresponds to a PD-norm here of approximately 0.58; hence, these robustness benefits are probably more relevant for participants with worse control or lower SNR recording modalities.

We also evaluated the performance of click-augmented PRI-T (Methods), whereby an explicit click observation model is integrated into the HMM to account for dwell-based selections in the simulation. This resulted in a significant data efficiency improvement compared with the standard PRI-T model at the smallest dataset size (0.001 at 40 s; Supplementary Fig. 7a), as well as improved robustness to lower neural population SNRs (*P* $<$ 0.05 for SNRs $\le$0.31; Supplementary Fig. 7a), validating the integration of click and cursor decoder information to improve performance beyond either signal alone in this simulation environment.

### Error-accumulating nature of chained subspace stabilization

Chained subspace stabilization involves composing multiple subspace transformations together, which might introduce compounding errors that could explain the subpar performance we observed relative to target-labelling methods. To investigate this, we first ran independent simulations of chained subspace stabilization until errors reached a threshold of at least 70° misalignment between the decoding and encoding subspaces (Methods). After this point, we then considered the effects of applying different recalibration methods for different dataset quantities, to see whether any methods were capable of reversing the accumulated error when given enough training data. Here error was defined using the correlation between decoder weights and the population encoding matrix (Methods).

We found that, despite increasing the dataset size, subspace stabilization was unable to remove previous errors in the decoder estimate (Fig. 4e). At best, subspace stabilization appeared to only avoid adding new errors to the system if given large amounts of training data. In contrast, self-training strategies such as RTI and PRI-T were able to compensate for previously induced errors, driving the error far below where it started. Similar to our data efficiency sweeps (Fig. 4c), PRI-T generally outperformed RTI for very small dataset sizes (<100 s here). These results offer an explanation for why subspace stabilization fails over the long term: errors are inevitably introduced with each new update and compound over time, with no mechanism available for correcting the error.

The above results also raise an apparent contradiction: how is it that RTI and PRI-T have poor performance in the offline setting when large errors in the decoder have accumulated (Fig. 3c), but are able to drive errors to zero in the closed-loop setting even in the face of initially large decoder errors (Fig. 4e)? We believe the answer lies in a crucial difference present in the closed-loop setting. In the closed-loop setting, the user is driving the cursor towards the target with the poorly aligned decoder while using visual feedback to correct for any decoding errors that occur due to misalignment. These user corrections make the decoder output more informative of the true target location, allowing target inference methods to more accurately estimate the user’s target. As long as control quality is reasonable enough, the user will eventually reach the intended target, which can then be inferred from the decoder output. However, in the offline testbed setting, these corrections cannot be applied (given that target inference methods are only given access to the misaligned decoder outputs), leading to worse target inferences.

### Performance across one month of closed-loop control with T5

PRI-T’s superior performance in the simulation was encouraging, but nevertheless could not be assumed to generalize to real closed-loop control in T5. We therefore tested the ability of PRI-T to enable fully unsupervised, closed-loop control as T5 engaged in a random target task over one month.

During each session, the weights of a linear decoder were updated using PRI-T after each closed-loop block (see Methods for decoder details). We benchmarked PRI-T’s ability to compensate for neural changes by also comparing FA stabilization and a fixed decoder with bias subtraction to account for mean shifts (Methods). PRI-T consistently outperformed the other decoders, both near the onset of closed-loop data collection and towards the end. PRI-T’s advantage increased considerably when tuning diverged the most from the reference day. In contrast, FA stabilization was highly variable and often failed to improve on a fixed decoder (such as at day 12). However, FA stabilization outperformed the fixed decoder on the last day, when neural activity presumably reverted back to a similar pattern as on day 0, as evidenced by the improved performance of the fixed decoder between days 14 (6.83 $\pm$ 3.03 s per trial; mean $\pm$ s.d.) and 28 (3.61 $\pm$ 2.12 s).

**Fig. 5:**
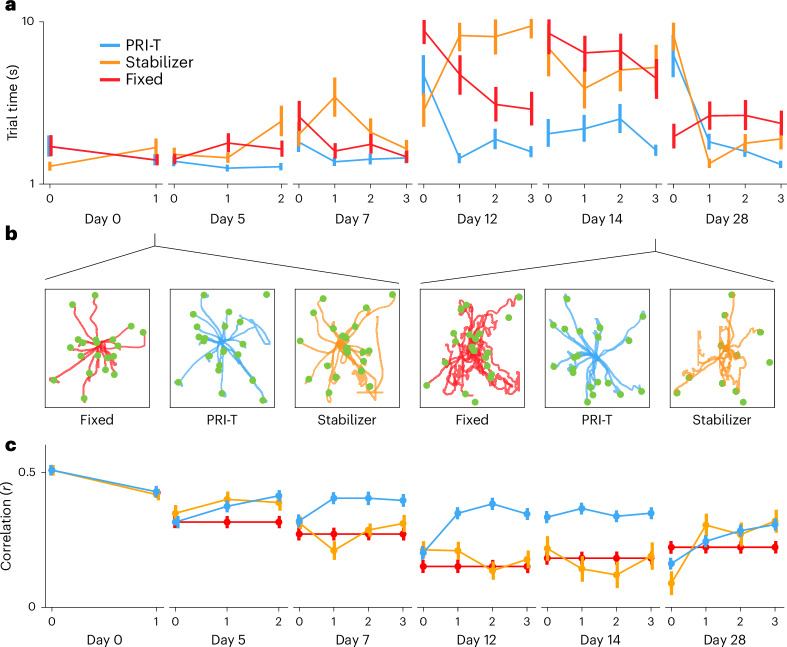
Closed-loop performance comparison. **a**, Average trial times across blocks and sessions for three different decoder strategies (bootstrapped empirical 95% confidence intervals). After initial supervised training on day 0, the decoders were tested and recalibrated repeatedly in an interleaved manner throughout a session. The ticks represent rounds of testing and track the number of previous recalibrations on a given day (for instance, day 7, slot 3 tracks performance across decoders after three previous iterations of deployment and recalibration). On a new day, the decoders from the end of the previous day were loaded for round 0. **b**, Example cursor trajectories from the first and fifth sessions for all three recalibration strategies. The green dots represent target locations. The trial starts are recentred to the origin. **c**, Open-loop block correlations for each decoder. The intervals again reflect 95% confidence intervals (empirical bootstrap; *n* = 10,000 resamples). Throughout the second week, PRI-T outperformed FA stabilization in open-loop decoding (on average, 0.14 ± 0.078 increase in correlation or 81% relative improvement).

We also collected an open-loop block at the beginning of each day to better probe decoder performance without closed-loop effects (Fig. 5c). All trends were roughly consistent, with PRI-T outperforming FA stabilization further out in time (days 7, 12 and 14). On day 28, PRI-T was similar to FA stabilization, and both methods outperformed the fixed decoder. The fixed decoder partially recovered on day 28, implying that the neural activity dimensions found on day 28 have stronger overlap with day 0 than those of the previous sessions. This is possible given that a random walk model of continuously accruing instability may be a simplification of real-world settings. Nevertheless, PRI-T appears to be the best method for adapting to nonstationarities, as for a majority of the time neural activity will differ substantially from an initial state as time progresses.

### PRI-T is more data efficient than RTI on personal use data

**Fig. 6:**
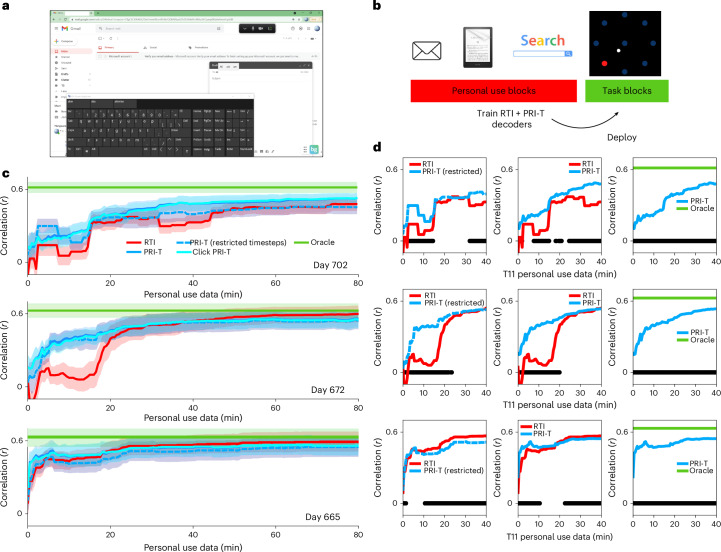
Offline evaluation of PRI-T and RTI on freeform, personal use data. **a**, Screenshot from a personal use session (day 672), during which T11 controlled a desktop computer via 2D cursor control, as well as discrete selections (Methods). **b**, Self-training evaluation procedure: training data were collected from personal use blocks, during which T11 engaged in a variety of applications, including sending emails, e-book reading and web searches. Cursor and click signals during these blocks were used to train decoders using either RTI or PRI-T, which were then evaluated offline on held-out task blocks from either the start or end of the day. **c**, Recalibrated model performance on task blocks with increasing personal use data (evaluated on non-overlapping, 30-s segments; *n* = 38, 18 and 19 distinct segments for days 665, 672 and 702, respectively). The data represent means and s.d. The green lines denote supervised baseline trained on the held-out data (cross-validated using the same segments). **d**, Mean performance for different pairs of approaches from **c**, with black scatters indicating significantly different timepoints (*P* < 0.05; two-sided Wilcoxon signed-rank test).

To further validate translational viability, we next examined PRI-T’s performance offline in unstructured, personal use settings. Here we used data from a second clinical trial participant (T11) while he performed tasks such as email typing (Fig. 6a), web browsing and e-book reading. Using cursor and click outputs from these blocks, we then trained linear decoders using RTI and PRI-T and evaluated them offline on held-out, structured task blocks (Fig. 6b). This enabled us to retrain in an unstructured setting while still obtaining a ground-truth readout for performance evaluation (via regression against a point-at-target vector during a task with known targets).

For each approach, we measured test block performance as a function of the amount of personal use training data provided (Fig. 6c). We performed a grid search (Supplementary Fig. 8 and Extended Data Table 3) separately for RTI (blue) and click PRI-T (cyan; where we augmented the HMM observation model with a click indicator variable). We also considered a supervised baseline (oracle; green) that was trained and evaluated on test blocks using cross-validation.

PRI-T was generally more data efficient than RTI across sessions, with significantly higher performance on the test data for training sizes under roughly 20 min (Figs. 6c and6d (middle), but note that day 665 showed a small but significant advantage with RTI at around 20–40 min). This data efficiency appears to be due to better pseudo-labels, as restricting PRI-T’s labels to the same timesteps as RTI’s still showed differences in performance for two of the sessions (restricted timesteps in Fig. 6d, left). Interestingly, click PRI-T’s performance curves are nearly identical to the standard PRI-T instantiation (Fig. 6c and Supplementary Fig. 8c,e,g). This is in contrast with simulation results where click was helpful for small dataset sizes (Supplementary Fig. 7a), suggesting that velocity signals alone may be sufficient for unsupervised recalibration. Encouragingly, PRI-T’s performance gap with the oracle tended to lessen—but not disappear—when provided with increasing amounts of data (Fig. 6d, right) or when using a variant without any timestep weighting (Supplementary Fig. 8g), which also generally outperformed RTI across a range of dataset sizes. These results imply that, with sufficient retraining and parameter tuning, PRI-T could be able to achieve a degree of iBCI cursor control competitive with supervised retraining in an everyday setting.

## Discussion

Our offline, in silico and online results demonstrate an unsupervised recalibration system that outperforms subspace stabilization and helps to tackle one of the remaining technical roadblocks for clinical translation of cursor BCI systems. By providing consistent control without supervision—despite compounding neural nonstationarities—these findings demonstrate a way for BCI users to maintain robust control without frequent pauses for supervised recalibration. Our work also demonstrates that iterative or chaining-based strategies may be necessary to compensate for long-term signal drift and that, at least in simulations, only target inference methods such as PRI-T and RTI appear capable of indefinite chaining.

Recent work has examined neural latent space recalibration procedures^6,25,27,28^. In studies with NHPs, these approaches align neural activity to a reference day, enabling a fixed decoder to continue performing despite accruing neural changes. However, in our offline analysis, we found that two such approaches—FA stabilization and ADAN—were unable to consistently realign activity in the face of large subspace drifts (Fig. 3c). ADAN had similar performance to FA stabilization, albeit with larger variability, which may reflect a tendency to overfit on these data (Methods). Similarly, PRI-T was unable to restore performance when subspace changes were large between reference and test sessions. We suspect that these failures reflect an inability to deal with long-term drift, where the encoding subspace has moved far away from an initial reference location. In the case of FA stabilization, the alignment step may fail if the underlying task manifold is too symmetrical to enforce a unique identification (a failure mode seen in other subspace alignment methods; for example, in refs. ^23,29^ with kinematic and neural distribution realignment, respectively). For instance, linear tuning to velocity-like signals can cause a radially symmetric distribution of neural activity within the task subspace. If few stable channels exist to constrain the Procrustes problem, the alignment may be particularly sensitive to rotations. This aligns with recent work showing that both FA stabilization and ADAN underperformed with NHP centre-out reaching data^28^. More immediately, this failure mode would explain FA stabilization’s poor performance offline on pairs of sessions with large subspace angle changes (Fig. 3), in our closed-loop simulations (Fig. 4) and during closed-loop control in T5 on day 12, which had the largest neural nonstationarities with respect to day 0 (Fig. 5). Although chaining stabilization across time may prolong performance in future closed-loop studies, our simulation results suggest that errors should accrue until control is no longer possible (Fig. 4).

Since long-term drift rendered all approaches ineffective, we next reasoned that iterative updates are required to maintain high performance. We demonstrated this in simulation using a variant of subspace stabilization (Fig. 4), where subspace mappings were fit across consecutive pairs of sessions and then chained together. Despite stability improvements, chained subspace stabilization still showed overall high variability, with only chained target inference approaches (RTI and PRI-T) enabling consistent long-term control in simulation. In real closed-loop control, applying PRI-T iteratively also enabled relatively stable performance across one month. Chaining subspace stabilizers in simulation probably failed due to compounding errors over time. If each iteration of the stabilizer introduces an independent estimate of the optimal rotation angle from day to day, the final transformation (which comprises these smaller mappings) will accumulate errors over time without a self-correction mechanism. This aligns with our simulation results that demonstrate an inability to remove previous errors on the part of subspace stabilization (Fig. 4e). That being said, FA stabilization may also benefit from consolidation of latent neural activity^11^, which could be hindered by our interleaved block design during closed-loop sessions.

By extending RTI’s self-training approach to incorporate cursor information, PRI-T enables unsupervised recalibration during periods with few active selections, such as during drop-down-menu usage and hovering, while also increasing data efficiency. This latter benefit may be especially useful after periods of inactivity (for example, at the start of the day), when neural changes have accrued enough to impair high-performance control. However, this benefit comes at the cost of increased complexity, with additional hyperparameters (including whether to use confidence weights; Methods and Supplementary Fig. 8g) compared with RTI, and an increased compute overhead to perform a Viterbi search. To optimize the number of target grid points, we recommend beginning with a 20 × 20 arrangement (as in this work with a standard display) and then scaling based on held-out performance and compute constraints.

Instability of neural signals may be greater in humans than in animal models such as NHPs. For instance, one offline NHP decoding analysis demonstrated consistent performance over hundreds of days using fixed linear decoders^44^. Yet our work shows little correlation on average between task subspaces after a few months (Fig. 1) and highly variable performance in closed-loop with FA stabilization. Given the single subject nature of the study, one hypothesis for the discrepancy is that T5’s neural signals are unusually unstable relative to those of other iBCI users. However, other work has identified rapid fall-offs in the number of stable channels across time^14^, albeit with some highly stable subsets, as well as closed-loop instability in human users^3,5^.

Although recent work has often focused on neural distribution-matching strategies, many of these are yet to be evaluated for online BCI control. One exception is FA stabilization, which was evaluated over a five-day period in NHPs^6^, and may be optimal for a specific noise regime experienced in shorter timeframes. In contrast, self-training has shown much promise, with one group^26^ demonstrating robust cursor control over 29 days in NHPs using Bayesian updates on decoder weights. Similarly, RTI has been deployed with a human iBCI participant for stable cursor control over 30 days^32^. Alongside our closed-loop results here (28 days), we believe that self-training strategies demonstrate strong potential for long-term BCI stability and are well positioned for final validation in online, personal use environments.

Strategies such as PRI-T may therefore be crucial for long-term stability in human recordings. Future efforts to quantify the extent of signal nonstationarity differences between animal models and human BCI users, as well as differences between structured tasks and personal use behaviour, may enable a more nuanced understanding of the conditions under which recalibration procedures will generalize to human users. By leveraging relatively simple task structure, unsupervised retraining procedures can simplify and stabilize long-term control for end users, aiding clinical translation of cursor BCIs.

## Methods

Research sessions were conducted with volunteer participants enrolled in the BrainGate2 pilot clinical trial (ClinicalTrials.gov ID: NCT00912041). The trial is conducted under an Investigational Device Exemption provided by the US Food and Drug Administration (Caution—Investigational Device, Limited by Federal Law to Investigational Use) and is approved by the institutional review boards of Stanford University Medical Center (protocol 52060), Brown University (0809992560) and Mass General Brigham (2009P000505 and 2011P001036) and the Department of Veterans Affairs Providence Institutional Review Board (1633692).

Participant T5 was a right-handed man who was 69 years old at the time of the study. He was diagnosed with C4 American Spinal Injury Association Impairment Scale grade C (AIS-C) spinal cord injury (SCI) 11 years before this study. T5 was able to speak and move his head and has residual movement of his left biceps, as well as trace movement in most muscle groups. T11 is a 37-year-old right-handed male with a C4 AIS-B SCI that occurred approximately 11 years before study enrolment. Both T5 and T11 gave informed consent for this research and associated publications.

Throughout this manuscript, single, double and triple asterisks indicate *P* values of <0.05, <0.01 and <0.001, respectively.

### Experimental setup and neural recordings

We obtained 73 separate sessions worth of data from participant T5 spanning five years (2016–2021). Although some of these data have been previously reported^3,45^, many sessions are from monthly cursor tasks intended to monitor long-term device viability. These sessions comprise multiple tasks (radial-8, grid and random; Fig. 1a), but all share a 2D cursor-to-target acquisition structure. In radial-8 and grid tasks, the screen contains a set of equally sized targets to select from, whereas the random task contains a single, often variably sized target. The goal or active target in these tasks is often predetermined in advance (radial-8 and random), but these data also include free response settings (for instance, from ref. ^3^).

From T5’s historical cursor control data, we selected sessions with $\ge$2 blocks of closed-loop cursor control recordings each. Spikes were identified based on threshold crossings at −4.5 multiplied by the root mean square. The resulting neural time series from each session were downsampled to 1,000 Hz and then binned in non-overlapping, 20-ms bins to generate firing rate estimates. These estimates were further smoothed with a causal half-Gaussian filter (*σ* = 40 ms) to denoise the signals, resulting in a 192-dimensional vector $\mathbf{x}_{t}\in {{\mathbb{R}}}^{192}$ of population firing rates at each timestep *t*.

For all analyses aside from cosine tuning models (below), we also performed blockwise mean subtraction to centre the firing rates.

### Cosine tuning models

Cosine tuning curves^46^ were fit to each channel by regressing the instantaneous cursor angle *θ*_*t*_ (with respect to the target) against that channel’s firing rate *x*_*t*_. Cosine tuning is a standard model in the field that captures directional tuning, which has been shown to be a large signal in the motor cortex^46–48^. The standard cosine tuning model ${x}_{t}={b}_{0}+a\cos \left({\theta }_{p}-{\theta }_{t}\right)$, where *θ*_*p*_ is the PD and *θ*_*t*_ is the instantaneous velocity angle, is equivalent to a linear model of activity, ${x}_{t}={b}_{0}+{b}_{1}\cos \left({\theta }_{t}\right)+{b}_{2}\sin \left({\theta }_{{t}}\right)$, where ${b}_{1}=a\cos \left({\theta }_{{p}}\right)$ and ${b}_{2}=a\sin \left({\theta }_{p}\right)$. We hence used standard least squares to fit models by regressing neural firing rates against the ground-truth (unit) displacement vector.

Ground-truth firing rate plots (Fig. 1b) were obtained by first binning observed cursor angles into 15 evenly spaced, non-overlapping bins from 0 to 2π radians. The corresponding firing rates at each timestep were then averaged for each trial × bin × channel. We then generated bootstrapped mean estimates by sampling with replacement across trials to obtain 95% confidence intervals for each channel and bin angle. Trials with fewer than ten timepoints (200 ms) in a given bin were excluded from the sampling procedure.

### Measuring drift in T5’s recordings

For the offline analyses in Fig. 1, we fit and evaluated ridge regression models within each session by regressing the instantaneous displacement vector ($\mathbf{y}_{t}=\mathbf{g}_{t}-\mathbf{p}_{t}$, where **g**_*t*_ is the target location at timestep *t* and **p**_*t*_ is the cursor position) against population firing rates in a 50:50 train:test split across blocks. We swept L2 regularization strengths using fivefold cross-validation and then retrained models on the full training set using the optimal value. We then measured performance using *R*^2^ on the test set.

### Bias

To measure bias across time from old decoders, we used a normalized bias index that takes into account the within-day SNR. Specifically, we modelled a decoder’s outputs ${\hat{\mathbf{y}}}_{t}$ on the test set as a noisy linear readout of the true (unit) displacement vector **y**_*t*_, specifically ${\hat{\mathbf{y}}}_{t}=c\mathbf{y}_{t}+\mathbf{b}+\mathbf{\varepsilon}$, where *c* is a constant and the other terms are 2D vectors. In this equation, **ε** is noise, **b** is the degree of bias in the signal and *c* measures the signal since it reflects the degree to which decoder outputs lay along the ground-truth displacement vector (after centring). We fit this equation with least squares. If we now take the ratio ${||\mathbf{b}||}/c$, we get a normalized measure that takes into account both the bias on a given day and the tuning strength. This enables us to differentiate between days when bias is equivalent in magnitude but different in impact due to varying SNR (for example, a high SNR system, where *c* is large, will drown out a rather small bias term). In practice, we fit this model to timepoints for which the cursor was far from the target ($>$300 pixels) to create a more interpretable final number; by measuring the decoder output far from the target, we obtained a value of *c*, reflecting maximal neural push. A bias index above 1 therefore indicates that the decoder bias is so strong that even high-velocity signals are drowned out by the bias magnitude.

As we are modelling outputs during closed-loop control, one confounder is the possibility of within-session bias in the ground-truth data. This is problematic because an unbiased, high SNR decoder would have a large bias index in such a case despite being well calibrated. We adjusted for this by measuring the decoder’s output bias after removing the empirical bias **b**_0_ and obtained a final formula ${||\mathbf{b}}-\mathbf{b}_{0}{||}/c$.

When plotting bias values in Fig. 1e, we cap the *y* axis upper limit at 10 to better highlight trends. Similarly, in the inset we cap the *x* and *y* axis upper limits at 1.2 for clarity (one outlier session had sudden, brief electrical noise that massively inflated the bias estimate for that day; this session was removed from the within-day bias estimates and testing as well).

### Tuning similarity

To measure tuning similarity, we examined the cosine angle between the weights of ridge regression models after collapsing across the *X* and *Y* dimension coefficients. For instance, for decoder weight matrices ${D}_{1},{D}_{2}\in {R}^{192\,{\rm{x}}\,2}$, we compute:

$$\cos \left(\theta \right)=\frac{{D}_{1}{\left[\vdots \right]}^{T}{D}_{2}\left[\vdots \right]}{{|\left|{D}_{1}\right||}_{{\rm{F}}}{{||}{D}_{2}{||}}_{{\rm{F}}}}$$

Where ${{||}\bullet {||}}_{{\rm{F}}}$ is the Frobenius norm. A value of 1 indicates complete overlap in the readout dimensions, whereas a value of 0 indicates that the planes are orthogonal. For within-session drift, we trained a decoder on the test set while following the same cross-validated regularization procedure as earlier and took the cosine angle of its weights against the training set decoder.

### High channel count systems

Here we explain the motivation for simulating a consistent, low-variance regimen of neural changes across time, which is what would probably happen in a commercially available, high-channel-count iBCI system.

We consider a simple encoding system where the firing rate vector **x**_*t*_ across *k* channels is a noisy linear function of the intended velocity signal **v**_*t*_ (that is, $\mathbf{x}_{t}\approx {E}\mathbf{v}_{t}$) and representational drift occurs via random walk noise across days that preserve the tuning weights norm (namely, ${E}^{\prime} =\mathrm{Renormalize}\left({\rm{\alpha }}{E}+\sqrt{1-{{\rm{\alpha }}}^{2}}{P}\right)$, where ${P}_{i,\,j}\sim N\left(0,{{\rm{\sigma }}}^{2}\right)$), ${E}^{\prime}$ is renormalized to have the same column norms as *E*, and α < 1. As the encoding subspace ${E}^{\prime}$ drifts away through compounding noise, we might ask how the decoding weight *D* aligns with this new subspace.

We can compactly represent this information with a distortion matrix ${D}^{T}{E}^{\prime}$, which holds the *X* and *Y* subspace overlap on the diagonal and collisions between the *X*-encoding and *Y*-decoding subspaces on the off-diagonal (and vice versa). As the number of channels *k* scales, we find that the variance of these terms drops off like 1/*k* and ${D}^{T}{E}^{\prime} \to \alpha {I}$, using a rough proof (see below), as well as simulations (Supplementary Fig. 5). This finding leverages the observation that high-dimensional random vectors are nearly orthogonal to each other; as the number of channels increases, the random walk drift becomes less prominent within the task subspace.

### Proof sketch

Suppose our recordings measure from *k* neurons with an associated encoding matrix ${E}\in {R}^{k\times 2}$, where row ${E}_{i,\,\vdots }$ contains the *x* and *y* velocity tuning coefficients of neuron *i*. We denote the columns *E*_*x*_ and *E*_*y*_, which are linear subspaces within which the population encodings of the velocity signals lie. We further assume that ${||}{E}_{x}{||}={||}{E}_{y}{||}$ and ${E}_{x}\perp {E}_{y}$ and we then construct a decoder ${D}=\frac{{E}^{T}}{{{||}{E}_{x}{||}}^{2}}$. The former two assumptions say that the encoding of horizontal and vertical movements should have the same SNR and be encoded in separate subspaces. The latter assumption supposes that we have sufficient data and is in fact the optimal linear estimator (OLE) solution as the number of channels with uniformly distributed PDs approaches infinity^49^.

Now suppose that this population encoding drifts according to a decay model ${E}^{\prime} =\mathrm{Renormalize}\left(\alpha {E}+\sqrt{1-{\alpha }^{2}}{P}\right)$, where α is a shrinkage factor reflecting diminished tuning in the original subspace and ${P}_{i,j}\sim N\left(0,{\sigma }^{2}\right)$ is some random new component to the tuning. Then, we have that:

$${E}\left[{D}^{T}{E}^{{\prime} }\right]=\alpha {D}^{T}{E}+\sqrt{1-{\alpha }^{2}}0=\alpha {I}$$

$$\mathrm{Var}{\left[{D}^{T}{E}^{{\prime} }\right]}_{i,\,j}={\alpha }^{2}\mathrm{Var}\left[{\left({D}^{T}{E}\right)}_{i,\,j}\right]+\left(1-{\alpha }^{2}\right)\mathrm{Var}{\left[{{{D}}}^{T}{{P}}\right]}_{i,\,j}$$

$$=0+\left(1-{\alpha }^{2}\right)\mathrm{Var}\left[{\left(\frac{1}{{{||}{E}_{x}{||}}^{2}}{E}^{T}{P}\right)}_{i,\,j}\right]=\left(1-{\alpha }^{2}\right)\frac{\mathrm{Var}\left[{E}_{:,i}^{T}{P}_{:,\,j}\right]}{{\mathrm{||}{E}_{:,i}\mathrm{||}}^{4}}$$

We note that ${E}_{:,i}^{T}{P}_{:,\,j}\sim N\left(0,{{||}{E}_{:,i}{||}}^{2}{{\rm{\sigma }}}^{2}\right)$, as the sum of independently distributed Gaussians, is also normally distributed with a mean (and variance) equal to the sum of the means (and variances) of the independent Gaussians. Hence, we obtain a simplified form for the variance:

$$\mathrm{Var}\left[{\left({D}^{T}{E}\right)}_{i,\,j}\right]=\frac{\left(1-{a}^{2}\right){\sigma }^{2}}{{{||}{E}_{\vdots ,i}{||}}^{2}}$$

Note that as the number of channels grows, the denominator also increases on average, which implies that the variance diminishes with increasingly high channel counts. If we suppose that ${E}_{n,i}\sim N\left(0,{{\rm{\sigma }}}_{c}^{2}\right)$ (for some neuron *n* and kinematic dimension *i*), then ${{||}{E}_{\vdots ,i}{||}}^{2}=k{{\rm{\sigma }}}_{c}^{2}$ on average as ${E}_{n,i}^{2}\sim {X}_{1}^{2}$. So we find that, with all else equal, the variance falls off like $1/k$ for increasingly high channel count systems.

### Simulating distortion matrices

We simulated random tuning shifts 1,000 times for different channel counts. On each iteration, we randomly sampled PDs for each channel from the unit sphere, then scaled the resulting population tuning columns to have unit norm; we take the transpose of this matrix as the decoder *D*. We then generate a random perturbation vector *P* with independent and identically distributed elements (with the same sampling as PD weights and the same norm as *E*) and average its weights together with the original weights *E*, followed by column renormalization, via ${E}^{\prime} =\mathrm{Renormalize}{\rm{(}}{\rm{\alpha }}{E}+\sqrt{1-{{\rm{\alpha }}}^{2}}{P}{\rm{)}}$. We used $\alpha =0.8$ in practice for the results in Supplementary Fig. 5. Finally, we measure ${D}^{T}{E}^{\prime}$ to obtain the overlap between the original decoder and the new encoding subspace.

### PRI-T

We model the cursor position **p**_*t*_ and velocity **v**_*t*_ (collectively, observations *O*_*t*_) at some timestep *t* as a reflection of the target position using a HMM. This has three components: (1) a description $P\left({H}_{t}|{H}_{t-1}\right)$ of how the target location evolves over time; (2) a posterior distribution $P\left({O}_{t}|{H}_{t}\right)$ over the observations with respect to a proposed target location; and (3) a prior probability $P\left({H}_{t}\right)$ of the target location.

To model the target evolution across time, we want to maintain generality across cursor tasks while broadly capturing task structure to support PRI-T’s performance. Additionally, we require the target to behave according to discrete first-order Markov dynamics; this assumption means that the current (discrete) target location is simply a function of the previous state and helps to enable fast, exact inference. We accomplish this by discretizing the screen into an *N* × *N* grid and modelling the probability of moving from one grid position to another via a transition matrix *T* with elements:

$${T}_{i,\,j}=P\left({H}_{t}={h}_{i}{\rm{| }}{H}_{t-1}={h}_{j}\right)=\left\{\begin{array}{cc}\varepsilon & {\rm{i}}={\rm{j}}\\ \frac{1-\varepsilon }{N^2-1} & {\rm{i}}\ne {\rm{j}}\end{array}\right.$$

This expression says that the target has some probability *ε* of remaining in the current location and a uniform probability of transitioning to any other location. The latter decision provides task generality as we do not assume knowledge of how the target location varies in any detail; for specific tasks such as keyboard typing, one can leverage letter bigram probabilities here to improve within-task performance, although we opted for a general approach. For the screen discretization, we used *N* = 20 for all experiments. We used *ε* = 0.999 to reflect the timescale on which the HMM operates; trials were on the order of one second, whereas timesteps were 20 ms. In summary, the distribution encodes a few assumptions: (1) the current target location is just a function of the previous target location; (2) the target is likely to remain in its current spot for a while; and (3) all other possible spots are equally probable.

To model the observed cursor state as a function of the target location, we consider a Von Mises distribution over cursor velocity angles. The intuition here is that the cursor angle ${\theta }_{{v}_{t}}$ with respect to the current target should be concentrated around 0°. This provides an initial posterior of the form $P\left({O}_{t}|{H}_{t}\right)=\mathrm{VonMises}\left({{\rm{\theta }}}_{{v}_{t}};\kappa \right)$, where *κ* controls the concentration of the distribution around its mean and reflects the noise in the decoder’s angular outputs. We improve on this initial description by noting that the variability of the cursor’s angle with respect to the target will increase when the cursor is near the target, as small changes in position cause large changes in angle. To accommodate this effect, we parametrize *κ* as a function of the cursor-to-target distance,

$${d}_{{H}_{t}}={||}\mathbf{p}_{t}-{H}_{t}{||}{:}{\kappa }_{{d}_{{H}_{t}}}{=}\frac{\kappa_{0}}{1+{e}^{{-}\beta {(}{d}_{{H}_{t}}{-}{d}_{0}{)}}}$$

Here we weight an initial kappa value *κ*_0_ by a logistic function; at large distances, the effective *κ* is close to this value. However, at smaller distances, kappa is closer to 0. This causes a higher variance in the Von Mises distribution, which means that noisier velocity angles are likely when near a target. The exponent and midpoint variables *β* and *d*_0_ are found via an exhaustive grid search (Supplementary Fig. 2a). Our final posterior is then:

$$P\left({O}_{t}=\mathbf{v}_{t},\mathbf{p}_{t}|{H}_{t}={h}_{j}\right)=\mathrm{VonMises}\left({{\rm{\theta }}}_{{\nu }_{t}}|{\kappa }_{{d}_{{H}_{t}}}\right)$$

Finally, we require a prior probability over the possible target states. This distribution $P({H}_{t})$ reflects our a-priori belief about the target location and can be used to encode task-specific regularities. For instance, if one wanted to optimize for keyboard typing, this could be the empirical distribution of starting letters across all words. Like earlier, we forgo these sorts of optimizations to ensure generality across cursor tasks and instead use a uniform prior across all possible locations.

During hyperparameter sweeps, we found that using weighted least squares to emphasize timepoints based on how confident the model was about the target location was superior to using all timepoints equally or applying a discrete cutoff in T5’s offline data (Supplementary Fig. 1a). Note however that confidence weighting was strictly worse in T11’s personal use data (Supplementary Fig. 8g), implying that this setting may be participant or task dependent and should be evaluated in future PRI-T implementations.

We also saw that PRI-T’s Viterbi probabilities given by different hyperparameter settings were correlated with the resultant decoders’ offline performance on a new day (Supplementary Fig. 1b,c; mean *r* = 0.50), meaning that hyperparameters that appeared to offer a better explanation of the data also tended to train better decoders. We therefore explored an automated tuning approach whereby the hyperparameters that maximized the Viterbi probability were selected. This approach was slightly worse than the full optimization (Supplementary Fig. 1d; Δ*r* = −0.016 $\pm$ 0.036; mean $\pm$ s.d.), although not statistically significant when restricted to independent samples (*P* $=$ 0.20; two-sided Wilcoxon signed-rank test) and performed better than expected by chance (Supplementary Fig. 1e; *P* $=$ 0.001; two-sided shuffle test).

### Click integration

We can integrate click decoder outputs through an indicator variable *c*_*t*_, which records whether or not a click occurred. To model this probabilistically, we suppose that clicks have a higher likelihood of occurring when the cursor is near the target:

$$P\left({c}_{t}|{H}_{t}\right)=\mathrm{Bernoulli}\left({c}_{t}{;f}\left({{||}\mathbf{p}_{t}-{H}_{t}{||}}_{2}\right)\right)$$

where $f\left(\cdot \right)$ is fit to the empirical click likelihood as a function of the target distance from historical sessions. We then multiply this probability with the Von Mises probability, yielding the overall posterior probability over the observations ${O}_{t}\{\mathbf{p}_{t},\mathbf{v}_{t},{c}_{t}\}$. For the simulation in Supplementary Fig. 7, we set:

$${p}_{\mathrm{Bermoulli}}=f\left(x\right)=1-\frac{1}{1-{e}^{-{\left(x-0.1\right)}^{12}}}$$

For the offline T11 analysis in Fig. 6, we instead use a simpler form:

$${p}_{{\rm{Bernoulli}}}=f\left(x\right)=\left\{\begin{array}{cc}a & x < b\\ 0 & x\ge b\end{array}\right.$$

and perform a grid search over values of $$a=\{\mathrm{0.10,0.15,0.20,0.25,0.30}\},$$$$b=\{\mathrm{0.05,0.10,0.15,0.20}\}$$. We select the combination that yields the highest performance on the holdout task blocks (see ‘Offline personal use sessions with T11’ section). Here we explicitly give click PRI-T the best possible chance of outperforming the standard PRI-T integration, but still find that the two are largely the same.

### Inference

Using the HMM structure, we can then infer the most likely target sequence given the data $P\left({H}_{1},\ldots ,{H}_{n},|,{O}_{1},\ldots ,{O}_{n}\right)$. Note that this expression is an inversion of the posterior distribution earlier, as here we now measure the likelihood of the target locations given the observed data. To do so, we can perform exact inference for the most likely sequence of target locations using the Viterbi search algorithm^50^. This algorithm’s complexity is linear in sequence length, allowing for relatively fast computation. We also make use of the occupation probabilities during inference; these are the marginal probabilities of the target being in a given state at a given timestep (given the observed data). These values are obtained via the forward–backward algorithm (also linear in sequence length) and used to weight the Viterbi labels. In practice, we use the square of the maximal occupation probability at a given timestep as a weight for weighted regression: $\mathop{\max }\limits_{h}P{\left({H}_{t}=h,|,{O}_{1},\ldots ,{O}_{n}\right)}^{2}$. This latter sequence of probabilities can yield sequences that differ from the Viterbi sequence but during initial testing noticeably improved performance.

### Decoder update

By running PRI-T on past cursor behaviour, we obtain cursor-to-target intention estimates ${\hat{\mathbf{y}}}_{t}={\hat{H}}_{t}-\mathbf{p}_{t}$ and confidence estimates ${w}_{t}={\mathop{\max }\limits_{h}P\left({H}_{t}={h|}{O}_{1},\ldots ,{O}_{n}\right)}^{2}$. Associated with these time series are corresponding neural data $\mathbf{x}_{t}\in {{\mathbb{R}}}^{193}$ containing the firing rate of each channel at timestep *t* and an extra constant term in the first position (for the intercept). We then perform weighted least squares, where we find linear regression weights $\hat{{D}}\in {{\mathbb{R}}}^{2\times 193}$ that minimize the (weighted) mean squared error loss:

$$\hat{{D}}=\mathop{\min }\limits_{{D}}\mathop{\sum }\limits_{t}{w}_{t}{{||}\,\widehat{{{\mathbf{y}}}_{{t}}}-{D}^{T}\mathbf{x}_{t}{||}}^{2}$$

This expression can be evaluated in closed form or with standard, out-of-the-box numerical solvers (we use SciPy’s linalg.lstq routine, which calls a LAPACK driver^51^).

### RTI

We implemented RTI as described in ref. ^5^ for closed-loop simulation analyses. For each registered selection, we take a time window of length *L* (lookback in hyperparameter sweeps) before that click and assign the cursor position during the selection as the intended target. The cursor-to-(inferred)-target displacement signal is then regressed against neural activity from these time windows as well.

As in ref. ^5^, we apply several heuristics to improve the performance of this retrospective labelling. First, if the labelling window extends into a previous labelling window associated with a different selection, we avoid overwriting those overlapping timesteps. Second, we remove timesteps from the window when the cursor is too close in space (the minimum distance threshold in hyperparameter sweeps) or time (the minimum time) to the inferred target. Finally, we remove timesteps for which the distance function derivative is non-negative (that is, when the cursor is not actively moving closer to the inferred target).

We also considered a long lookback variant of RTI for some of the simulation analyses (Fig. 4). Here we extended the lookback to 500 timesteps (10 s), which is the maximum trial length.

### FA stabilization

In ref. ^6^, the authors use a low-rank matrix decomposition of neural activity, ${X}={LF}+{{\varepsilon }}$, where ${L}\in {{\mathbb{R}}}^{d\times k}$ is the loading matrix and ${F}\in {{\mathbb{R}}}^{k\times n}$ is a low-dimensional representation of the data. Here *d* is the dimensionality of the neural recordings, *k* is latent space dimensionality and *n* is the number of data points used. This decomposition is performed via FA in our offline results and via principal component analysis in our simulations; during the initial experiments, we saw that principal component analysis tended to provide better results for FA stabilization than FA itself. A velocity decoder was then trained in the subspace *L* by regressing velocity against the timepoints ${F}_{:,t}$. To recalibrate across days, a Procrustes alignment realigns the loadings matrices *L*^(1)^ and *L*^(2)^. That is, a matrix *O* is found such that

$${O}={\mathrm{argmin}}_{{O}^{T}{O}={I}}{||}{L}^{\left(1\right)}-{O}{L}^{\left(2\right)}{||}$$

To make the Procrustes alignment tractable, a channel selection algorithm constrains the optimization to a subset *S* of stable channels^6^. This selection algorithm uses an initial minimal stability threshold (threshold in hyperparameter sweeps) to prune noisy channels that have an L2 difference between their loading weights greater than this value. The algorithm then iteratively prunes the remaining channels until a requested number of stable channels remains (B in hyperparameter sweeps). The full alignment is then

$${O}={{\rm{argmin}}}_{{O}^{T}{O}={I}}{||}{L}_{S,:}^{\left(1\right)}-{{OL}}_{S,:}^{\left(2\right)}{||}$$

where *S* indexes stable channels.

### Daisy chaining

As drift compounds, fewer stable channels exist and this mapping becomes increasingly difficult. We avoid this problem by dividing the alignment into a series of subproblems: aligning between consecutive days when drift is small. To do so, we first initialize the stabilizer as in ref. ^6^ and obtain ${O}_{2}={{\rm{argmin}}}_{{O}^{T}{O}={I}}{||}{L}_{S,:}^{\left(1\right)}-{{OL}}_{S,:}^{\left(2\right)}{||}$. On a new day, we then repeat the channel selection algorithm and Procrustes alignment, obtaining ${O}_{3}={{\rm{argmin}}}_{{O}^{T}{O}={I}}{||}{L}_{S,:}^{\left(2\right)}-{{OL}}_{S,:}^{\left(3\right)}{||}$. We can then compose these transformations to obtain ${O}:= {O}_{2}{O}_{3}$. This process is run again for each subsequent day.

### ADAN

During the initial within-day model training, we found that the original ADAN architecture tended to heavily overfit to behavioural task data. We therefore introduced two changes to combat this problem. First, we swapped out the RNN task predictor for a ten-dimensional fully connected layer. Second, we introduced multiple data augmentation strategies to improve noise robustness and combat overfitting. This was accomplished by adding constant channel offsets, per-timestep white noise and random walk noise to each minibatch:

$$\begin{array}{c}{x}_{t}={x}_{t}+c+{\varepsilon }_{t}+{\nu }_{t}\\ {v}_{t}={\nu }_{t-1}+{\sigma }_{t}\end{array}$$

where $c,{\varepsilon }_{t},{\sigma }_{t}\sim N\left(0,q\right)$. We used *q* = 3, 14 and 0.9, respectively. Models were trained for 50 epochs with an Adam optimizer (learning rate = 1 × 10^−3^) and minibatch size of 64.

Across-day alignment hyperparameters were tuned through a grid search (Supplementary Fig. 2c) over batch size, generator learning rate and discriminator learning rate based on the results from ref. ^27^ demonstrating performance being especially sensitive to these variables. Because of computing requirements (27 parameter sets at ~ 1 h of wall-clock time each = 27 h per session pair), we used a subset of 30 session pairs for sweeps. During the initial analyses, we observed that the results tended not to converge for small epoch sizes; we consequently used 200 epochs as in ref. ^28^. We otherwise used default hyperparameters.

### Offline hyperparameter sweeps

We performed exhaustive grid searches for PRI-T, FA stabilization and ADAN over pairs of sessions. Since all methods tended to fail on sessions with large tuning changes (Fig. 3c), we opted to subselect session pairs where a mean recalibration baseline satisfied a threshold of ${r}_{\text{mean recal}} > \sqrt{0.15}$. Hyperparameters that maximized the mean performance across session pairs were then selected. In Extended Data Table 1, we show the hyperparameter values tested and corresponding data in Supplementary Fig. 2 for all three methods.

### Offline comparisons

We generated offline datasets by creating pairs of cursor sessions, with the first item in the pair being a reference session for decoder training and the second being an evaluation session. These data were processed as before (causal Gaussian smoothing with *σ* = 40 ms, 20-ms binned firing rates and blockwise mean subtraction). Within each day, we performed a 50:50 train:test split across blocks. Sessions with at least two blocks of cursor control data were used.

For examining recalibration, decoders were recalibrated on a new session’s train set and evaluated on the test set. We measured the Pearson correlation across both outputs (*r*) as well as *R*^2^, uniformly averaging over *x* and *y* dimensions. Although *r* and *R*^2^ scores showed a strong, monotonic relationship, we opted for *r* as it reflects the strength of the relationship between decoder outputs and ground truth after accounting for a difference in means and scaling. The former is modified by online mean subtraction algorithms during closed-loop control and the latter is often dealt with via simple gain scaling procedures that are also modified for optimal control. In Fig. 3c, we plot *r*^2^, which is an upper bound on *R*^2^ assuming that gain and bias are controlled for. Here we subselect for days where supervised recalibration achieved an *r*^2^ of at least 0.15 to avoid the inclusion of sessions with electrical artefacts or overall poor control. We obtained the moving average trend lines by averaging points in a 0.1-radian window with Gaussian weighting (standard deviation = 0.5). This process starts centred at the leftmost point, then iterates rightward with a step size of 0.02 until the centre passes the rightmost point. We removed outliers with a radian value > 1.9.

In Fig. 3b, we took care to subselect days for which mean recalibration had high performance. This reduces the within-approach variance by removing far-apart sessions for which all methods tend to fail. Despite thresholding pairs based on mean recalibration (*r*^2^ of at least 0.15; 239 pairs remaining), all methods nonetheless outperformed mean recalibration. We applied this same thresholding to pairwise comparisons in Supplementary Figs. 3 and 4, as well as removal of an outlier day with sudden electrical noise.

We also looked more directly at pairwise comparisons (Supplementary Fig. 3). All trends were roughly consistent with the above initial analyses. PRI-T, ADAN and FA stabilization outperformed mean recalibration (>75% of cases for all three; Fig. 4a) and with no major differences in median performance; among pairwise comparisons of the three, the largest improvement was a median 1.7% increase in scores using PRI-T over ADAN (Supplementary Fig. 3b). Using a combined approach was slightly worse than supervised recalibration (4% improvement with supervised recalibration; Supplementary Fig. 3c) but better than PRI-T or FA stabilization alone (>85% of cases). Similar results were obtained when displaying *R*^2^ as well (Supplementary Fig. 4).

### Closed-loop simulator

The closed-loop simulator here is a variant of the PLM simulator described in ref. ^38^, but with an additional neural tuning model and simplified noise. A control policy generates a neural command signal **c**_*t*_ at each 20-ms timestep in response to delayed visual feedback that pushes the cursor towards the target:

$$\mathbf{c}_{t}=\frac{\mathbf{g}_{t}-{\hat{\mathbf{p}}}_{t}}{\mathrm{||}\mathbf{g}_{t}-{\hat{\mathbf{p}}}_{t}\mathrm{||}}{f}_{\mathrm{targ}}\left(\mathrm{||}\mathbf{g}_{t}-{\hat{\mathbf{p}}}_{t}\mathrm{||}\right)$$

where **g**_*t*_ is the current target location and *f*_targ_ weights the magnitude of **c**_*t*_ depending on the distance from the cursor to the target. The simulated user’s internal estimate of the cursor position ${\hat{\mathbf{p}}}_{t}$ is obtained by forward integrating from the ground-truth cursor position 200 ms ago, using the previous command signals and ground-truth smoothing and gain^38^.

The neural command *c*_*t*_ is encoded across *k* channels with linear tuning to its *x* and *y* components (uniformly sampled PDs) to simulate neural activity **x**_*t*_ at timestep *t*. Neural activity is then corrupted by independent, mean-zero Gaussian noise (*σ* = 0.3) and linearly decoded at each timepoint, yielding a noisy velocity command signal **ŷ**_*t*_:

$$\begin{array}{l}{\mathbf{x}}_{t}={E}\mathbf{c}_{t}+N\left(0,{\sigma }^{2}I\right)\\ {\hat{\mathbf{y}}}_{t}={D}{\mathbf{x}}_{t}\end{array}$$

This signal is exponentially smoothed with the prior velocity ($\alpha =0.94$) and scaled by a fixed gain *β* (determined via sweeps, as described below) before updating the cursor state. We used a dwell-based target selection with a hold time of 500 ms.

### Matching simulator and participant encoding strength

To compare the SNR between T5 and our simulation, we use an SNR metric that models offline decoder outputs ${\hat{\mathbf{y}}}_{t}$ as a scaled, noisy readout of the true point-at-target vector ${\hat{\mathbf{y}}}_{t}=c{\mathbf{y}}_{t}+\mathbf{b}+\mathbf{\varepsilon}$, where **b** is a constant bias term, *c* is a scalar reflecting the overlap between true and intended command signals and ${\mathbf{\varepsilon }}\sim N\left(0,{{\rm{\sigma }}}^{2}\right)$. We can then define the SNR of a single recording session as $\mathrm{SNR}=\frac{c}{{\rm{\sigma }}}$. This metric is highly correlated with offline *R*^2^ (*r* = 0.73 and *r* = 0.92 for experimental and simulated; Supplementary Fig. 5b, insets), but is insensitive to gain miscalibration on a test block as the *a* term absorbs constant gain differences. We fit this model using offline linear decoder outputs, while avoiding timepoints that were either: (1) shortly after a new trial began (within 200 ms for T5 and within 140 ms for the simulator); or (2) close to the target (<300 pixels for T5 and <0.3 units for the simulator).

We then changed the tuning strength of our simulated channels to match T5 by changing the overall magnitude of the tuning coefficients (the population PD-norm) across channels, ${||}{E}_{x}{||}$, where ${E}_{x}={\left[{e}_{1},\ldots ,{e}_{192}\right]}^{T}$, to align with the SNR values obtained empirically from T5. When plotting the simulated population PD-norm against the associated SNR across simulation runs, we saw a tight correlation (*r* = 0.99). This meant we were able to adjust the simulator’s SNR distribution by simply finetuning the population PD-norm distribution. To find an appropriate PD-norm distribution, we then fit a Gaussian mixture model (*n*_Gaussians_ = 2) to T5’s SNR using expectation maximization. Simply rescaling the value of this distribution then provides the correct PD-norm distribution in the simulator. During simulator use, we randomly sampled from the Gaussian mixture model (GMM), followed by scaling and bias to adjust the output to the corresponding magnitude value:

$$\begin{array}{c}{\rm{SNR}}\sim {\rm{GMM}}\left(\left\{{\mu }_{1},{\mu }_{2}\right\},\left\{{\sigma }_{1},{\sigma }_{2}\right\}\right)\\ {||}{E}_{x/y}{||}\sim a{\rm{SNR}}+b\end{array}$$

### Matching simulator and participant encoding drift

To examine encoding drift across time in our participant recordings, we measured the cosine angle difference $\cos \left({{\rm{\theta }}}_{i,j}\right)$ of the encoding weights for different pairs of sessions *i* and *j* (and separately for *x* and *y* subspaces within these pairs). We then fit exponential decay models that suppose that $\cos \left({\theta }_{i,j}\right)$ decays by some constant multiple across time, that is $\cos \left({{\rm{\theta }}}_{i,i+k}\right)=b{{\rm{\alpha }}}^{k}$, where *b* is a constant and set to 1 in practice; this latter assumption supposes that the within-day encoding is stable. We can then estimate *α* by solving the least-squares problem $\cos \left({{\rm{\theta }}}_{i,i+k}\right)={{\rm{\alpha }}}^{k}$ in log-space, $\log \left(\cos \left({{\rm{\theta }}}_{i,i+k}\right)\right)=k\log \left({\rm{\alpha }}\right)$, which amounts to linear regression. As the logarithm is undefined for negative values, we mask data points where the cosine angle value is less than zero in the optimization. The resulting coefficient is the logarithm of our decay parameter; exponentiating this value yields the decay estimates *α* = 0.913 and 0.925 in Supplementary Fig. 5c.

Next we would like to instantiate the same drift rate in our simulated units. To do so, let us again consider the drift model $${E}^{\prime} \propto$$$$\alpha{E}+\sqrt{1-{\alpha}^{2}}{P}$$. With a few simple assumptions, the shrinkage parameter *α* here aligns with the cosine-angle difference decay parameter described above. To see this, consider the cosine angle difference:

$$\cos \left(\theta \left({E}^{{\prime} },E\right)\right)=\frac{{\alpha{E}}\cdot {E}+\sqrt{1-{\alpha}^{2}}{P}\cdot {E}}{\left|\left|{\alpha{E}}+\sqrt{1-{\alpha}^{2}}{P}\right|\right|{\rm{||}}{E}{\rm{||}}}$$

Now we make two simplifying assumptions: (1) *P* is orthogonal to *E*; and (2) that the magnitude of ${E}^{\prime}$ is roughly equal to *E*. The first assumption is relatively safe for high-dimensional recordings (random vectors are nearly orthogonal in high dimensions) and the latter is equivalent to saying that SNR is roughly equivalent across days. This yields:

$$\cos \left(\theta \left({{E}}^{{\prime} },{E}\right)\right)=\frac{\alpha {{||}{E}{||}}^{2}}{{{||}{E}{||}}^{2}}=\alpha$$

In practice, we use an explicitly orthogonal *P* (as this mimics the anticipated task setting of recalibration in the high-dimensional channel count regimen), but vary the tuning strength of the simulator, which violates the second assumption. However, the approximation still holds well as we obtain an empirical cosine angle decay of *α* = 0.909 when using *α* = 0.910 for our shrinkage parameter.

### Performance sweeps

We simulated two months of data for each recalibration approach by first building an initial linear decoder on day 0. All decoders used 200 s of open-loop data and a fixed smoothing coefficient of 0.94. Optimal gain was determined by sweeping across ten evenly spaced values from 0.1 to 2.5 and measuring closed-loop trial times (400-s blocks).

We then applied different recalibration schemes while slowly modifying the neural population activity. On each new day, we updated the encoding matrix *E* with an orthogonal *P* via ${E}^{\prime} \propto {\alpha{E}}+\sqrt{1-{\alpha}^{2}}{P}$, where *α* = 0.91. We then rescaled the column magnitudes by sampling from the GMM fit to T5’s SNR distribution (see above). Next we applied the previous day’s decoder and obtained 400 s of closed-loop control data. This block was used for recalibration, followed again by an optimal gain sweep, and the resulting decoder was tested once more on a new block to obtain trial time measures. The decoder was then saved and redeployed again the following day after following the above steps once more. In practice, we independently initialized and updated the encoding matrix for each run to maintain statistical independence across simulations. Each method was simulated in 200 such independent runs.

For dataset size sweeps, we reduced the length of the recalibration block while measuring average trial times on a fixed-size evaluation block lasting 400 s.

For the SNR sweep, we modified the norm of the population encoding matrix ${||}{E}{||}$ while running each method across days as before (200 repetitions per method). Trial times were measured at 30 days out.

For the error accumulation analysis in Fig. 4e, we ran subspace stabilization until the angle between decoder and encoding weights (collapsed across *x* and *y* dimensions) reached at least 70°. At this point, we then continued with subspace stabilization or another recalibration method for another five days while keeping track of the angular error. We repeated this analysis across varying training dataset sizes for those five days. We used a fixed population norm here to better isolate the effect of the recalibration strategy for each dataset size. We report relative error, which is simply the new error angle normalized by the existing error when the threshold was first tripped (that is, $\mathrm{relative}\,\mathrm{error}=\frac{\left({\mathrm{error}}_{\mathrm{new}}-{\mathrm{error}}_{\mathrm{old}}\right)}{{\mathrm{error}}_{\mathrm{old}}}$). Hence, a value of −0.3 corresponds to a 30% error reduction.

### Simulator hyperparameter sweeps

We tested three unsupervised recalibration methods in simulation (PRI-T, FA stabilization and RTI), as well as a simple gain recalibration baseline where decoder weights were fixed and only the gain was optimized. For the three unsupervised methods, we separately examined iterative or chained recalibration strategies versus a static approach where the reference decoder is fixed.

For all methods, we simulated 30 days of unsupervised recalibration, with slowly varying neural tuning, as described earlier, and applied various hyperparameter settings in a grid search fashion (Supplementary Fig. 6). Decoders were initialized using 200 s of open-loop activity and recalibrated using 200 s of closed-loop data on each new day.

We tested each hyperparameter set with 30 independent runs. The hyperparameter sets that minimized the median trial time for each method at the 30-day point were selected for subsequent performance comparisons (Extended Data Table 2).

### Closed-loop evaluation

Task logic and visualization were implemented in Python using the PyQtGraph library. At the start of a trial, a fixed-size target was generated randomly on the screen. The user then had to move the cursor over the target and dwell for a consecutive, 500-ms period to select it. Trials lasting more than 10 s were counted as failures. Upon trial completion, audio feedback was provided to the user to indicate whether or not the trial was successful and a new target was generated immediately. Open- and closed-loop blocks were 2 and 4 min long, respectively.

Neural data were binned in 20-ms, non-overlapping bins and fed through a linear regression model *D* trained to predict the cursor-to-target displacement (defined as $\mathbf{g}_{t}-\mathbf{p}_{t}$, where $\mathbf{g}_{t}$ is the current target position and $\mathbf{p}_{t}$ is the cursor position). The raw velocity signal $\mathbf{v}_{t}$ was exponentially smoothed with the running velocity average $\mathbf{c}_{t}$ and scaled with a gain parameter *β* via $\mathbf{c}_{t}=\alpha \mathbf{c}_{t-1}+\left(1-\alpha \right){\rm{\beta }}\mathbf{v}_{t}$, where *α* is the smoothing term and *β* is the gain parameter. Smoothing and gain were manually adjusted during the first session and fixed on subsequent days. We also applied bias correction (as in ref. ^3^) to reduce the impact of mean shifts by subtracting a running estimate of the decoder bias **B**_*t*_ from the raw velocity output. We used an adaptation rate of 0.3 for bias updates.

After a closed-loop block was over, the running bias estimate was saved to the deployed decoder’s file, to provide a better initial bias estimate when used later that same session (or the following session). We obtained confidence intervals for average trial times using bootstrapping with 10,000 iterations.

### Initial day

On day one, we trained an initial linear decoder using T5’s neural activity while he engaged in an open-loop block. This decoder was then used to drive closed-loop control in a subsequent block. We then built two decoders based on this closed-loop block: a standard decoder using the full neural time series; and an FA subspace decoder (*n*_components_ = 6). Both decoders were trained to predict the cursor-to-target displacement signal. The standard decoder was then used as the stale baseline decoder and also as the initial decoder for PRI-T. The subspace decoder, as well as the associated FA model, were used as the reference session for FA stabilization on each new day (this is a fixed stabilization strategy).

### Subsequent sessions

We collected an open-loop block at the beginning of each new session and later measured decoder performance offline (the correlation of outputs with ground-truth displacement vector). We obtained 95% empirical confidence intervals by bootstrap resampling paired prediction and ground-truth timepoints 10,000 times.

To counteract strong session-to-session mean firing rate changes, we collected a 30-s rest block on each day, during which T5 was instructed to relax. We then updated the intercept term of all decoders such that the average decoded velocity vector during the rest block was zero, enforcing that the cursor did not move when the user was not actively doing anything.

With all three approaches (fixed, FA stabilization and PRI-T), we then used the corresponding decoder from the previous session in an initial closed-loop unsupervised recalibration block. We then recalibrated the decoder weights using the block’s data, followed by column-wise rescaling to match the initial day 0 decoder column norms (to keep the overall gain relatively constant) and then tested it in an evaluation closed-loop block. We then repeated this process one or two more times depending on the amount of time available in the session. We interleaved blocks from the three methods in an A/B/C–A/B/C–… fashion to avoid biasing the results from performance differences across time.

Decoders were recalibrated for each of the three methods as follows. For the mean recalibration strategy, we simply ran the closed-loop block with the fixed decoder; bias was automatically corrected for via bias correction. We then initialized bias correction on subsequent blocks using the latest bias correction state from the previous block. This latter strategy was not used with subspace stabilization or PRI-T, as decoder weights were updated after each closed-loop block (and hence the estimated bias differed from the previous block’s estimate).

For FA stabilization, neural data were smoothed offline (causal half-Gaussian filter; *σ* = 40 ms) and reduced to a six-dimensional FA subspace, then realigned to the reference subspace (as in ref. ^6^; *B* = 100; threshold = 0.01). At run time, neural data were pushed through these mappings then decoded by a linear regression fit on day one. This amounted to a reduced-rank regression, $\mathbf{y}_{t}={DQE}\mathbf{x}_{t}$, where ${E}\in {{\mathbb{R}}}^{6\,\times 192}$, ${Q}\in O(6)$ and ${D}\in {{\mathbb{R}}}^{2\,\times \,6}$. The outputs were then smoothed and debiased, as described earlier.

For PRI-T, we infered pseudo-target labels then recalibrated by training a new decoder from scratch with weighted least squares.

When displaying trial times for a given session, we removed the first 60 s from the first block of each decoder. This burn-in period generally contained heavy bias, which was gradually removed by an adaptive bias correction algorithm.

### Offline personal use sessions with T11

We obtained recordings from three separate sessions with participant T11. Similar to T5, T11 has two, 96-channel microelectrode arrays placed in the hand knob area of the dorsal precentral gyrus. During these sessions, T11 was using his iBCI system for personal use, performing various self-prompted tasks such as email typing, video calls and web searches (Fig. 6a,b). T11 used a set of discrete gestures to perform multiple selections. We selected these specific sessions based on several criteria: (1) a sufficiently high SNR such that T11’s control was reasonable throughout the session; (2) inclusion of both personal use and target acquisition task blocks; and (3) long recording times.

These session data were collected as part of an iBCI stability study^16^, which investigated the use of a fixed recurrent neural network (RNN) decoder over several months. For trial days 665 and 702, an updated RNN decoder—adjusted for new data from these days—was used across all blocks (including Fitts task blocks for the test block in this study). For trial day 672, an older RNN decoder (trained on trial day 665—one week previously, with high performance on this new day), was used in place for the test blocks (centre-out task).

### Neural signal preprocessing

Spikes were identified based on threshold crossings at −3.5 multiplied by the root mean square. The resulting neural time series were then binned in non-overlapping, 20-ms bins to generate firing rate estimates. From each session, we selected personal use blocks as training data and held-out target acquisition blocks from each day as test data^16^.

As T11’s signals were noisier than T5’s, we performed a modified post-processing scheme. First, a larger causal Gaussian filter was used (*σ* = 120 ms) to generate population firing rates $\mathbf{x}_{t}\in {{\mathbb{R}}}^{192}$ at each timestep *t*. We also *z* scored this time series using a sliding window (12 s) of past data. For each test block, the running estimate was initialized using the last 12 s of the previous block.

### Self-training comparisons

We estimated cursor-to-target intention vectors using pseudo-labels from PRI-T and RTI. For RTI, all gesture-based selections were considered clicks. We then rescaled the vector norms to equal the decoded speed at each timestep, thereby suppressing time periods for which T11 wasn’t engaged in the task. This latter step greatly increased the performance for the personal use setting, but not in T5’s data. We then trained a linear decoder using preprocessed neural signals across T11’s arrays.

As in the offline T5 analysis, we used decoder output correlations with the point-at-target vector as a performance metric. We split test blocks into 30-s segments to obtain multiple independent measures of performance for each recalibration method (enabling variance quantification and significance testing), yielding *n* = 38, 18 and 19 distinct segments for days 665, 672 and 702, respectively. Statistical significance at each dataset size was evaluated using a Wilcoxon signed-rank test (with the false discovery rate controlled via the Benjamini–Yekutieli method). We removed the first 60 s of each test block from evaluation to reduce the impact of mean shifts and initial participant adjustment to the task.

We used T5’s PRI-T hyperparameters here for T11. For click PRI-T and RTI, we swept hyperparameters while measuring mean performance across 30-s folds on the test data. Sweep settings and results are shown in Extended Data Table 3 (see Supplementary Fig. 8 as well). Selecting RTI and click PRI-T parameters based on held-out task performance gives them the best chance against PRI-T; nevertheless, we still found that PRI-T outperformed RTI in terms of data efficiency while rivalling click PRI-T.

### Reporting summary

Further information on research design is available in the Nature Portfolio Reporting Summary linked to this article.

## Supplementary information

Supplementary Information Supplementary Figs. 1–8.

Reporting Summary

Peer Review File

## Figures and Tables

**Extended Data Table 1:**
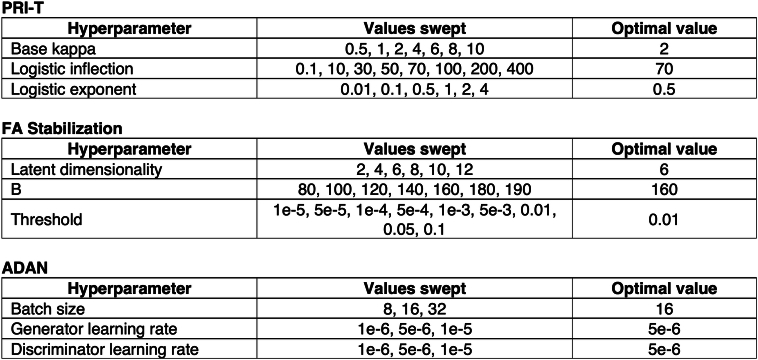
Offline hyperparameter sweeps. Values swept for each approach and corresponding optimal values.

**Extended Data Table 2:**
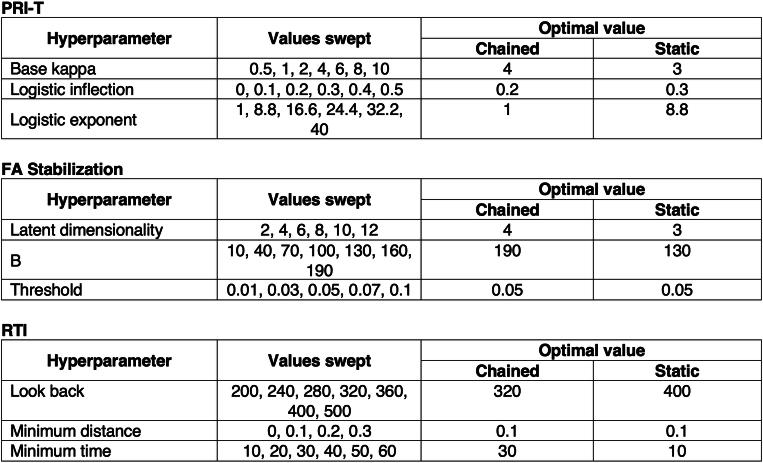
Simulator hyperparameter sweeps. Hyperparameters swept for different approaches in a closed-loop simulation environment. Optimal values minimized the median trial time at the 30-day mark.

**Extended Data Table 3:**
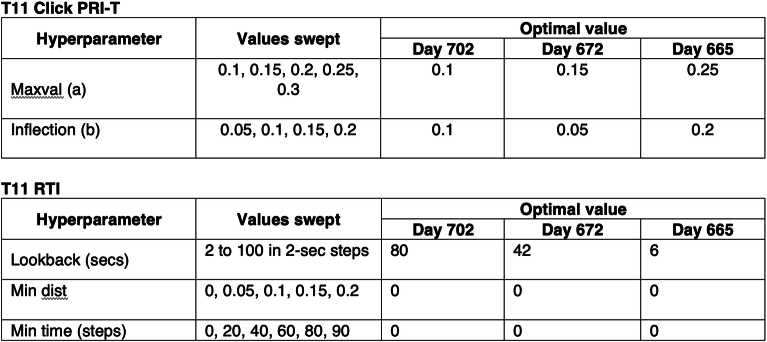
Free response hyperparameter sweeps. Click PRI-T and RTI hyperparameters were optimized within each free response day by sweeping listed values. Optimal values are shown in the rightmost columns.

## Data Availability

The closed-loop datasets supporting the results of this study are available from 10.5061/dryad.1jwstqk6g. Due to privacy considerations, the personal use datasets are not available.

## Code availability

Custom code for this study is available from GitHub at https://github.com/guyhwilson/nonstationarities. A more compact, standalone version of PRI-T is available at https://github.com/guyhwilson/PRI-T.
