# Supplementary material for: Long-term unsupervised recalibration of cursor-based intracortical brain–computer interfaces using a hidden Markov model
Source: Nat Biomed Eng. 2025 Dec 8;10(7):1466–84. doi: 10.1038/s41551-025-01536-z (PMC13375652; doi:10.1038/s41551-025-01536-z)
Supplement: Supplementary file 1 — Supplementary Figs. 1–8. [file 41551_2025_1536_MOESM1_ESM.pdf]

nature biomedical engineering

Supplementary information

https://doi.org/10.1038/s41551-025-01536-z

Long-term unsupervised recalibration of cursor-based intracortical brain–computer interfaces using a hidden Markov model

In the format provided by the authors and unedited

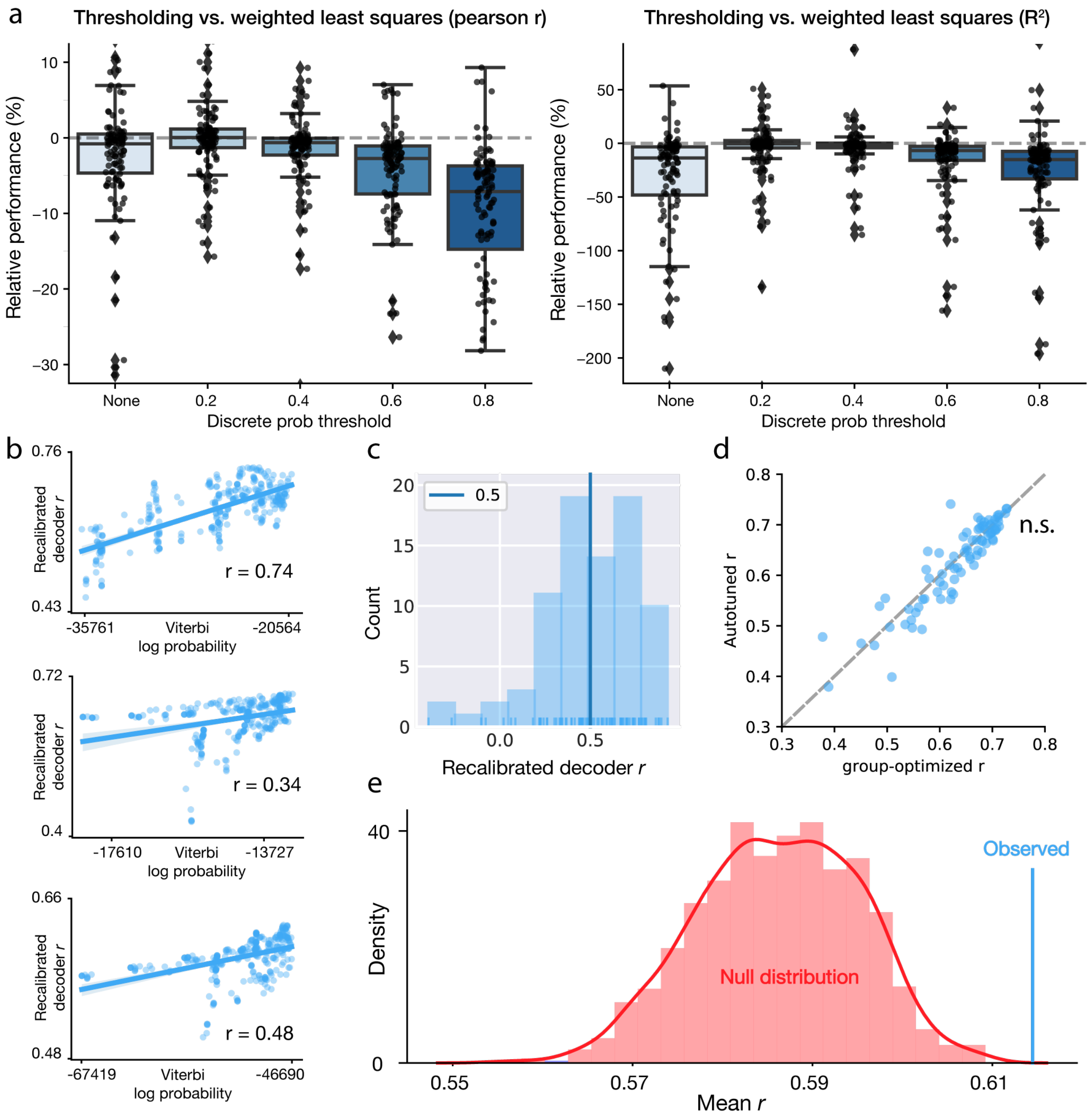

Supplementary Fig. 1 | Supplementary PRI-T analyses. a, PRI-T performance change on a subset of session pairs (N=125) using a discrete cutoff in place of weighted least squares (% relative to weighted strategy). "None" corresponds to case where all timepoints are included and equally weighted. Left: relative change in Pearson *r* (medians: -0.8%, 0.1%, -0.6%, -2.7%, -7.1%). Right: relative change in R2 scores (medians: -13.7%, -0.4%, -1.3%, -6.7%, -15.2%). N=125. Centre line, median; box limits, upper and lower quartiles; whiskers, 1.5x interquartile range; diamond point, outliers. **b,** Example comparisons of Viterbi probabilities for different hyperparameter sets (N=338) against decoder performance when using those values for PRI-T. Solid line, linear regression fit. Shading, bootstrapped 95% confidence intervals (1000 resamples). **c,** Distribution of correlations from **(b)** taken across ~~all~~ a subset of session-pairs (N=81). Most session-pairs have a positive correlation between the two. **d,** Comparison of recalibration strategies across session pairs (N=81): performance using autotuning is slightly worse than a standard grid search (although not statistically significant: $p = 0.2$, two-sided shuffle test, 1000 resamples). **e,** Autotuning improves

performance beyond expected by randomly picking values (p =0.001, two-sided shuffle test, 1000 resamples).

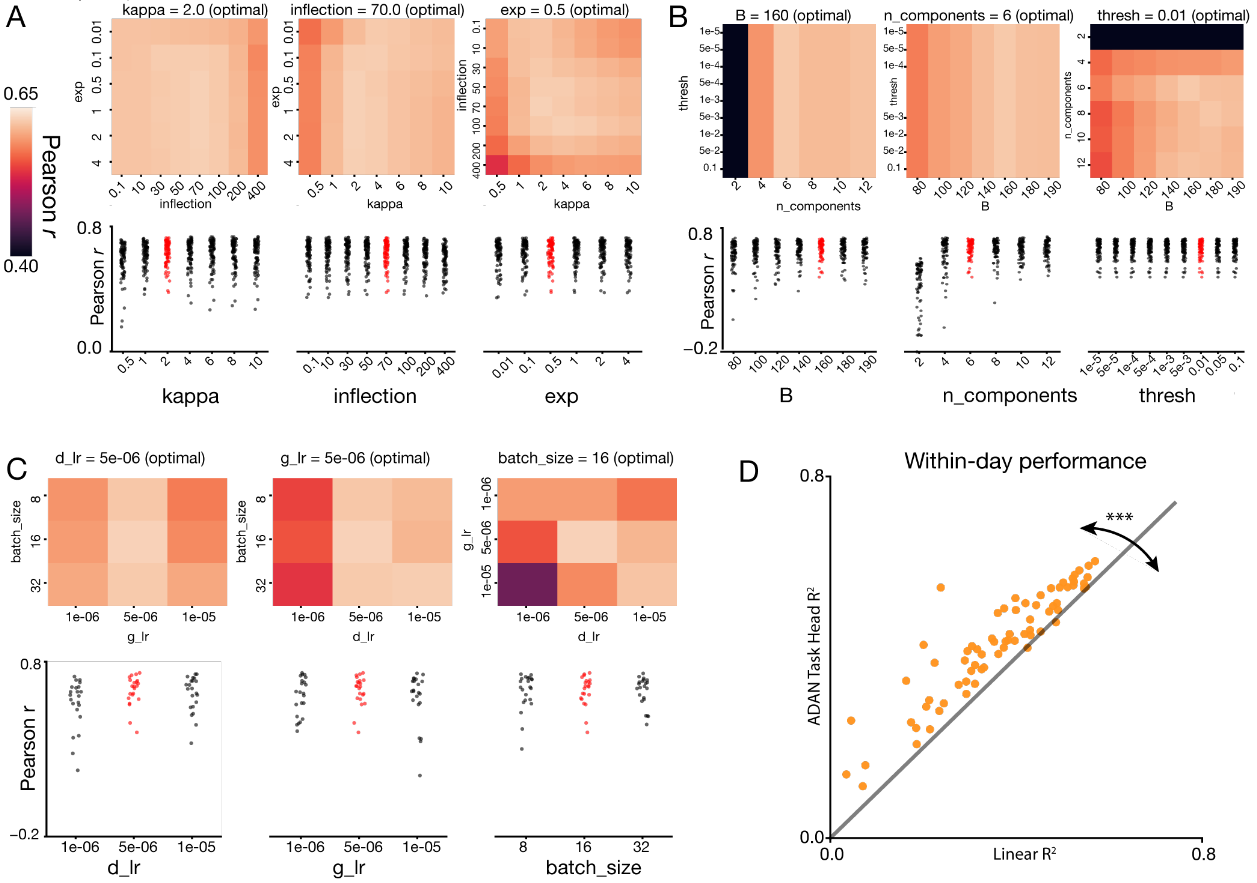

Supplementary Fig. 2 | Offline hyperparameter sweeps. Sweeps are performed across N=81 pairs. **a,** Offline PRI-T hyperparameter (HP) sweeps. Top: 2D heatmaps of median Pearson correlations as a function of HPs (left-out value is set to optimal). Bottom: Correlation values while varying one HP at a time (others are set to their optimal values). Highlighted red scatters indicate optimal values in sweep (maximizing median correlation). **b**, Offline FA stabilizer sweeps. **c**, Offline ADAN hyperparameter sweeps. **d**, ADAN models improved on linear regression by 23% on average within-day (N= 73; $p < 0.001$, two-sided Wilcoxon signed-rank test).

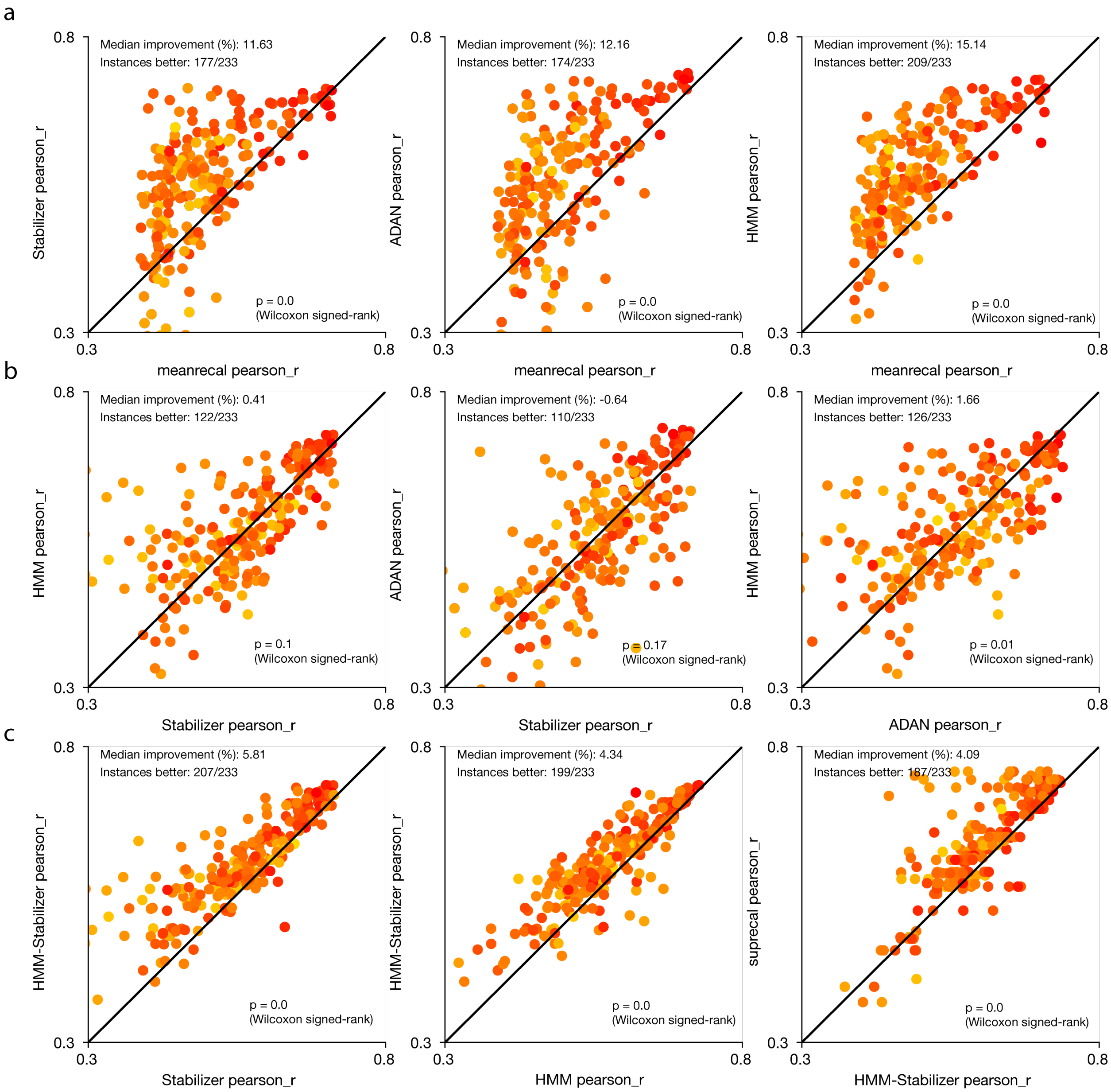

Supplementary Fig. 3 | Pairwise offline results (Pearson correlation). a, Pairwise comparisons plotted for PRI-T, FA stabilizer, ADAN against mean recalibration. PRI-T, stabilizer, and ADAN all generally outperform mean recalibration (> 75% of cases for all three, $p < 0.001$ all, double-sided Wilcoxon signed-rank test). **b,** Comparisons among PRI-T, ADAN, and FA stabilizer. Methods are all roughly equivalent. **c,** Combined approach outperforms versus PRI-T and stabilizer individually (>85% of the time in both cases). Combined decoder is slightly worse than supervised recalibration (supervised outperforms in ~80% of cases with a median 4% improvement).

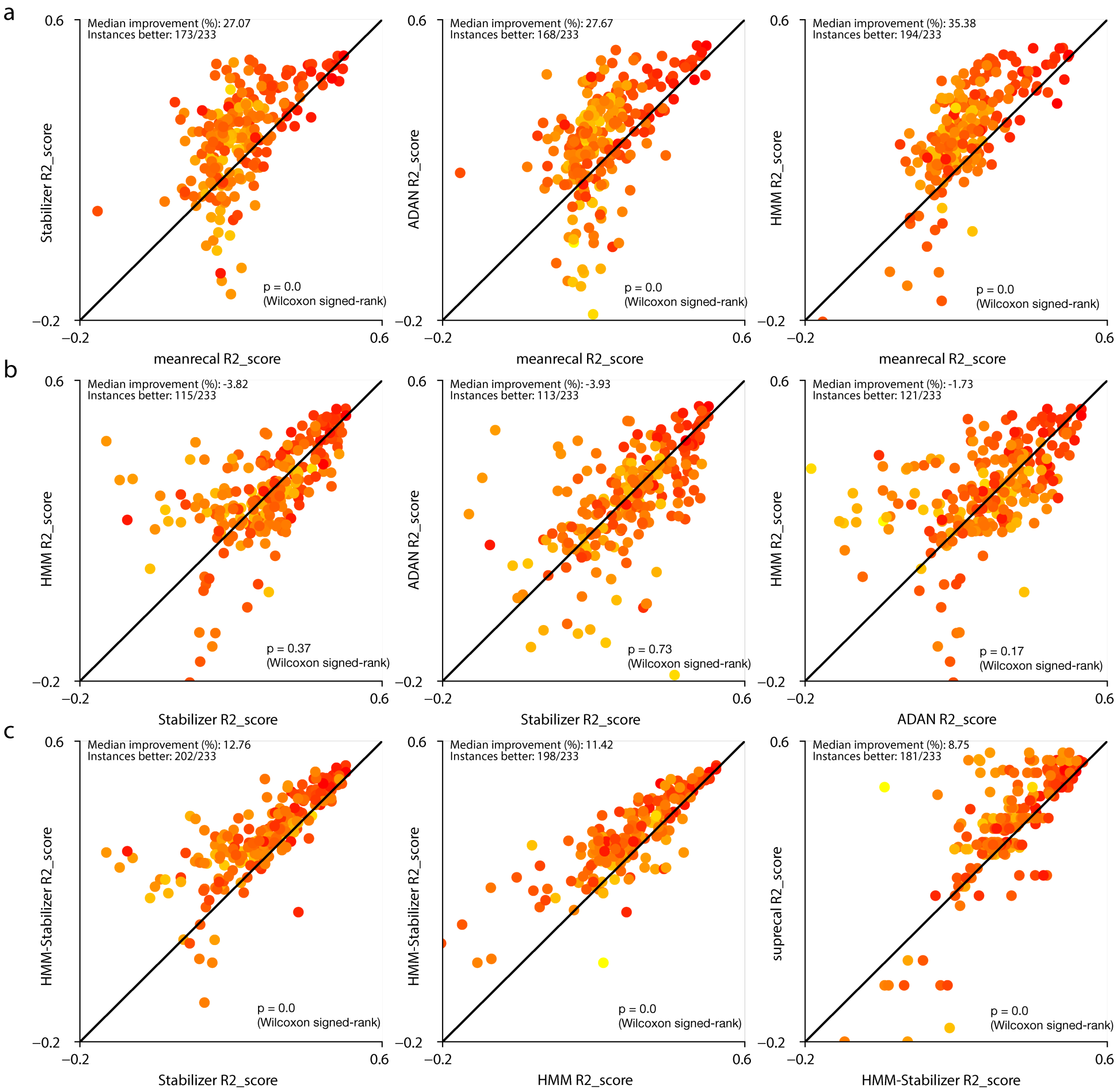

Supplementary Fig. 4 | Pairwise offline results (R2). Same as Supplementary Fig. 3 but with R2 score. **a,** Pairwise comparisons plotted for PRI-T, FA stabilizer, ADAN against mean recalibration. PRI-T, stabilizer, and ADAN all generally outperform mean recalibration (> 72% of cases for all three, $p < 0.001$ double-sided Wilcoxon signed-rank test). **b,** Comparisons among PRI-T, ADAN, and FA stabilizer. Methods are all roughly equivalent. **c,** Combined approach outperforms versus PRI-T and stabilizer individually (>84% of the time in both cases). Combined decoder is worse than supervised recalibration (supervised outperforms in ~77% of cases with a median 9% improvement).

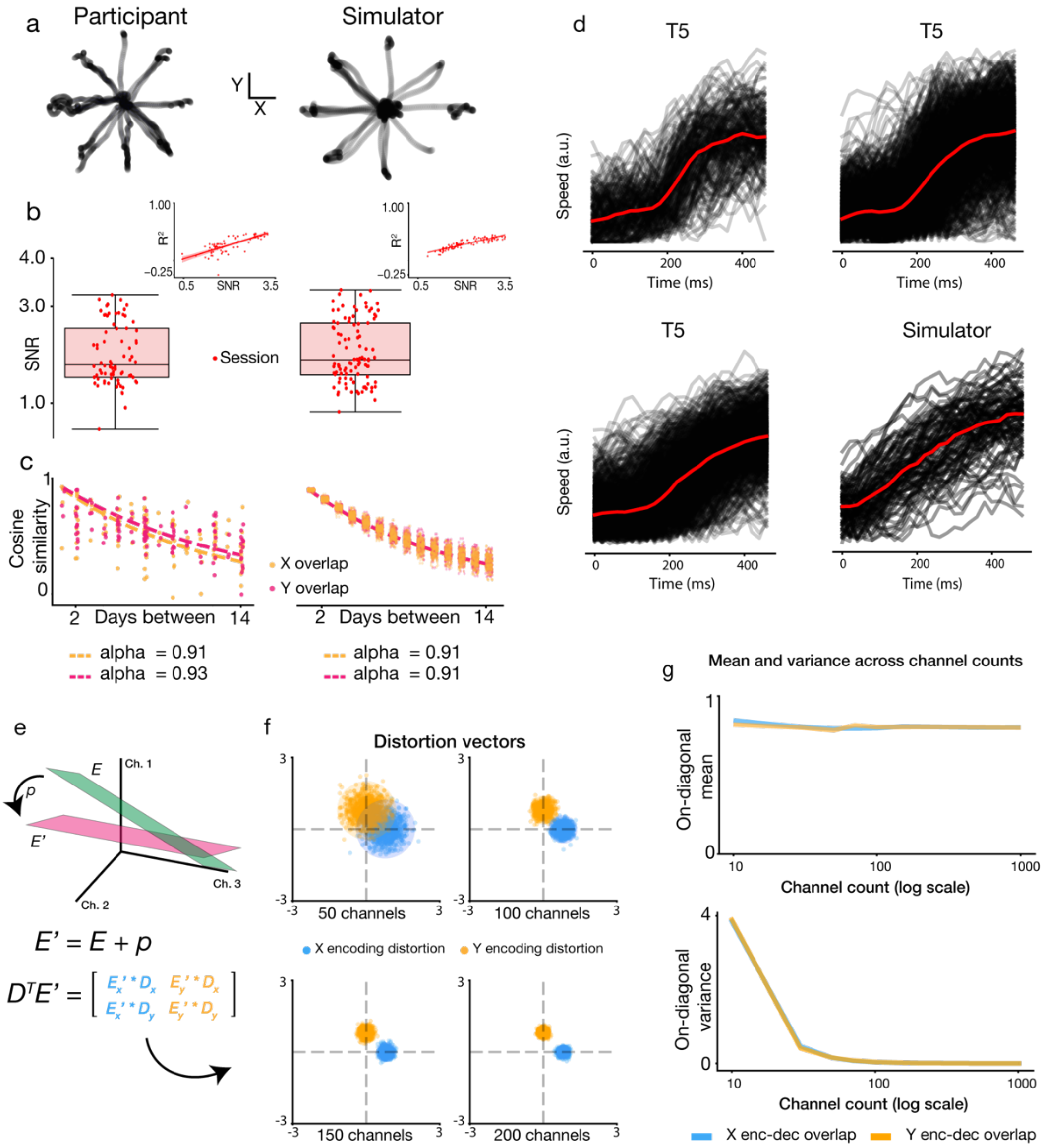

Supplementary Fig. 5 | High channel count systems and simulator. a, Example trajectories during real (left) and simulated (right) closed-loop BCI use. **b,** Distribution of SNR values in participant T5 (left) and simulation (right). T5's SNR distribution (median 1.85, 1.53-2.59 IQR; N=73) closely matches the simulator (median 1.97, 1.53-2.65 IQR). For simple visual comparison, we plot N=100 datapoints drawn from the simulated SNR distribution. Box plot center lines, median; box limits, upper and lower quartiles; whiskers, 1.5x interquartile range. Insets show correlation with offline R^2 during heldout timepoints (shading: bootstrapped 95% confidence intervals). Median R^2s were close between the two settings (0.28 vs. 0.30 for experimental and simulated) and with similar IQR ranges (0.18 - 0.40 experimental, 0.22 - 0.37 simulated). **c,** Cosine angle difference of tuning models across separate sessions in T5 (left) and simulation (right). Dotted lines show exponential decay fits. **d,** Example cursor speeds, aligned to trial starts, during real and simulated closed-loop BCI use. Red lines denote median across trials. **e,** Problem overview: on each new session, the encoding space E is shifted by some random nonstationarity p, yielding the new subspace E'. The effect of this shift on a decoder D can be summarized via a distortion matrix. In an ideal setting with no neural instabilities, this is simply the identity matrix. **f,** Simulated distortion matrices for different channel counts (N=1000 each), plotted as columnwise vectors. The means of these distributions remain constant but their variance diminishes as channel count increases. **g,** left: mean X and Y-subspace overlap across channel counts. Right: variance of overlap across channel counts.

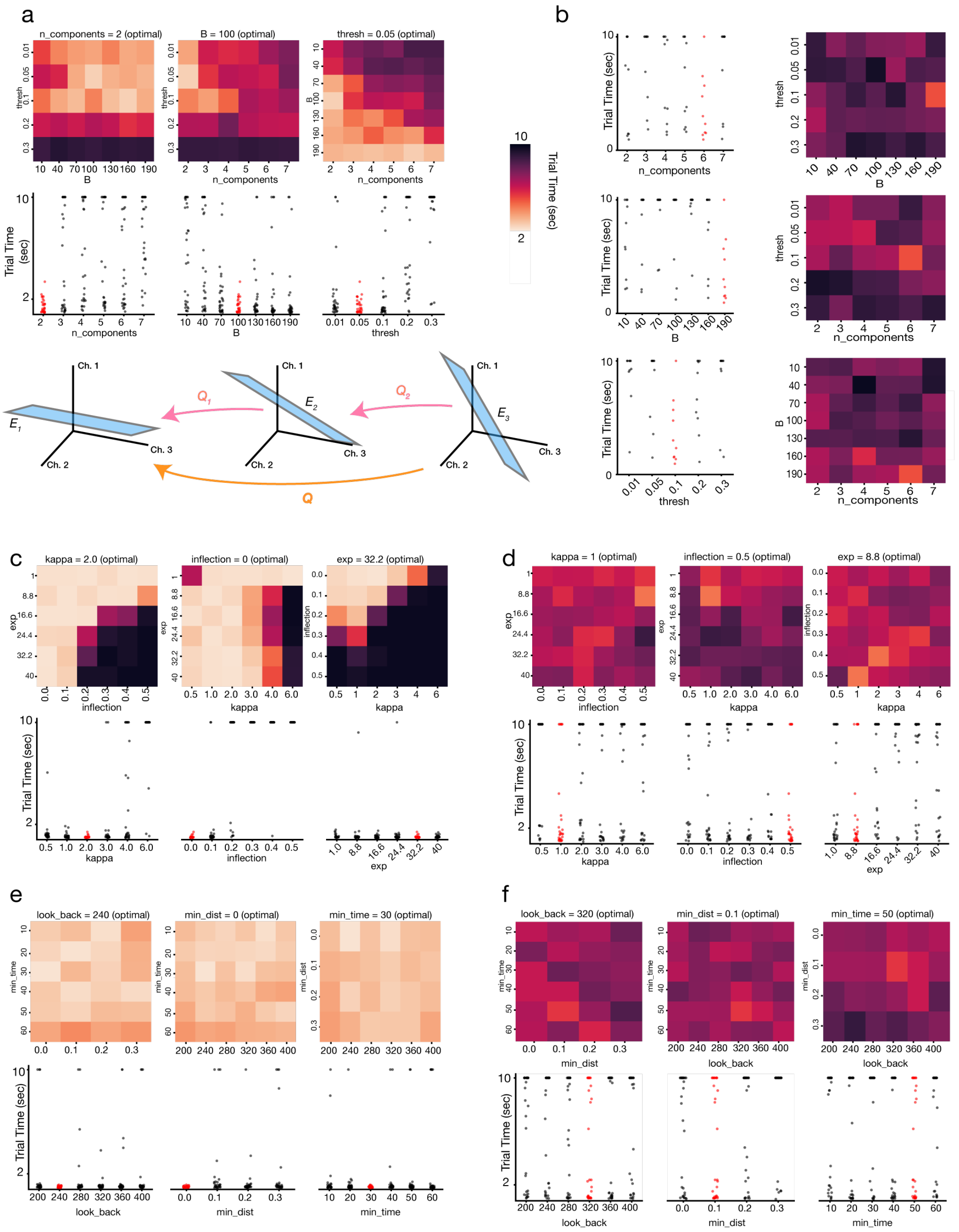

Supplementary Fig. 6 | Simulator hyperparameter sweeps. All hyperparameter (HP) sweeps done using N=30 independent runs for each HP set and evaluated at 30 days with average trial time. Heatmaps show average trial times for each HP set (left-out value is set to optimal). Swarm plots show average trial times while varying a single HP (others are set to optimal). Highlighted red scatters indicate optimal value for this HP (maximizing average trial times). **a**, simulated subspace stabilizer sweeps using a “chaining” strategy (see pink arrows in schematic). **b**, simulated stabilizer sweeps using a standard (“static”, see orange arrow in schematic) approach. **c**, chained PRI-T sweeps. **d**, static PRI-T sweeps. **e**, chained RTI sweeps. f, static RTI sweeps.

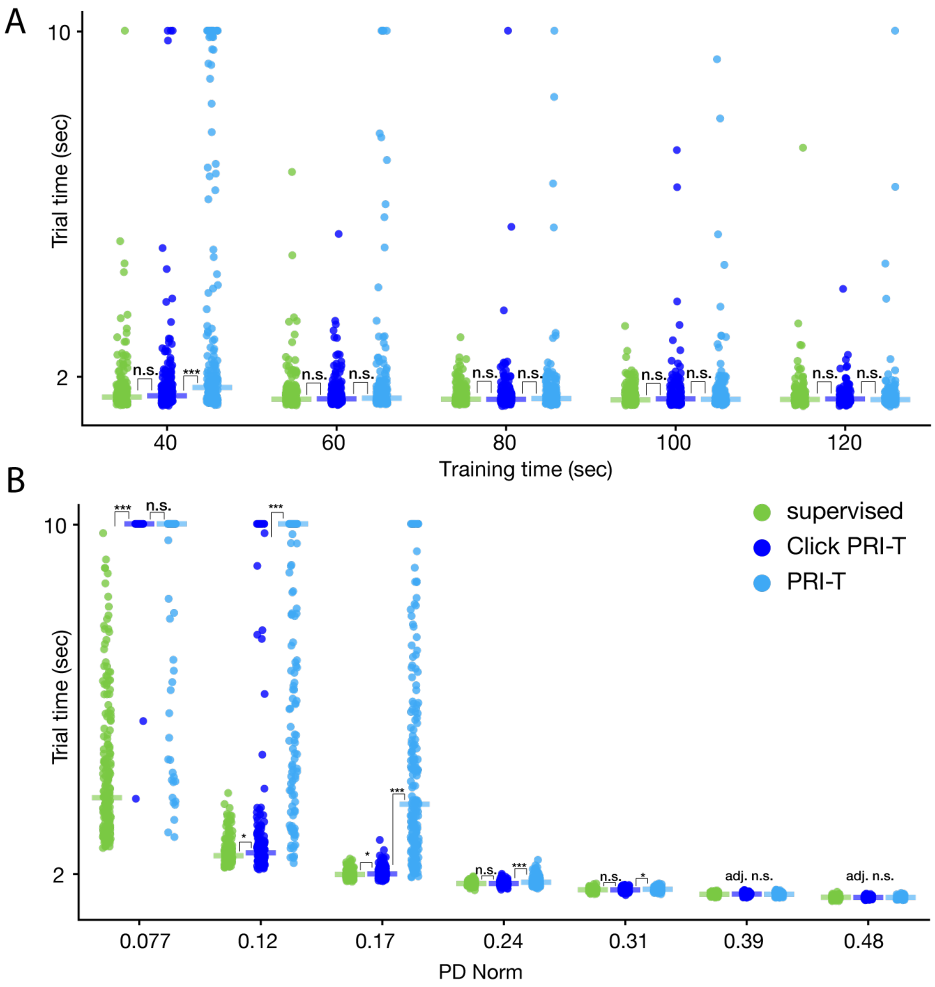

Supplementary Fig. 7 | Adding click to PRI-T. a, Simulated data efficiency comparison of supervised recalibration, PRI-T, and PRI-T with an additional click signal integrated into its observation model. **a,** Average trial times across N=200 independent runs (per method and recalibration block size) are measured at 30 days out. two-sided Wilcoxon rank-sum test: supervised vs click PRI-T at PD-norm=0.12 (p=0.018), supervised vs click PRI-T at PD-norm=0.17 (p=0.043), PRI-T vs click PRI-T at PD-norm=0.31 (p=0.013). All other tests of adjacent approaches were either significant at the 0.001 level (***) or were not significant ("adj. n.s."). **b,** Again simulating performance at 30 days out while varying the population SNR (norm of encoding matrix, see Methods). Recalibration blocks are fixed at 400 seconds long.

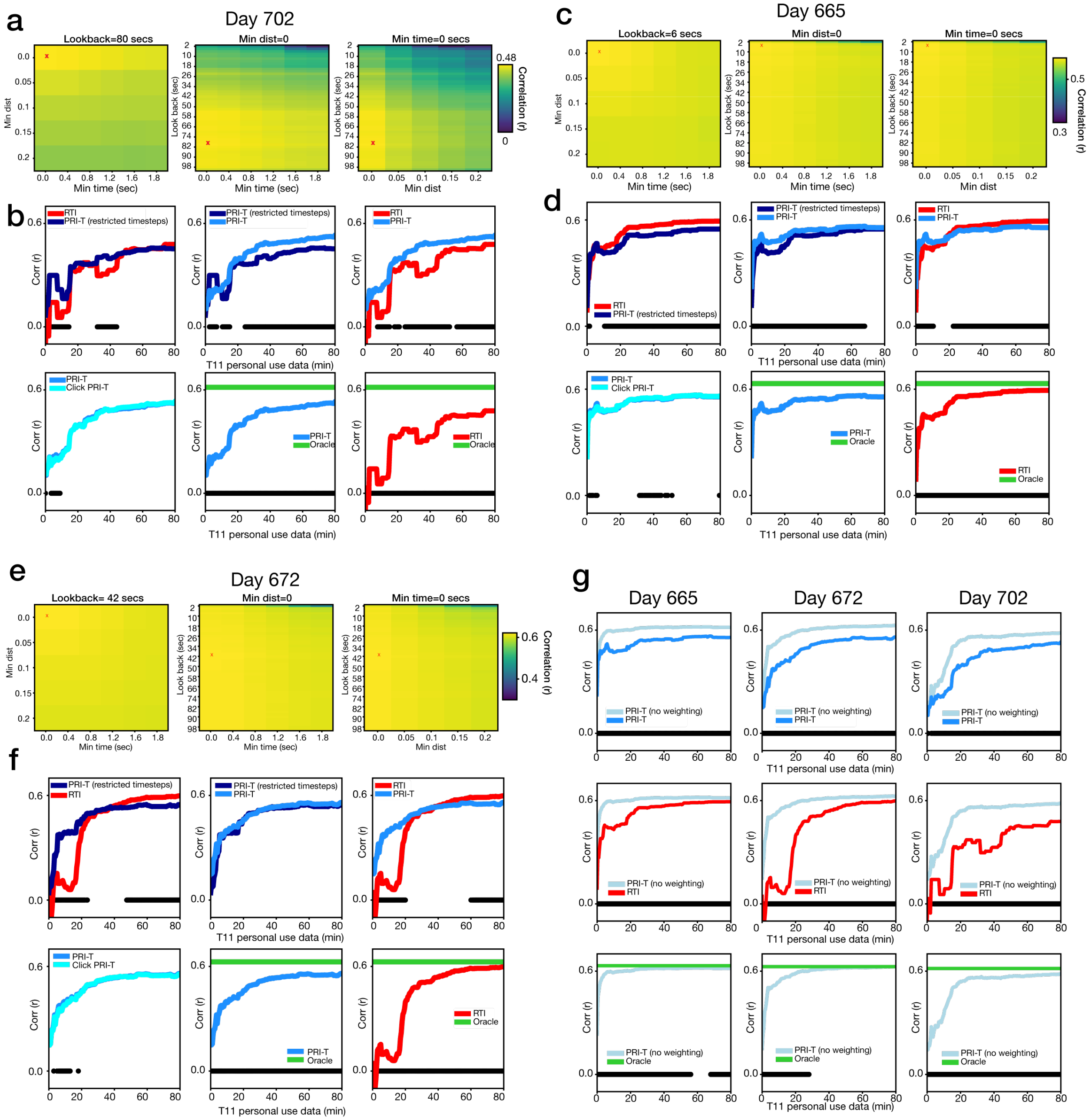

Supplementary Fig. 8 | T11 RTI Sweeps. For a given dataset size, we apply RTI and PRI-T to the user's behavior to infer pseudo-labels for each timestep. To compare pseudo-label quality versus quantity tradeoffs in RTI and PRI-T, we also test a subset of labels from PRI-T ("restricted timestamps") that are restricted to the inference window of RTI, as well as PRI-T without any timestep weighting ("no weighting"). **a,** Grid search results for RTI hyperparameters (HPs) on a personal use session showing average correlation (N=19) between decoder predictions and point-at-target vector on test blocks. Heatmaps show the result of fixing one HP at optimal value (title) while varying the other two. Red crosses denote optimal combination. **b,** Plots of average performance on test block for pairs of methods while varying the amount of training data provided. Black dots denote timepoints with significant differences ($p < 0.05$ with double-sided Wilcoxon signed-rank test). **c-f**, Same as **(a-b)** for two other personal use sessions. N=38, N=18 respectively for day 665 and 672. **g**, comparison of PRI-T without weighting (faded blue) against standard implementation (blue), RTI (red), and an oracle (green) across three personal use sessions.
