# Supplementary material for: Long-term unsupervised recalibration of cursor-based intracortical brain–computer interfaces using a hidden Markov model
Source: Nat Biomed Eng. 2025 Dec 8;10(7):1466–84. doi: 10.1038/s41551-025-01536-z (PMC13375652; doi:10.1038/s41551-025-01536-z)
Supplement: Supplementary file 2 — Reporting Summary [file 41551_2025_1536_MOESM2_ESM.pdf]

nature portfolio

Corresponding author(s): Guy Wilson

Last updated by author(s): March 30, 2025

Reporting Summary

Nature Portfolio wishes to improve the reproducibility of the work that we publish. This form provides structure for consistency and transparency in reporting. For further information on Nature Portfolio policies, see our Editorial Policies and the Editorial Policy Checklist.

Statistics

For all statistical analyses, confirm that the following items are present in the figure legend, table legend, main text, or Methods section.

n/a	Confirmed	
☐	☒	The exact sample size (*n*) for each experimental group/condition, given as a discrete number and unit of measurement
☐	☒	A statement on whether measurements were taken from distinct samples or whether the same sample was measured repeatedly
☐	☒	The statistical test(s) used AND whether they are one- or two-sided<br>*Only common tests should be described solely by name; describe more complex techniques in the Methods section.*
☒	☐	A description of all covariates tested
☐	☒	A description of any assumptions or corrections, such as tests of normality and adjustment for multiple comparisons
☐	☒	A full description of the statistical parameters including central tendency (e.g. means) or other basic estimates (e.g. regression coefficient) AND variation (e.g. standard deviation) or associated estimates of uncertainty (e.g. confidence intervals)
☐	☒	For null hypothesis testing, the test statistic (e.g. *F*, *t*, *r*) with confidence intervals, effect sizes, degrees of freedom and *P* value noted<br>*Give P values as exact values whenever suitable.*
☒	☐	For Bayesian analysis, information on the choice of priors and Markov chain Monte Carlo settings
☒	☐	For hierarchical and complex designs, identification of the appropriate level for tests and full reporting of outcomes
☐	☒	Estimates of effect sizes (e.g. Cohen's *d*, Pearson's *r*), indicating how they were calculated

Our web collection on statistics for biologists contains articles on many of the points above.

Software and code

Policy information about availability of computer code

Data collection	Software for running experiments and recording data were implemented in custom MATLAB 2019b and Python 3.7.11 code. PyQTGraph 0.11.0 was used for real-time visualization.
Data analysis	Custom code for this study is available on GitHub at https://github.com/guyhwilson/nonstationarities. A more compact, standalone version of PRI-T is available at: https://github.com/guyhwilson/PRI-T.

For manuscripts utilizing custom algorithms or software that are central to the research but not yet described in published literature, software must be made available to editors and reviewers. We strongly encourage code deposition in a community repository (e.g. GitHub). See the Nature Portfolio guidelines for submitting code & software for further information.

Data

Policy information about availability of data

All manuscripts must include a data availability statement. This statement should provide the following information, where applicable:

- Accession codes, unique identifiers, or web links for publicly available datasets
- A description of any restrictions on data availability
- For clinical datasets or third party data, please ensure that the statement adheres to our policy

The closed-loop datasets supporting the results of the study are available at https://doi.org/10.5061/dryad.1jwstqk6g. Due to privacy considerations, the personal use datasets are not available.

Research involving human participants, their data, or biological material

Policy information about studies with human participants or human data. See also policy information about sex, gender (identity/presentation), and sexual orientation and race, ethnicity and racism.

Reporting on sex and gender	Sex and gender-specific analyses are not relevant for this study, which includes data from two male participants.
Reporting on race, ethnicity, or other socially relevant groupings	Race and ethnicity-specific analyses are not relevant for this study, which includes data from two Caucasian participants.
Population characteristics	This study includes data from two participants (identified as T5 and T11) who gave informed consent and were enrolled in the BrainGate2 Neural Interface System clinical trial (ClinicalTrials.gov Identifier: NCT00912041, registered June 3, 2009) - but note that this study does not report clinical trial results. T5 is a right-handed man, 69 years old at the time of data collection, with a C4 AIS C spinal cord injury that occurred approximately 11 years prior to study enrollment. T11 is a 36-year-old right-handed male at the time of data collection, with a C4 AIS-B spinal cord injury (SCI) that occurred approximately 11 years prior to study enrollment.
Recruitment	Participants T5 and T11 were enrolled in the BrainGate2 pilot clinical trial prior to the design and execution of this study, after meeting inclusion criteria based in part on disease characteristics. Inclusion and exclusion criteria are available online (ClinicalTrials.gov). Participants were not financially compensated for participation in the study.
Ethics oversight	The BrainGate2 Neural Interface System clinical trial was approved under an Investigational Device Exemption (IDE) by the US Food and Drug Administration (Investigational Device Exemption #G090003). Permission was also granted by the Institutional Review Boards of Stanford University (protocol #20804). All research was performed in accordance with relevant guidelines/ regulations.

Note that full information on the approval of the study protocol must also be provided in the manuscript.

Field-specific reporting

Please select the one below that is the best fit for your research. If you are not sure, read the appropriate sections before making your selection.

☒ Life sciences ☐ Behavioural & social sciences ☐ Ecological, evolutionary & environmental sciences

For a reference copy of the document with all sections, see nature.com/documents/nr-reporting-summary-flat.pdf

Life sciences study design

All studies must disclose on these points even when the disclosure is negative.

Sample size	No sample size calculation was done as results are from two participants. The opportunity to record intracortical signals from human participants is extremely rare, hence here we used available data.
Data exclusions	One session with electrical noise was excluded from some of the offline analyses. The first 60 seconds from the starting block on each new day of on line comparisons with the participant was cut out to remove noise from heavy bias.
Replication	Results were replicated across multiple days of recordings in one participant (239 pairs from 73 sessions for offline results, 21 blocks across 6 different sessions for closed-loop results), in a closed-loop simulation analysis (200 separate simulations for main analysis), and in a separate set of data from an additional participant (3 separate sessions).
Randomization	Randomization into groups is not relevant for this two-participant study.
Blinding	Blinding was not possible as custom codes required tracking of decoder versions for online control A/B testing. During online recalibration comparisons, the participant was not told which decoder was being used in each new block.

Reporting for specific materials, systems and methods

We require information from authors about some types of materials, experimental systems and methods used in many studies. Here, indicate whether each material, system or method listed is relevant to your study. If you are not sure if a list item applies to your research, read the appropriate section before selecting a response.

Materials & experimental systems

n/a	Involved in the study
☒	☐ Antibodies
☒	☐ Eukaryotic cell lines
☒	☐ Palaeontology and archaeology
☒	☐ Animals and other organisms
☒	☐ Clinical data
☒	☐ Dual use research of concern
☒	☐ Plants

Methods

n/a	Involved in the study
☒	☐ ChIP-seq
☒	☐ Flow cytometry
☒	☐ MRI-based neuroimaging

Plants

Seed stocks

Report on the source of all seed stocks or other plant material used. If applicable, state the seed stock centre and catalogue number. If plant specimens were collected from the field, describe the collection location, date and sampling procedures.

Novel plant genotypes

Describe the methods by which all novel plant genotypes were produced. This includes those generated by transgenic approaches, gene editing, chemical/radiation-based mutagenesis and hybridization. For transgenic lines, describe the transformation method, the number of independent lines analyzed and the generation upon which experiments were performed. For gene-edited lines, describe the editor used, the endogenous sequence targeted for editing, the targeting guide RNA sequence (if applicable) and how the editor was applied.

Authentication

Describe any authentication procedures for each seed stock used or novel genotype generated. Describe any experiments used to assess the effect of a mutation and, where applicable, how potential secondary effects (e.g. second site T-DNA insertions, mosiacism, off-target gene editing) were examined.
