# Supplementary material for: Long-term unsupervised recalibration of cursor-based intracortical brain–computer interfaces using a hidden Markov model
Source: Nat Biomed Eng. 2025 Dec 8;10(7):1466–84. doi: 10.1038/s41551-025-01536-z (PMC13375652; doi:10.1038/s41551-025-01536-z)
Supplement: Supplementary file 3 — Peer Review File [file 41551_2025_1536_MOESM3_ESM.pdf]

nature portfolio

Peer Review File

Long-term unsupervised recalibration of cursor intracortical brain computer interfaces using a Hidden Markov model

Corresponding Author: Dr Guy Wilson

Version 0:

Decision Letter:

Dear Mr Wilson,

Thank you again for submitting to *Nature Biomedical Engineering* your manuscript, "Long-term unsupervised recalibration of cursor BCIs" and for your patience in waiting for the reviewer reports and our decision (we had initial difficulties in securing experts who were available and willing to review the work, and one reviewer required more time to review due to unforseen circumstances). The manuscript has been seen by 2 experts, whose reports you will find at the end of this message, (yet despite our chasing efforts one reviewer has yet to provide a report; if the reviewer delivers it, we will also share it with you). You will see that the reviewers appreciate the work, and that they raise a number of technical criticisms that we hope you will be able to address. In particular, we would expect that a revised version of the manuscript provides:

*Clarification that self-training methods outperform distribution alignment in closed-loop BCI, as suggested by both Reviewers.

*Expansion on the comparison between self-training and distribution alignment methods using closed-loop simulations, as suggested by both Reviewers (with compelling arguments and/or additional evidence).

*Clarification of the advantages and disadvantages of distribution alignment methods, as suggested by both Reviewers.

*A discussion of the limitations of the closed-loop experiment results, especially with regard to the structured BCI task, as suggested by both Reviewers.

*Clarification of the interpretation of the results and hypotheses regarding neural signal instability, subspace similarity and differences in variability shown in Fig. 3c, as suggested by both Reviewers.

*Clarification of the confidence estimates for pseudotargets and their interpretation, as suggested by both Reviewers.

*Inclusion of statistical comparisons between recalibration methods in the offline comparisons, as suggested by Reviewer #1.

*Inclusion of non-chained RTI and PRI-T in the comparisons between recalibration methods, as suggested by Reviewer #2.

When you are ready to resubmit your manuscript, please upload the revised files, a point-by-point rebuttal to the comments from all reviewers, the reporting summary, and a cover letter that explains the main improvements included in the revision and responds to any points highlighted in this decision.

Please follow the following recommendations:

* Clearly highlight any amendments to the text and figures to help the reviewers and editors find and understand the changes (yet keep in mind that excessive marking can hinder readability).

* If you and your co-authors disagree with a criticism, provide the arguments to the reviewer (optionally, indicate the relevant points in the cover letter).

* If a criticism or suggestion is not addressed, please indicate so in the rebuttal to the reviewer comments and explain the reason(s).

* Consider including responses to any criticisms raised by more than one reviewer at the beginning of the rebuttal, in a section addressed to all reviewers.

* The rebuttal should include the reviewer comments in point-by-point format (please note that we provide all reviewers will the reports as they appear at the end of this message).

* Provide the rebuttal to the reviewer comments and the cover letter as separate files.

We hope that you will be able to resubmit the manuscript within 20 weeks from the receipt of this message. If this is the case, you will be protected against potential scooping. Otherwise, we will be happy to consider a revised manuscript as long as the significance of the work is not compromised by work published elsewhere or accepted for publication at *Nature Biomedical Engineering*.

We hope that you will find the referee reports helpful when revising the work, which we look forward to receive. Please do not hesitate to contact me should you have any questions.

Best wishes,

Michelle

__
Dr Michelle Korda
Senior Editor, *Nature Biomedical Engineering*

____
Reviewer #1 (Report for the authors (Required)):

This study reports an important result that stable BCI performance can be obtained without explicit recalibration for 28 days. This conclusion is supported through online brain-computer interface performance using a new method of automatic recalibration (PRI-T) that leverages models of kinematic task structure to infer target locations. They also compare to other state-of-the-art recalibration approaches both online and offline. The introduction does a nice job of describing the challenges with achieving stable BCI control and the strengths/limitations of various approaches to overcome this challenge. The results are nicely organized with the key conclusions supported in the main figures and details regarding day-by-day comparisons or model optimization are included in the supplemental materials. The findings reported here address an important challenge in clinical BCI and the results are significant because they demonstrate the potential for very long term stable performance with clinically-relevant amounts of data. I commend the authors on their thorough and rigorous study. Below I include specific comments that I think would help strengthen this manuscript. The primarily limitation that should be discussed further is that the participant was always performing a structured BCI task where they were highly engaged in a very active task. It remains to be demonstrated that this method would generalize to self-selected and self-paced general BCI use (e.g. typing or browsing the web).

1.It is appropriate to discuss some limitations of the closed-loop experiment results (Figure 5) and/or interpretation in greater detail.
a.First, the participant was always performing the same type of structured task (random target presentation and selection). For this type of task, the target is known and explicit recalibration could be performed. For actual BCI use in a home environment, the user would likely perform less structured tasks that could be more variable (e.g. on screen typing vs. web browsing) that may have different kinematic distributions or requirements. Future work will need to evaluate the extent to which this approach is generalizable to less structured or prescribed BCI use.
b.There is a significant gap between Day 14 and Day 28 and the authors comment that neural activity on Day 28 looks quite similar to the reference day so I am not very convinced that the results can generalize to any 28 day period, though this does not change or weaken the conclusions that are stated. Rather, I think that there is an open question about whether this similarity is due to the random walk nature of neural signal instability as proposed, or whether it reflects a return to a particular neural state (e.g. attention level). It may be valuable to verify that the subspace similarity was in fact more similar on this day than on Days 12 and 14. The simulation results assume a continuously increasing amount of instability, which may not be reflective of real world conditions. This is evidenced in the pairwise tests in Figure 3a as well where there is not a continuous decrease in performance for greater separation between trials. The results clearly show that PRI-T performs better than other methods for ever increasing signal instability, which certainly occurs on average over time. I would appreciate if the authors could mention that continuously increasing instability may be a simplification of real world conditions.
c.I understand and agree with the authors rationale for using an interleaving block-design for the online decoder comparison. However, this approach may have limited learning or consolidation of a consistent latent neural structure that other approaches may benefit from (such as FA alignment). This limitation should be noted.
d.For the closed loop experiments, was FA implemented in an iterative manner or always referenced to Day 0? I presume that it was iterative to mimic realistic BCI conditions, but I could not glean this information from the text.

2.Figure 1e includes many sessions where the R2 values is very low, which likely implies nearly unusable BCI decoding. The inclusion of these sessions also obscures the main results described in the text reporting median bias values <1.

3.The authors mention that the lack of confidence estimates for pseudotargets is a limitation of RTI. There is only one example figure (Figure 2d) of confidence estimates for PRI-T but it is a bit difficult to interpret what is being shown. The different colored traces are not defined. The confidence estimates seem to rapidly move from 0 to 1 for these structured tasks. Would this be the case for an unstructured task?

4.This statement seems to be inaccurate: “Both latent space methods tended to have much larger IQRs ([0.83 - 0.96] for stabilizer; [0.89 - 0.98] for ADAN) compared to PRI-T ([0.85 - 0.96]).” The confidence interval for PRI-T falls in between the two latent space methods.

5.I was unable to come up with an explanation for the differing amount of variability shown in Figure 3c. The authors do mention this in the text, but I am curious if there is a hypothesis as to why this was observed.

6.It was unclear how many “targets” (e.g. the n x n spatial grid) were considered in the PRI-T model. Would the number of targets inferred from the various structured BCI tasks be similar to the number of targets that would be appropriate for self-selected tasks during BCI-controlled computer use?

7.The results presented for the click dimension were quite limited. It was unclear how click targets were defined particularly given the combination of hover- and click-based control. Based on the methods, I interpret that the click probability was high when the cursor was near the target (which was also estimated probalistically), but again I wonder how well this would generalize to less structured tasks. The paper would benefit from a more detailed description of how the click targets were defined and showing data regarding the confidence estimates, predicted click timing, and actual click timing.

8.Were the subjects informed that different decoders were being evaluated? Did they report any subjective performance differences between the recalibration methods?

9.I appreciate that the authors were transparent regarding their process for hyperparameter selection as this will obviously impact how well each method performs.

10.There were a number of instances where IQRs were reported without units.

11.Minor: The first paragraph of the methods refers to “participants” but only 1 participant is included in this paper.

Reviewer #2 (Report for the authors (Required)):

This paper shows a new approach for unsupervised recalibration of BCIs based on self-training, without the need for supervised recalibration sessions which require users to interrupt their free use of the BCI (free-use session). As a key strength, the study compares methods in actual closed-loop iBCI experiments for one full month, which is commendable. Unlike this study that focuses on a self-training approach and closed-loop iBCI testing, recent work for unsupervised BCI recalibration have instead focused on distribution matching/alignment approaches with largely offline testing.

The authors develop a new self-training method called PRI-T, which provides an explicit probabilistic HMM model for target inference unlike the prior self-training method of RTI. The HMM model relates targets to cursor kinematics based on a first order Markov chain and uses the forward-backward and Viterbi algorithms to estimate the targets from cursor observations along with the probabilities of the targets during free-use sessions. These target estimates can then be used to recalibrate decoders for subsequent free-use sessions. The method is evaluated in offline analyses of human iBCI data, closed-loop simulations, and critically on closed-loop human iBCI data. Most interestingly, the authors show that self-training improves distribution alignment in closed-loop iBCI experiments over one month of usage. I believe this is the first demonstration of this important result.

The topic of addressing nonstationarity in neural data in iBCI is important. I believe the authors’ result that self-training methods outperform distribution matching/alignment techniques (such as FA stabilization, ADAN, NoMAD) is a very valuable contribution to the field. This is because distribution alignment methods have shown no or minimal validation in actual closed-loop iBCI experiments as the authors correctly note; instead, alignment methods have largely focused on offline testing, which can be quite different from real-world iBCI use by a human. Here, the authors compare on a full month of closed-loop iBCI use, which is commendable. Overall, convincingly demonstrating that self-training (rather than distribution alignment) is the way to go for practical human iBCIs will be a very important contribution and save the field a lot of time by showing the direction to focus efforts on. For that reason, I believe the authors should focus their manuscript on this finding and expand their analyses on it (for example as I suggest below) to make the manuscript even stronger in this respect. The specific self-training method here (PRI-T) is also interesting in that it provides a mathematical formalization of self-training ideas; but to me the general finding that self-training far outperforms distribution alignment is more significant and should be expanded with further analyses. With the below revisions, I believe the paper will be a very valuable contribution.

Major Comments

1.I find the result that self-training far outperforms the distribution alignment/matching methods (both FA stabilization and ADAN) the most important contribution of this study. Indeed, it seems that distribution alignment performs poorly in closed-loop iBCI, almost similar to a fixed decoder. It is thus critical to strengthen this finding as I discussed above. I think the authors have an opportunity to show the reasons why self-training is advantageous for iBCIs with their closed-loop simulation setup.

The authors hypothesize the reason for FA performing poorly in closed-loop iBCI is that "the alignment step may fail if the underlying task manifold is too symmetrical to enforce a unique identification". Also, they show ADAN underperforms and overfits, leading to more variability compared with FA. Further, they state a hypothesis of noise-dependence, saying "One exception is FA stabilization, which was evaluated over a 5 day period in NHPs, and may be optimal for a specific noise regime experienced in shorter timeframes". I think Degenhart et al hypothesize that FA's advantage over supervised methods holds for periods of low user engagement, which may not happen during free-use of a human user unlike an NHP. The closed-loop simulations here provide a good setup to test such hypotheses by simulating different scenarios for the noise, drift, etc. Similarly, ADAN can be trained with more or less data to confirm the above hypotheses about overfitting. Also, hypotheses about other recent distribution alignment methods can be posed and/or tested in closed-loop simulations. I think this is the most important aspect the revision should focus on: expanding on the downsides/comparisons of distribution alignment approaches in general (for iBCIs), which are largely not tested in closed-loop iBCI but rather on offline data.

2.Another contribution here is the development of the new PRI-T method for self-training. Indeed, there are novel differences in methodology in PRI-T vs. the prior RTI method for self-training. Given that at a high-level, both PRI-T and RTI estimate targets from cursor data for recalibration purposes, extending the comparisons can further highlight the advantages of PRI-T. Currently, comparisons to RTI are done in the simulations. How do the methods compare in the offline iBCI data? I appreciate that the closed-loop experiments have already concluded and cannot be repeated but it is also helpful to put a discussion on what the expected performance differences could be.

3.In the simulations, the main advantage of PRI-T vs RTI is noted as requiring less data (about ~2 min less, Fig. 4f). Given that this data seems to be free-use data, the authors should clarify how much of a real-world advantage this provides over RTI and in what situations. Are there situations where free-use data is limited? The authors should highlight such situations. Again, that is why to me item 1 above – showing that self-training in general, regardless of specific method, is better than distribution alignment for iBCIs– is more significant of a contribution and should be emphasized, focused on, and further explored.

4.As the authors discuss, the use of simulations allow for more direct comparisons between methods as well as enable explorations of when one method outperforms the other. Additional simulations that can highlight how PRI-T can utilize the HMM formalization to yield meaningful and practical improvements could help strengthen the manuscript.

5.Comparisons between recalibration methods in closed-loop simulations (Figure 4) currently show both "chained" and non-chained forms of the Stabilizer method where the non-chained appears to use the decoder from day 0 and recalibrate with data from day N while the chained compounds recalibrations over each consecutive day. However, it appears that only the equivalent "chained" forms of RTI and PRI-T methods are used here (as indicated by line 816 where decoders are recalibrated from the immediate prior day rather than only from day 0). While showing the non-chained FA Stabilizer method gives insight into how the method may fail over long time scales, there is then a question of whether the other two self-training methods (RTI, PRI-T) would also fail if non-chained. Analyses with non-chained RTI and PRI-T should be included and discussed.

6.In Figure 3a, there seems to be a persistent block structure in the heatmaps (specifically one block around ~500 to ~1200 days, and one block from ~1200 to the last day). One could interpret this as the recalibration methods improving decoding performance within each block but suddenly and abruptly failing when moving to the other block (e.g., around day 1200). Why is that? Could this be due to some unknown confounding variable that is shared within but not between blocks? This adds difficulty in assessing whether recalibration methods fail due to the variable time or due to some other variable that has not been mentioned (e.g., what is happening right around the end of one block?). Additional information and some discussion on why this pattern is there would be helpful.

Other Comments

1.In the dataset size sweeps in the simulations as shown in Figure 4f, PRI-T is shown to outperform RTI with less data. However, inherently RTI uses less data to train since it discards timesteps for various conditions like being outside the lookback window. It would be interesting to see how PRI-T performs with respect to a modified RTI that uses all timesteps (in other words a varying look back window that extends from prior target to current target selection). In this way, both methods train on the same number of timesteps. This analysis could be helpful in showing if PRI-T is better than RTI due to more accurate estimates of targets and/or the use of the confidence estimates. This could be explicitly tested for as well by also comparing to PRI-T with equal confidence weights as was done in Supp Fig 5a/b but now with sweeps of data size.

2.The results for the offline comparisons in lines 177-210, Figure 3, and Supp. Fig 3 and 4 do not contain statistical comparisons between methods. While comparisons of median and IQR can be helpful, it is important to know if differences in methods are statistically significant. At the very least, P-values should be added to the pairwise comparisons in the Supplementary Figures, and indicators of statistically significant differences should be added to Figure 3b.

3.On line 299, the authors state that “our offline, in-silico, and online results demonstrate an unsupervised recalibration system [PRI-T] that outperforms prior state-of-the-art [...].” Authors could be clearer with this claim, for example to specifically say the name of the method (FA stabilizer) and avoid vague statements about state-of-the-art method. Any change should keep line 27 in mind as it uses a similar sentence.

4.On line 104, the authors say “PRI-T appears similar to existing approaches in pairwise recalibrations but outperforms in iterative, longer-term settings.” As PRI-T is not compared to RTI in real data, this statement should be clarified. The authors are referring to distribution alignment methods?

5.It would be helpful to see the heatmaps that are shown in Figure 3 but for the supervised method to give some intuition on the expected upper-bound of performance.

6.While this seems to be implicit, the authors should explicitly clarify how they recalibrate the decoder after the inferred target is obtained from the HMM, write the equation for the linear decoder, and how the weighted least squares is done for recalibration.

Typos
1.There’s a handful of sentences with incorrect capitalization that should be fixed. (e.g. but not limited to line 308)
2.In line 274, Supplementary Figure 4A is probably instead referring to Supplementary Figure 7.

Version 1:

Decision Letter:

Dear Mr Wilson,

Thank you again for submitting to *Nature Biomedical Engineering* your manuscript, "Long-term unsupervised recalibration of cursor BCIs". The manuscript has been seen by 2 experts, whose reports you will find at the end of this message. You will see that the reviewers appreciate the work, and that they raise a number of technical criticisms that we hope you will be able to address. In particular, we would expect that a revised version of the manuscript provides:

*Clarification of the specific improvements conferred by PRI-T.

*Clarification of aspects of the methodology and the conclusions with regard to the data in Supplementary Figure 5, Figure 3 and Figure 4.

*Clarification on the use of specific statistical tests and an additional information regarding multiple comparisons.

*A discussion of the limitations of PRI-T vs RTI.

*Clarification on the hyperparameter selection methodology and application of a independent validation set.

When you are ready to resubmit your manuscript, please upload the revised files, a point-by-point rebuttal to the comments from all reviewers, the reporting summary, and a cover letter that explains the main improvements included in the revision and responds to any points highlighted in this decision.

Please follow the following recommendations:

* Clearly highlight any amendments to the text and figures to help the reviewers and editors find and understand the changes (yet keep in mind that excessive marking can hinder readability).

* If you and your co-authors disagree with a criticism, provide the arguments to the reviewer (optionally, indicate the relevant points in the cover letter).

* If a criticism or suggestion is not addressed, please indicate so in the rebuttal to the reviewer comments and explain the reason(s).

* Consider including responses to any criticisms raised by more than one reviewer at the beginning of the rebuttal, in a section addressed to all reviewers.

* The rebuttal should include the reviewer comments in point-by-point format (please note that we provide all reviewers will the reports as they appear at the end of this message).

* Provide the rebuttal to the reviewer comments and the cover letter as separate files.

We hope that you will be able to resubmit the manuscript within 4 weeks from the receipt of this message. If this is the case, you will be protected against potential scooping. Otherwise, we will be happy to consider a revised manuscript as long as the significance of the work is not compromised by work published elsewhere or accepted for publication at *Nature Biomedical Engineering*.

We hope that you will find the referee reports helpful when revising the work, which we look forward to receive. Please do not hesitate to contact me should you have any questions.

Best wishes,

Michelle

__
Dr Michelle Korda
Senior Editor, *Nature Biomedical Engineering*

____
Reviewer #1 (Report for the authors (Required)):

Thank you for your through response to the previous round of comments.

Reviewer #2 (Report for the authors (Required)):

I thank the authors for performing several new analyses that address important comments. The authors have added a simulation, showing that self-training methods are superior to distribution/subspace alignment methods (Figure 4), with the latter accumulating error over time leading to failure. They have also added 3 sessions of data from another participant to show that self-training works when using personal use data for training, which is also important. I believe the manuscript has in fact improved with these revisions. Regarding the new analyses and results, I have the following comments for the authors to address for better precision and clarity.

1.One main comment is regarding claims added about PRI-T outperforming "state of the art", which the authors have now added to the abstract: "Finally, we show offline that our approach also outperforms the state of the art on freeform datasets of a person using a home computer with an iBCI." I presume "state of the art" implies RTI here, as that is what the authors compare with in the 3 sessions from participant T11. However, I believe this claim is too broad and not sufficiently precise, and thus should either be removed from the abstract (and elsewhere) or made much more specific. Here are the reasons. First, PRI-T and RTI perform similarly in simulations as the authors state on line 277 "Both approaches had similar performance". Only for short training data of less than 2 minutes PRI-T does better in simulations (Fig 4c,e). As I mentioned before, I don't think this data efficiency is that critical because the data is free-use data and thus abundant -- we expect a user to use their BCI freely for more than 2 minutes per day for their daily tasks, indeed we expect hours of free-use. Second, the data from T11 is from 3 sessions, and in 2 of 3 sessions there is no significant difference with sufficient data (session 665 and 672) and in 1 of the 3 sessions there is no difference even for short data (Fig. 6D, left panel, session 665). Similarly, the authors should be more specific in the discussion line 378 what they mean by PRI-T outperforming RTI. If the authors mean improved data efficiency, they should specifically say that and clarify for how much data and how often they see this improvement. A discussion of when such data efficiency is important would also be helpful. I think what is convincingly shown in this manuscript is the general advantage of self-training methods (PRI-T, RTI) over distribution alignment ones, which is an important finding for the field.

2.Related to the above, there are other instances of "state of the art" in the abstract and elsewhere. For example, in the abstract, it is stated "Our approach outperforms the state of the art in large-scale, closed-loop simulations over two months, and in closed-loop with a human iBCI user over one month", where "state of the art" this time likely refers to FA stabilization and not RTI (since RTI was not compared with online). As such the usage of "state of the art" seems inconsistent in the manuscript/abstract. In general, I don't believe there exists an agreed upon unsupervised "state of the art" for recalibration, because BCIs have largely used supervised recalibration thus far and most unsupervised methods are simply tested offline. For precision, I believe the authors should remove such notions of "state of the art" and instead be specific about which methods they are referring to (distribution alignment or self-supervision/RTI) and what the specific improvement is and in what regime it is observed. This will make the conclusions clearer and the manuscript more precise.

3.The authors use 3 separate sessions with participant “T11”. Is there a reason more sessions were not used to provide more general conclusions (see also comment 1 above)? How were these 3 sessions chosen? Also, why not compare with subspace alignment (FA stabilization) on this data offline?

4.In new Figure 4, what is the PD norm (SNR) value that best matches the human data? I assume it corresponds to panels a-

c which are the primary simulations? Please clarify. In Figure 4, the advantage over RTI is at one of the SNR levels (PD norm 0.24), is this a realistic SNR based on human data?

5.I am having difficulty understanding the statistical comparisons in the new Figure 6 and new Supplementary Fig 8. The authors mention "Encouragingly, PRI-T had similar accuracy to the oracle when provided sufficient data (Fig. 6D, right panels)." First, the green and cyan lines seem to be different, how is lack of significance assessed? What is N in these tests and what does each data point (N) represent? Second, how does RTI compare with oracle? Is that in Supplementary Fig. 8? Third, the authors seem to be performing repeated comparisons for each point on the x-axis, which thus requires a correction for multiple comparisons. It does not seem like this correction is applied, is it? Please apply a correction such as Benjamini-Hochberg or False discovery rate control. Finally, when statistical comparisons are pair-wise, why do the authors use the Wilcoxon rank-sum test rather than the signed rank test?

6.I thank the authors for providing the RTI with longer look back, which is interesting. One question I had in my previous comments was whether PRI-T is better than RTI due to more accurate estimates of targets and/or the use of the confidence estimates. As I mentioned previously, this could be explicitly tested for by also comparing to PRI-T with equal confidence weights as was done in previous Supp Fig 5a/b but now with sweeps of data size. I am not sure what the conclusion was regarding that?

7.The authors discuss the advantages of PRI-T vs. RTI, but what are the limitations of PRI-T vs RTI? Is fitting the probabilistic model prone to errors for example if a lot of grid points for target location are needed? Or if the task environment is more general or tasks are more complex? To me one advantage of PRI-T which may be more important than data efficiency is the fact that the user does not need to be actively making selections. Please discuss pros and cons of both methods.

8.The authors perform a new nice simulation in which they let errors accumulate with the subspace stabilization and then apply different recalibration methods, showing that the self-training methods can remove the error but subspace stabilization cannot (Fig. 4e). But in Figure 3C, they conclude that “Results show that PRI-T, ADAN, and FA stabilization all perform poorly in the face of large rotations of the subspace ..., and seem to fail completely for rotations larger than roughly 90 degrees (albeit with differing variability; Fig. 3C).” Are these two conclusions in Fig. 3 vs Fig. 4 consistent? If large rotations lead to failure, how could RTI and PRI-T both correct for it in Figure 4 after error accumulation and thus presumably large rotations? Please discuss how these are consistent results.

9.In terms of hyperparameter selection, it seems the author do so based on performance on test data? For example, the authors state on line 764 that "We select the combination that yields the highest performance on the holdout task blocks (see T11 section)". If so, doing the selection based on test data is prone to overfitting to test data. Instead hyperparameters should be selected based on a validation set that is not used in either training or test, please clarify.

10.I still find the pattern in Figure 3 a bit confusing even now that days apart are shown. For example, the pattern after around day 500 (lower right yellow square) is different from that between day 1-200 (upper left square). What does this imply or mean? It seems to mean that correlations across days suddenly increases after day 500? If so is there a hypothesized reason for that?

Version 2:

Decision Letter:

Dear Dr Wilson,

Thank you for your revised manuscript, "Long-term unsupervised recalibration of cursor BCIs", which has been seen by Reviewer #2. In their reports, which you will find at the end of this message, you will see that the reviewer acknowledge the improvements to the work and raise only a few additional technical criticisms that we hope you will be able to address.

As before, when you are ready to resubmit your manuscript, please upload the revised files, a point-by-point rebuttal to the comments from all reviewers, the reporting summary, and a cover letter that explains the main improvements included in the revision and responds to any points highlighted in this decision.

As a reminder, please follow the following recommendations:

* Clearly highlight any amendments to the text and figures to help the reviewers and editors find and understand the changes (yet keep in mind that excessive marking can hinder readability).

* If you and your co-authors disagree with a criticism, provide the arguments to the reviewer (optionally, indicate the relevant points in the cover letter).

* If a criticism or suggestion is not addressed, please indicate so in the rebuttal to the reviewer comments and explain the reason(s).

* Consider including responses to any criticisms raised by more than one reviewer at the beginning of the rebuttal, in a section addressed to all reviewers.

* The rebuttal should include the reviewer comments in point-by-point format (please note that we provide all reviewers will the reports as they appear at the end of this message).

* Provide the rebuttal to the reviewer comments and the cover letter as separate files.

We look forward to receive a further revised version of the work. Please do not hesitate to contact me should you have any questions.

Best wishes,

Valeria

__
Dr Valeria Caprettini
Associate Editor, *Nature Biomedical Engineering*

____
Reviewer #2 (Report for the authors (Required)):

I thank the authors for addressing my comments. I think the paper has improved and I recommend publication after the below points are addressed:

1) One technical distinction of PRI-T vs. RTI is the confidence weighting as the authors state on line 180. But the new analyses (supp fig 8g) show that this weighting does not necessarily help and can actually hurt performance on the personal use data. This implies that it can be better to use PRI-T without this confidence weighting in some tasks. In particular, the authors state in their response: "Unlike the offline, structured task results with T5 (Supplementary Fig. 5), which suggested that confidence weighting improved PRI-T's performance relative to no weighting ..., the T11 data show a strong improvement without the use of weighting". Currently the methods Line 817 still states that confidence weighting helps and refers to Supplementary Fig. 5A. These statements need to be corrected/amended in light of the new results so that readers know that confidence weighting in some cases can be hurtful and so that they can test for themselves whether they need it or are better off not using it. Also, the current discussion section does not discuss this topic. It should be made clear in discussions and method that confidence weighting is not necessarily helpful and users should compare their PRI-T approach with and without it, with a reference to the new Supplementary figure 8g.

2) In the abstract, it appears by "existing strategies" authors are referring to their closed-loop results in which they compare with distribution-matching methods. Please revise to clarify this.

3) Please clarify superior performance is over FA in this section title: "PRI-T demonstrates superior performance during one month of closed-loop control with T5."

4) On line 456 where pros and cons versus RTI are discussed, please put a discussion of how the number of grid points for target location can be determined for PRI-T and how the increased number of grid points depending on task complexity could potentially affect performance or complexity of method. These are important points for users to know.

Version 3:

Decision Letter:

Dear Dr Wilson,

Thank you for your revised manuscript, "Long-term unsupervised recalibration of cursor BCIs". Having consulted with Reviewers #2 (whose comments you will find at the end of this message), I am pleased to write that we shall be happy to publish the manuscript in *Nature Biomedical Engineering*.

We will be performing detailed checks on your manuscript, and in due course will send you a checklist detailing our editorial and formatting requirements. You will need to follow these instructions before you upload the final manuscript files.

Please do not hesitate to contact me if you have any questions.

Best wishes,

Valeria

__
Dr Valeria Caprettini
Associate Editor, *Nature Biomedical Engineering*

____
Reviewer #2 (Report for the authors (Required)):

The manuscript has been sufficiently revised for publication. I look forward to seeing this out.

Version 4:

Decision Letter:

Dear Dr Wilson and Dr Willett,

I am happy to inform you that your manuscript, "Long-term unsupervised recalibration of cursor intracortical brain computer interfaces using a Hidden Markov model", has now been accepted for publication in *Nature Biomedical Engineering*.

Over the next few weeks, the figures will be checked for production quality, the text edited to ensure that it conforms to house style, and the manuscript typeset.

Our Articles are published about 40 days after the acceptance date (we recommend that you inform your institutional press office of this timeframe), and you will be notified of the actual publication date a few days in advance. Articles can be published any working day of the week, and are pushed live shortly after 10 am London time.

Publishing agreement. You will be asked to digitally sign a publishing agreement (grant of rights). After the signed publishing agreement has been received, the proofs of the article will be sent to you for review. If you have any queries during the production process, or you cannot meet the requested deadline for returning the proofs, please contact rjsproduction@springernature.com.

Nature Biomedical Engineering is a Transformative Journal. Authors may publish their research with us through the traditional subscription access route, or make their paper immediately open access through payment of an article-processing charge. More information about publication options is available.

You may need to take specific actions to comply with funder and institutional open-access mandates. If the work described in the accepted manuscript is supported by a funder that requires immediate open access (as outlined, for example, by Plan S) and your manuscript was originally submitted on or after January 1st 2021, then you should select the gold OA route. Authors selecting subscription publication will need to accept our standard licensing terms (including our self-archiving policies), and these will supersede any other terms that the author or any third party may assert apply to any version of the manuscript.

Acceptance of your manuscript is conditional on agreement, by all authors, with both our media embargo and confidentiality and pre-publicity policies. In particular, you may arrange your own publicity of the Article (for instance, through your institutional press office), as long as you ensure that journalists strictly adhere to the media embargo.

To assist you in disseminating the work, as soon as the Article is published you will be able to take advantage of the Springer Nature SharedIt initiative to generate a unique shareable link to the Article that will allow anyone

(with or without a subscription) to read it. Recipients of the link who are subscribers will also be able to download and print the PDF.

Thank you for having submitted this work to *Nature Biomedical Engineering*.

Best wishes,

Valeria

__
Dr Valeria Caprettini
Senior Editor, *Nature Biomedical Engineering*

Open Access This Peer Review File is licensed under a Creative Commons Attribution 4.0 International License, which permits use, sharing, adaptation, distribution and reproduction in any medium or format, as long as you give appropriate credit to the original author(s) and the source, provide a link to the Creative Commons license, and indicate if changes were made.
In cases where reviewers are anonymous, credit should be given to 'Anonymous Referee' and the source.
The images or other third party material in this Peer Review File are included in the article's Creative Commons license, unless indicated otherwise in a credit line to the material. If material is not included in the article's Creative Commons license and your intended use is not permitted by statutory regulation or exceeds the permitted use, you will need to obtain permission directly from the copyright holder.
To view a copy of this license, visit https://creativecommons.org/licenses/by/4.0/

Reply to Reviewers

Note: reviewers' comments appear in **black text**. Our replies appear in **blue text**, and revised manuscript text appears indented (with old text shown in **black** and new edits in **red**).

We thank the reviewers for their thorough evaluation of our manuscript and for their constructive feedback. Reviewer #1 (R1) noted, "The results are organized effectively... the study addresses an important challenge in clinical BCI, suggesting potential for stable long-term performance." Reviewer #2 (R2) commented, "This paper offers an interesting perspective on the unsupervised recalibration of BCIs... the comparison between self-training techniques and distribution matching/alignment strategies provides a valuable insight. The month-long closed-loop iBCI experiments lend robustness to the findings."

Many of the concerns raised were centered around clarity, elucidation of our methods, and suggestions for expanded analyses. We have made efforts to address each of these points, which we believe has refined our manuscript in both presentation and depth.

R1 underscored the distinction between structured BCI tasks and personal use. R1 rightly pointed out the challenges that may arise with unsupervised recalibration methods in the dynamic, less rigid environment of personal use. Inspired by this observation, we have added new, offline analyses of personal use data in another BCI clinical trial participant ("T11"). Our results demonstrate that self-training on personal use data can yield high quality decoders, and that PRI-T appears generally more data efficient and performant than RTI. We believe that this new analysis not only addresses R1's concerns but also bolsters the applicability of our method in diverse, everyday scenarios (pending final validation with online control).

R2 emphasized the comparative analysis between self-training methods and distribution alignment techniques. In response, we've further developed this section, adding more context and analyses by taking advantage of our closed-loop simulation environment. We demonstrate several key new findings, including 1) better robustness of PRI-T to lower SNR regimes compared to RTI and 2) an analysis of how self-training versus neural realignment methods correct for errors in alignment, where we show that neural realignment methods do not appear to be self-correcting and hence inevitably accumulate error over time, in contrast to self-training methods.

Before addressing each reviewer separately, we'd like to comment on some shared concerns. Firstly, both reviewers raised questions about recalibration in a personal use setting. Toward this aim, we have included new analyses using historical data from another iBCI clinical trial participant ("T11"), who has two, 96-channel arrays in hand knob area of motor cortex. In these "personal use" sessions, T11 used his cursor control system in a home use setting (including typing, web browsing, and e-book reading). Importantly, these data include a wide range of more realistic behaviors, including self-prompted movements, pauses, and incorrect selections.

By recalibrating with PRI-T and RTI using these data and measuring decoder quality on structured task blocks from the same day, we show that both self-training strategies can enable high offline performance - close to a supervised decoder - with sufficient data. Furthermore, we find that PRI-T is generally more data efficient than RTI across sessions. This aligns with our simulation results and bodes well for online translation (where fast recalibration is needed for sustained user engagement). We also found that, on some sessions, PRI-T outperforms RTI even with all available data. This result suggests that strategies such as PRI-T are likely better suited for real-world applications where user behavior is more variable. We have included these results in a new Figure 6, included below:

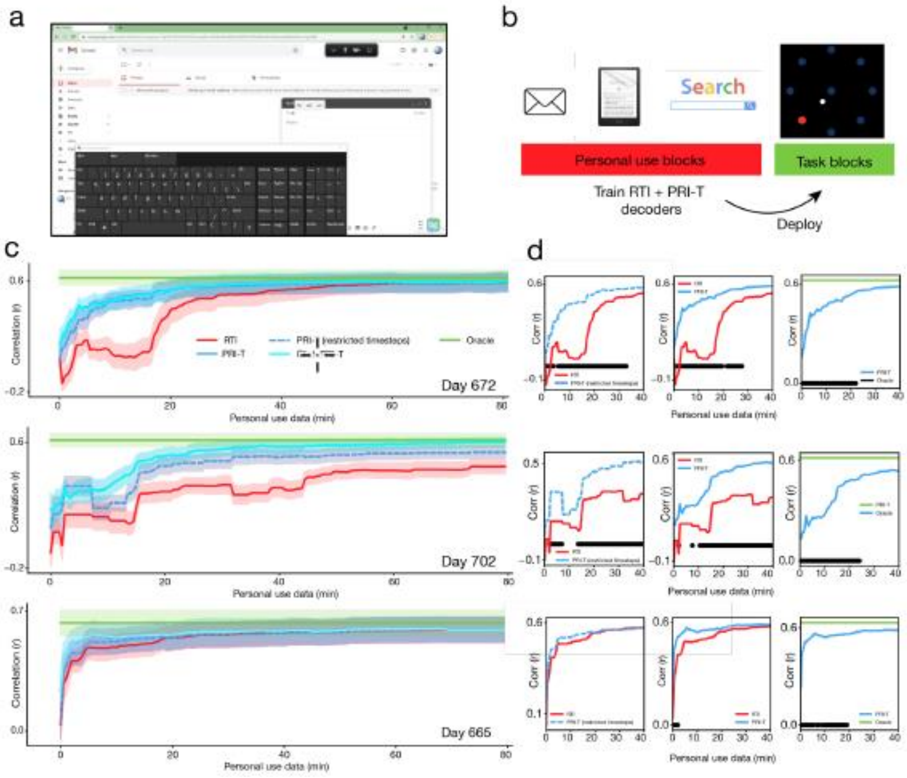

Fig. 6 | T11 offline personal use data. a, Screenshot from a personal use session (“2021.07.27”) during which T11 controlled a desktop computer through 2D cursor control as well as discrete selections (see Methods). **b,** self-training evaluation procedure: training data were collected from personal use blocks, during which T11 engaged in a variety of applications ranging from sending emails to e-book reading to web searches. Cursor and click signals during these blocks were used to train decoders using either RTI or PRI-T, which were then evaluated offline on held-out task blocks from either the start or end of the day. **c,** recalibrated model performance on task blocks with increasing personal use data (evaluated on non-overlapping, 30 second segments; dark lines, mean; shading, SD). Green line denotes a supervised baseline trained on the held-out data (cross-validated using the same segments). **d,** mean performance for different pairs of approaches from **(c)** with black scatters indicating significantly different timepoints ($p < 0.05$, Wilcoxon signed-rank test).

And have modified our Results:

PRI-T outperforms RTI on personal use data in participant T11

To further validate translational viability, we next examined PRI-T’s performance offline in unstructured, personal use settings. Here we used data from a second clinical trial participant (T11) while he performed tasks such as email typing (Fig. 6A), web browsing, and e-book reading. Using cursor and click outputs from these blocks, we then trained linear decoders using RTI and PRI-T and evaluated them offline on held-out, structured task blocks (Fig. 6B). This enables us to retrain in an unstructured setting while still obtaining a ground-truth readout for performance evaluation (via regression against the point-at-target signal during a task with known targets).

For each approach, we measured test block performance as a function of the amount of personal use training data provided (Fig. 6C). We performed a grid search (Supplementary Fig. 9) separately for RTI (blue) and Click PRI-T (cyan; where we augmented the HMM observation model with a click indicator variable). We also

considered a supervised baseline (“Oracle” in green) that is trained and evaluated on test blocks using cross-validation.

PRI-T was generally more data-efficient than RTI across sessions, with significantly higher performance on the test data for training sizes under roughly 40 minutes (Fig. 6C; Fig. 6D middle panels; albeit note session “2021.07.20” showed significant differences only for very small sizes). This improvement appears partly due to better pseudo labels, as restricting PRI-T’s labels to the same timesteps as RTI’s (“restricted timesteps”, dotted blue lines; Supplementary Fig. 9A) still showed differences in performance for two of the sessions (Fig. 6D, left panel). Interestingly, Click PRI-T’s performance curves are nearly identical to the standard PRI-T instantiation (Fig. 6C; Supplementary Fig. 9C,E,G). This is in contrast with simulation results where click was helpful for small dataset sizes (Supplementary Fig. 8A), suggesting that velocity signals alone may be sufficient for unsupervised recalibration. Encouragingly, PRI-T had similar accuracy to the oracle when provided sufficient data (Fig. 6D, right panels). This implies that, with sufficient retraining, PRI-T should be able to achieve a degree of iBCI cursor control competitive with supervised retraining in an everyday setting.

We have also modified the Discussion, where we have moderated our tone with an optimistic outlook for final, closed-loop results needed to fully validate PRI-T:

Alongside our closed-loop results here (28 days), we believe that self-training strategies demonstrate strong potential for long-term BCI stability and are well-positioned for final validation in online, personal use environments.

Strategies such as PRI-T may therefore be crucial for long-term stability in human recordings. Future efforts to quantify the extent of signal nonstationarity differences between animal models and human BCI users, as well as differences between structured tasks and personal use behavior, may enable a more nuanced understanding of the conditions in which recalibration procedures will generalize to human users

Second, both reviewers sought clarity on the visualization in Figure 3A, particularly the block structure in the heatmaps. This visualization depicts recalibration performance for all day-pairings, and has distinct diagonal "blocks" that result from an uneven sampling of days. We recognize that this initial presentation was not immediately intuitive and lead to confusion (our apologies). We've now modified this figure with an additional heatmap showing distance between days for better visual aid, while also changing the axis labels for the main heatmaps to read “Session ID”:

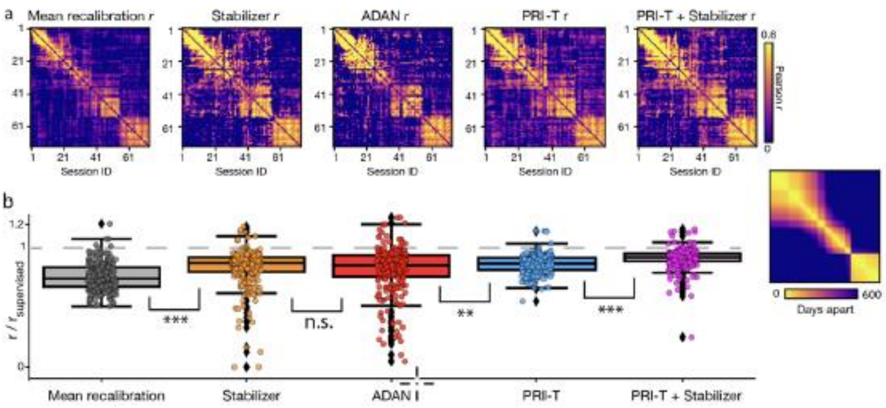

Fig. 3 | All tested methods fail with sufficient tuning drift between sessions. a, Performance of various approaches across different pairs of sessions and plotted as a heatmap. Color indicates decoder output correlations with ground-truth displacement after recalibration. Rightmost lower heatmap shows time between session pairs.

We have also explicitly clarified this in the Results:

Plotting performance across pairs of sessions as a heatmap (Fig. 3A - note uneven sampling of days along axes; an additional heatmap on the right shows time between session indexes), we observed similar overall performance patterns across approaches.

More detailed point-by-point responses to each of the reviewer's comments appear below. We believe that our manuscript has been enriched by these revisions.

____

Reviewer #1 (Report for the authors (Required)):

This study reports an important result that stable BCI performance can be obtained without explicit recalibration for 28 days. This conclusion is supported through online brain-computer interface performance using a new method of automatic recalibration (PRI-T) that leverages models of kinematic task structure to infer target locations. They also compare to other state-of-the-art recalibration approaches both online and offline. The introduction does a nice job of describing the challenges with achieving stable BCI control and the strengths/limitations of various approaches to overcome this challenge. The results are nicely organized with the key conclusions supported in the main figures and details regarding day-by-day comparisons or model optimization are included in the supplemental materials. The findings reported here address an important challenge in clinical BCI and the results are significant because they demonstrate the potential for very long term stable performance with clinically-relevant amounts of data. I commend the authors on their thorough and rigorous study.

We are grateful that the reviewer believes this manuscript is of importance for clinical translation of BCIs and for their in-depth review of our findings and helpful suggestions.

Below I include specific comments that I think would help strengthen this manuscript. The primarily limitation that should be discussed further is that the participant was always performing a structured BCI task where they were highly engaged in a very active task. It remains to be demonstrated that this method would generalize to self-selected and self-paced general BCI use (e.g. typing or browsing the web).

1.It is appropriate to discuss some limitations of the closed-loop experiment results (Figure 5) and/or interpretation in greater detail.

a. First, the participant was always performing the same type of structured task (random target presentation and selection). For this type of task, the target is known and explicit recalibration could be performed. For actual BCI use in a home environment, the user would likely perform less structured tasks that could be more variable (e.g. on screen typing vs. web browsing) that may have different kinematic distributions or requirements. Future work will need to evaluate the extent to which this approach is generalizable to less structured or prescribed BCI use.

Thank you for raising this point - we agree that demonstrating unsupervised recalibration in a personal use setting is important for clinical translation. While our emphasis in this paper was on bridging the gap between offline and online control settings, we recognize that our preliminary success in a structured task setting will still need to translate over to home use. We have added a new Figure 6 tackling personal use, as described at the beginning of this reply, and hope that it sufficiently addresses this point. We recognize that future work is still needed to validate decoders calibrated using personal use data in an online setting.

b. There is a significant gap between Day 14 and Day 28 and the authors comment that neural activity on Day 28 looks quite similar to the reference day so I am not very convinced that the results can generalize to any 28 day period, though this does not change or weaken the conclusions that are stated. Rather, I think that there is an open question about whether this similarity is due to the random walk nature of neural signal instability as proposed, or whether it reflects a return to a particular neural state (e.g. attention level). It may be valuable to verify that the subspace similarity was in fact more similar on this day than on Days 12 and 14. The simulation results assume a continuously increasing amount of instability, which may not be reflective of real world conditions. This is evidenced in the pairwise tests in Figure 3a as well where there is not a continuous decrease in performance for greater separation between trials. The results clearly show that PRI-T performs better than other methods for ever increasing signal instability, which certainly occurs on average over time. I would appreciate if the authors could mention that continuously increasing instability may be a simplification of real world conditions.

Thank you for this comment. We agree that a strict random walk is unlikely for real BCI data and have modified our Results accordingly:

The fixed decoder partially recovers on day 28, implying that the neural activity dimensions found on day 28 have stronger overlap with day 0 than the prior sessions. This is possible given that a random walk model of continuously accruing instability may be a simplification of real-world settings. neural activity evolves in a somewhat random walk fashion, and may thus double back on itself. Nevertheless, PRI-T appears to be the best method for adapting to nonstationarities, as a majority of the time, neural activity will differ substantially from an initial state as time progresses.

Over a long enough period of time, neural activity does nevertheless appear quite distinct from any given reference day (e.g. Fig. 1F: note that correlations in cosine tuning weights drop to zero over time). We caution that Figure 3A has a non-uniform sampling of days along the X and Y-axes (due to e.g. medical absences, COVID-19 related research shutdowns, etc.). Our apologies again for the confusion and we hope that the modified figure 3A mentioned earlier addresses this point.

c. I understand and agree with the authors rationale for using an interleaving block-design for the online decoder comparison. However, this approach may have limited learning or consolidation of a consistent latent neural structure that other approaches may benefit from (such as FA alignment). This limitation should be noted.

Thank you for raising this issue. We agree and have modified the Discussion to include the following sentence:

…will accumulate errors over time without a self-correction mechanism. That being said, FA stabilization may also benefit from consolidation of latent neural activity 11, which could be hindered by our interleaved block design during closed-loop sessions.

Citation #11: Athalye, V. R., Ganguly, K., Costa, R. M. & Carmena, J. M. Emergence of Coordinated Neural Dynamics Underlies Neuroprosthetic Learning and Skillful Control. *Neuron* **93**, 955–970.e5 (2017).

d. For the closed loop experiments, was FA implemented in an iterative manner or always referenced to Day 0? I presume that it was iterative to mimic realistic BCI conditions, but I could not glean this information from the text.

We used the fixed ("static") FA as described in the Degenhart et al paper. Ultimately, we were faced with a catch-22 situation: implementing a novel modification to FA stabilization would potentially be an unfair comparison, as chained FA has not previously been tested in true closed-loop with iBCI participants. The original FA paper uses only this fixed strategy, and similarly offline studies of competing methods have focused mainly on pairwise recalibration (e.g. Ma et al., 2023, Karpowicz et al., 2022).

Nevertheless, the counterargument (perhaps FA stabilization with chaining would do better) does hold weight. To address this, we have included further simulation results analyzing chained subspace stabilization's performance after sufficient error has accrued. We find that, with increasing data, stabilization appears at best only capable of preventing new errors from being introduced over time (as opposed to correcting previously introduced errors). Our simulation results suggest that, in a cosine tuning-like regime, FA stabilization will thus likely fail over longer time periods, as inevitable errors are introduced and accumulate. A copy of the updated figure and accompanying caption are reproduced below:

Fig. 4 | Comparison of recalibration strategies in simulation. **d**, Performance at 30 days out as a function of the simulator's SNR. We simulated 200 repetitions for each method (p-values via two-sided Wilcoxon rank-sum test). **e,** Performance after letting stabilizer errors accumulate. Each method is then run for 5 days across different dataset sizes and the relative decoder error is then plotted (dark lines, means; shading, 95% CIs).

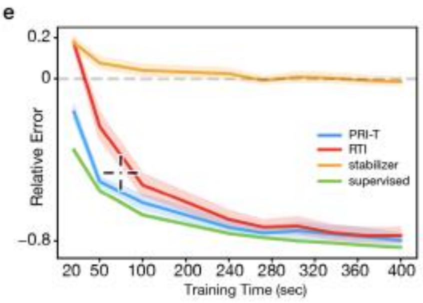

We have also updated the Methods to more explicitly mention this detail:

Initial day.

…the subspace decoder, as well as the associated FA model, are used as the reference session for FA stabilization on each new day (this is a "fixed" stabilization strategy).

As well as the Discussion (relevant new text is underlined):

More immediately, this failure mode would explain FA stabilization's poor performance offline on pairs of sessions with large subspace angle changes (Fig. 3), in our closed-loop simulations (Fig. 4), and during closed-loop control in T5 on day 12, which had the largest neural nonstationarities with respect to day 0 (Fig. 5). While chaining stabilization across time may prolong performance in future closed-loop studies, our simulation results suggest that errors should accrue until control is no longer possible (Fig. 4).

2.Figure 1e includes many sessions where the R2 values is very low, which likely implies nearly unusable BCI decoding. The inclusion of these sessions also obscures the main results described in the text reporting median bias values <1.

The offline analysis here considers using a decoder while not accounting for bias changes that can result in a low R2 (hence the tendency of far apart days with large bias index values to have low R2s). The performance within-day on these sessions was generally good as decoders were built on those days.

3.The authors mention that the lack of confidence estimates for pseudotargets is a limitation of RTI. There is only one example figure (Figure 2d) of confidence estimates for PRI-T but it is a bit difficult to interpret what is being shown. The different colored traces are not defined. The confidence estimates seem to rapidly move from 0 to 1 for these structured tasks. Would this be the case for an unstructured task?

The confidence estimates show the occupation probabilities for each timestep P(H_t | O_1, …, O_T), i.e. the conditional probabilities of the target states given the observed cursor data. With 400 such traces (one for each target state), it is difficult to add an appropriate legend. We have updated the figure caption however to better clarify their meaning:

Fig. 2 | FA stabilization and PRI-T overviews. … **d,** We also query the model for its confidence in its predictions by obtaining the marginal probability of the target state , plotted here as colored lines (one for each possible target state), and use this to weight the importance of each pseudo-target label during recalibration.

Below is an example of the pseudotargets from Click PRI-T during one of T11's personal use sessions. Note that, while there are some characteristic "peaks" and high confidence time segments, the latter half of the plot shows relatively little confidence on PRI-T's part regarding target states. This suggests that more varied user behavior during personal use will be reflected in the pseudolabel probabilities.

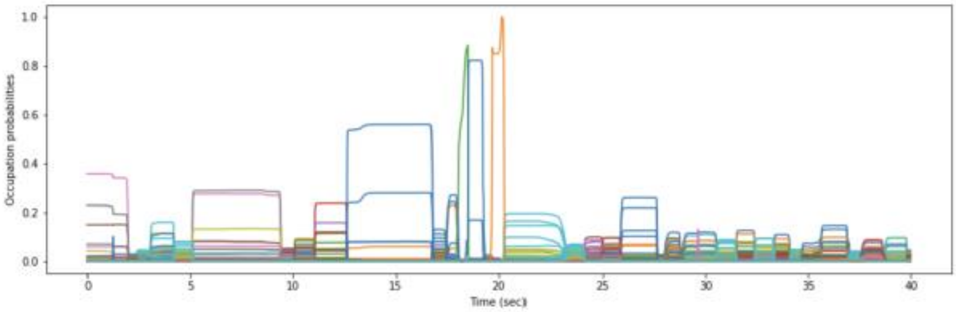

4.This statement seems to be inaccurate: "Both latent space methods tended to have much larger IQRs ([0.83 - 0.96] for stabilizer; [0.89 - 0.98] for ADAN) compared to PRI-T ([0.85 - 0.96])." The confidence interval for PRI-T falls in between the two latent space methods.

Our apologies for this mistake. Our intended point was an observation that the overall variability of latent space methods at the extremes was greater; however, the IQRs are indeed similar. We have modified the language accordingly:

> Both latent space methods ~~tended to have much larger IQRs~~ appeared to have more variability at the extremes, albeit with similar IQRs ([0.83 - 0.96] for stabilizer; [0.89 - 0.98] for ADAN; [0.85 - 0.96] ~~compared to~~ for PRI-T).

5. I was unable to come up with an explanation for the differing amount of variability shown in Figure 3c. The authors do mention this in the text, but I am curious if there is a hypothesis as to why this was observed.

Thank you for raising this point. Our interpretation is that, because PRI-T is reliant on the decoder output to be able to infer target locations, PRI-T's performance is largely determined by the decoder's baseline performance on a new day (which is implicitly quantified by the x-axis, which is a measure of decoder angle differences between two days). Thus, PRI-T's performance does not have large variability as a function of decoder angle difference. By contrast, latent space methods are contending with an identifiability problem: how one should optimally rotate a subspace on a new day to match the reference subspace. This latter problem is influenced by other factors not included in the subspace angle metric on the x-axis, such as neural activity variations in readout-null subspaces (e.g. FA stabilization uses n_components=6, only two of which are reflected in the subspace angle metric). Since distribution alignment methods are influenced by other factors not reflected by the decoder angle difference, they may thus have larger variability in performance as a function of decoder angle difference.

6.It was unclear how many "targets" (e.g. the n x n spatial grid) were considered in the PRI-T model. Would the number of targets inferred from the various structured BCI tasks be similar to the number of targets that would be appropriate for self-selected tasks during BCI-controlled computer use?

We used 20 x 20 = 400 possible target locations distributed equally across the screen. Note that this was chosen independently of any target layout. Our simulation and closed-loop results (and many of the offline T5 datasets) are a random targets task, which means PRI-T's discretization cannot rely on overfitting the discretization to any specific structure in target locations. More specifically on this question, we used the 20 x 20 discretization option on T11's personal use data with good performance. This indicates that 20 x 20 appears sufficient for personal use on a standard computer monitor (albeit there are likely to be performance gains from sweeping this parameter).

7.The results presented for the click dimension were quite limited. It was unclear how click targets were defined particularly given the combination of hover- and click-based control. Based on the methods, I interpret that the click probability was high when the cursor was near the target (which was also estimated probalistically), but again I wonder how well this would generalize to less structured tasks. The paper would benefit from a more detailed description of how the click targets were defined and showing data regarding the confidence estimates, predicted click timing, and actual click timing.

Thank you for this note and apologies for the confusion. In our simulation, we use dwell-based clicks (500 ms hover over targets) rather than neurally-evoked clicks for simplicity and ease of implementation. In the personal use data, T11 uses gestures to emit discrete clicks. Given the personal use nature of those data, we're unable to compare predicted clicks (on the part of the system) with ground-truth intended clicks (which one could infer by measuring when the user crosses over onto a target). We have added language to more clearly delineate these forms of click control in the Methods:

> **Closed-loop simulator** This signal is exponentially smoothed with the prior velocity (= 0.94) and scaled by a fixed gain (determined via sweeps, described below) before updating the cursor state. We used a dwell-based target selection with a hold time of 500 milliseconds.

And in the Results:

> **Simulations show that only target labeling strategies maintain long-term control.** We then simulated closed-loop control with dwell-based target selections over two months in the presence of a drifting encoding subspace.

> ***In-silico* data efficiency robustness of PRI-T and RTI.** …We also evaluated the performance of click-augmented PRI-T (see Methods), where an explicit click observation model is integrated into the HMM to account for dwell-based selections in the simulation.

8.Were the subjects informed that different decoders were being evaluated? Did they report any subjective performance differences between the recalibration methods?

T5 knew that different decoders were being evaluated in each block, but was not told ahead of time which decoder was being used. Nevertheless, the large performance difference between decoders meant that he was likely able to infer which decoder was being used. During closed-loop sessions, T5 reported much more satisfaction with PRI-T based control compared to both other decoders.

9.I appreciate that the authors were transparent regarding their process for hyperparameter selection as this will obviously impact how well each method performs.

Thank you - we also plan on releasing the T5 cursor control dataset to enable others to replicate the performance shown here.

10.There were a number of instances where IQRs were reported without units. We have updated the IQR reporting in the Results section.

11.Minor: The first paragraph of the methods refers to “participants” but only 1 participant is included in this paper.

Thank you for this correction and our apologies for not catching it. With the new T11 data in mind, we have modified the language to:

> Research sessions were conducted with volunteer participants enrolled in the BrainGate2 pilot clinical trial (ClinicalTrials.gov Identifier: NCT00912041). The trial is approved by the U.S. Food and Drug Administration under an Investigational Device Exemption (Caution: Investigational device. Limited by Federal law to investigational use) and the Institutional Review Boards of Stanford University Medical Center (protocol #20804), Brown University (#0809992560), and Massachusetts General Brigham(#2009000505).
>
> Participant T5 is a right-handed man who was 69 years old at the time of the study. He was diagnosed with C4 AIS-C spinal cord injury eleven years prior to this study. T5 is able to speak and move his head, and has residual movement of his left bicep as well as trace movement in most muscle groups. T11 is a 37-year-old right-handed male with a C4 AIS-B spinal cord injury (SCI) that occurred approximately 11 years prior to study enrollment. Both T5 and T11 gave informed consent for this research and associated publications.

Reviewer #2 (Report for the authors (Required)):

This paper shows a new approach for unsupervised recalibration of BCIs based on self-training, without the need for supervised recalibration sessions which require users to interrupt their free use of the BCI (free-use session). As a key strength, the study compares methods in actual closed-loop iBCI experiments for one full month, which is commendable. Unlike this study that focuses on a self-training approach and closed-loop iBCI testing, recent work for unsupervised BCI recalibration have instead focused on distribution matching/alignment approaches with largely offline testing.

The authors develop a new self-training method called PRI-T, which provides an explicit probabilistic HMM model for target inference unlike the prior self-training method of RTI. The HMM model relates targets to cursor kinematics based on a first order Markov chain and uses the forward-backward and Viterbi algorithms to estimate the targets from cursor observations along with the probabilities of the targets during free-use sessions. These target estimates can then be used to recalibrate decoders for subsequent free-use sessions. The method is evaluated in offline analyses of human iBCI data, closed-loop simulations, and critically on closed-loop human iBCI data. Most interestingly, the authors show that self-training improves distribution alignment in closed-loop iBCI experiments over one month of usage. I believe this is the first demonstration of this important result.

The topic of addressing nonstationarity in neural data in iBCI is important. I believe the authors’ result that self-training methods outperform distribution matching/alignment techniques (such as FA stabilization, ADAN, NoMAD) is a very valuable contribution to the field. This is because distribution alignment methods have shown no or minimal validation in actual closed-loop iBCI experiments as the authors correctly note; instead, alignment methods have largely focused on offline testing, which can be quite different from real-world iBCI use by a human. Here, the authors compare on a full month of closed-loop iBCI use, which is commendable. Overall, convincingly demonstrating that self-training (rather than distribution alignment) is the way to go for practical human iBCIs will be a very important contribution and save the field a lot of time by showing the direction to focus efforts on. For that reason, I believe the authors should focus their manuscript on this finding and expand their

analyses on it (for example as I suggest below) to make the manuscript even stronger in this respect. The specific self-training method here (PRI-T) is also interesting in that it provides a mathematical formalization of self-training ideas; but to me the general finding that self-training far outperforms distribution alignment is more significant and should be expanded with further analyses. With the below revisions, I believe the paper will be a very valuable contribution.

Major Comments

1.I find the result that self-training far outperforms the distribution alignment/matching methods (both FA stabilization and ADAN) the most important contribution of this study. Indeed, it seems that distribution alignment performs poorly in closed-loop iBCI, almost similar to a fixed decoder. It is thus critical to strengthen this finding as I discussed above. I think the authors have an opportunity to show the reasons why self-training is advantageous for iBCIs with their closed-loop simulation setup.

Thank you for this comment. We have attempted to address this with a new analysis described below.

The authors hypothesize the reason for FA performing poorly in closed-loop iBCI is that “the alignment step may fail if the underlying task manifold is too symmetrical to enforce a unique identification”. Also, they show ADAN underperforms and overfits, leading to more variability compared with FA. Further, they state a hypothesis of noise-dependence, saying “One exception is FA stabilization, which was evaluated over a 5 day period in NHPs, and may be optimal for a specific noise regime experienced in shorter timeframes”. I think Degenhart et al hypothesize that FA’s advantage over supervised methods holds for periods of low user engagement, which may not happen during free-use of a human user unlike an NHP. The closed-loop simulations here provide a good setup to test such hypotheses by simulating different scenarios for the noise, drift, etc. Similarly, ADAN can be trained with more or less data to confirm the above hypotheses about overfitting. Also, hypotheses about other recent distribution alignment methods can be posed and/or tested in closed-loop simulations. I think this is the most important aspect the revision should focus on: expanding on the downsides/comparisons of distribution alignment approaches in general (for iBCIs), which are largely not tested in closed-loop iBCI but rather on offline data.

Thank you for this comment. Indeed, a major point of our paper is that self-training appears to be more viable as a class of solutions than distribution alignment. Toward that aim, we have included a new analysis in which we allowed subspace stabilization to accrue errors until decoder performance deteriorated. Upon applying subspace stabilization, RTI, and PRI-T, we observed that while subspace stabilization mainly worked to minimize new error accumulation, self-training methods (PRI-T and RTI) can additionally remove prior accumulated errors. This can be visualized in a newly subpanel for Fig. 4 (reproduced below alongside the new section discussing this result). Based on this result, we propose that distribution alignment approaches fail over the long term as they accumulate errors over time, in contrast to self-training methods which are capable of correcting previously introduced calibration errors.

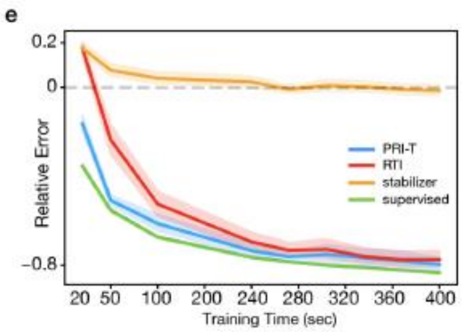

Fig. 4 | Comparison of recalibration strategies in simulation. d, Performance at 30 days out as a function of the simulator’s SNR. We simulated 200 repetitions for each method (p-values via two-sided Wilcoxon rank-sum test). **e,** Performance after letting stabilizer errors accumulate. Each method is then run for 5 days across different dataset sizes and the relative decoder error is then plotted (dark lines, means; shading, 95% CIs).

Error-accumulating nature of chained subspace stabilization. Chained subspace stabilization involves composing multiple subspace transformations together, which might introduce compounding errors that could explain the subpar performance we observed relative to target-labeling methods. To investigate this, we first ran independent simulations of chained subspace stabilization until errors reached a threshold (see Methods). After this point, we then considered the effects of applying different recalibration methods for different dataset quantities, to see if any methods were capable of reversing the accumulated error when given enough training data. Here, “error” was defined using the correlation between decoder weights and the population encoding matrix.

We found that, despite increasing dataset size, subspace stabilization was unable to remove prior errors in the decoder estimate (Fig. 4E; shading denotes bootstrapped 95% CI). At best, subspace stabilization appeared to only avoid adding new errors into the system if given large amounts of training data. By contrast, self-training strategies such as RTI and PRI-T were able to compensate for previously induced errors, driving the error far below where it started. Similar to our data efficiency sweeps (Fig. 4B), PRI-T generally outperformed RTI for very small dataset sizes (< 100 seconds here). These results offer an explanation for why subspace stabilization fails over the long term: errors are inevitably introduced with each new update and compound overtime, with no mechanism available for correcting the error.

2.Another contribution here is the development of the new PRI-T method for self-training. Indeed, there are novel differences in methodology in PRI-T vs. the prior RTI method for self-training. Given that at a high-level, both PRI-T and RTI estimate targets from cursor data for recalibration purposes, extending the comparisons can further highlight the advantages of PRI-T. Currently, comparisons to RTI are done in the simulations. How do the methods compare in the offline iBCI data? I appreciate that the closed-loop experiments have already concluded and cannot be repeated but it is also helpful to put a discussion on what the expected performance differences could be.

Thank you for raising this point. Based on the newly added T11 personal use data, we find evidence that PRI-T is more data-efficient than RTI and can also result in higher quality performance even with large amounts of data (> 40 minutes).

3.In the simulations, the main advantage of PRI-T vs RTI is noted as requiring less data (about ~2 min less, Fig. 4f). Given that this data seems to be free-use data, the authors should clarify how much of a real-world advantage this provides over RTI and in what situations. Are there situations where free-use data is limited? The authors should highlight such situations. Again, that is why to me item 1 above – showing that self-training in general, regardless of specific method, is better than distribution alignment for iBCIs– is more significant of a contribution and should be emphasized, focused on, and further explored.

Thank you for this comment. We have included expanded simulation analyses to further contrast PRI-T with RTI, shown below in an updated Figure 4:

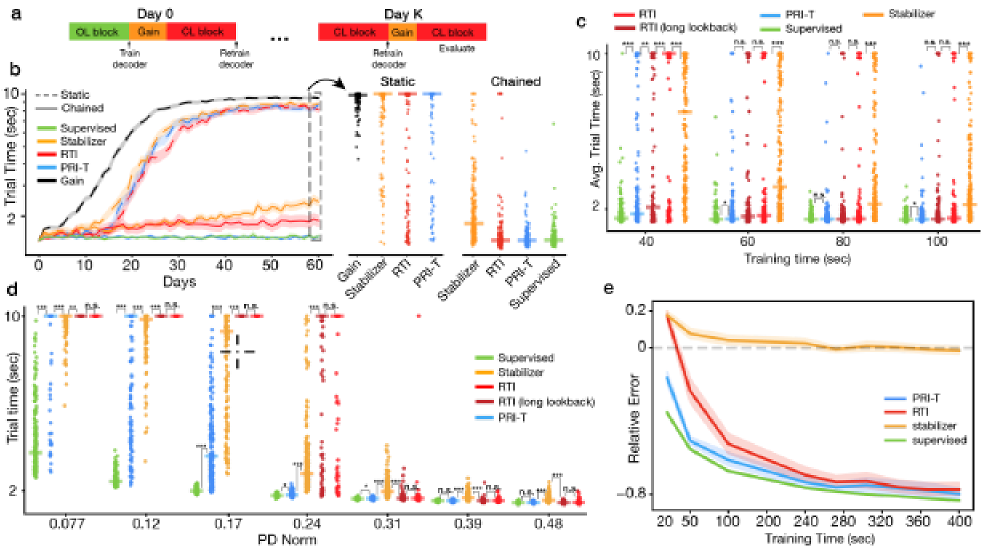

Fig. 4 | Comparison of recalibration strategies in simulation. a, overview of multiday recalibration experiment. Decoders are trained on open and closed-loop data on a reference day, then tested in closed-loop simulation on a new day. Recalibration is then applied, followed by a gain adjustment, before measuring performance on another closed-loop block. **b,** Left: average trial times across sessions for different methods (400 seconds or 6.7 minutes of data per block). Shading corresponds to standard error over 200 independent runs. Right: comparison of trial time values between methods at 60 days out. **f c,** Performance of RTI, PRI-T, and supervised recalibration as a function of training data size with 100 independent runs each per data size tested (two-sided Wilcoxon rank-sum test). Trial times measured at 30 days out. **d,** Performance at 30 days out as a function of the simulator's SNR. We simulated 200 repetitions for each method (p-values via two-sided Wilcoxon rank-sum

test). **e,** Performance after letting stabilizer errors accumulate. Each method is then run for 5 days across different dataset sizes and the relative decoder error is then plotted (dark lines, means; shading, 95% CIs).

In addition to data efficiency, we also show that PRI-T is more robust to lower SNR signals. This is because, as the number of clicks reduces due to fewer successful trials, RTI is unable to operate efficiently (Fig. 4D). As a result, even subspace stabilization overtakes RTI (starting around the middle of 4D plot) since it can rely on all neural signals and not just those associated to a window preceding clicks.

We also demonstrate increased data efficiency on real personal use data (Fig. 6), and even overall better performance with large amounts of data for one session (~40 minutes).

Considering only data efficiency alone as an advantage, we believe that this attribute is crucial for real-world adoption. Current supervised methods can achieve high performance within minutes (e.g. Brandman et al., 2018). If equipped with a mediocre decoder, the main restriction on unsupervised recalibration is likely to be a user's tolerance for suboptimal control when first initiating control. By reducing data requirements, PRI-T may enable higher acceptance of unsupervised retraining for across-day recalibration (by shortening the time one has to cope with subpar performance).

Regarding the distinction between self-training and distribution realignment however, we completely agree that this is a crucial fork in the road for neurotech development efforts. Toward this aim we have included a new analysis of error reduction differences (referenced earlier) as well as some updated language in the Discussion:

> More immediately, this failure mode would explain FA stabilization's poor performance offline on pairs of sessions with large subspace angle changes (Fig. 3), in our closed-loop simulations (Fig. 4), and during closed-loop control in T5 on day 12, which had the largest neural nonstationarities with respect to day 0 (Fig. 5). While chaining stabilization across time may prolong performance in future closed-loop studies, our simulation results suggest that errors should accrue until control is no longer possible (Fig. 4).
>
> …
>
> If each iteration of the stabilizer introduces an independent estimate of the optimal rotation angle from day-to-day, then the final transformation (which is composed of these smaller mappings) will accumulate errors over time without a self-correction mechanism. This aligns with our simulation results that demonstrate an inability to remove prior errors on the part of subspace stabilization (Fig. 4E). That being said, FA stabilization may also benefit from consolidation of latent neural activity11, which could be hindered here by our interleaved block design during closed-loop sessions.

Citation: Brandman et al. Rapid calibration of an intracortical brain-computer interface for people with tetraplegia. J Neural Eng. 2018 Apr;15(2):026007. doi: 10.1088/1741-2552/aa9ee7. PMID: 29363625; PMCID: PMC5823702.

4.As the authors discuss, the use of simulations allow for more direct comparisons between methods as well as enable explorations of when one method outperforms the other. Additional simulations that can highlight how PRI-T can utilize the HMM formalization to yield meaningful and practical improvements could help strengthen the manuscript.

Thank you for this suggestion on how to strengthen the manuscript. We are excited about the possibility of further extensions to the model that could yield performance improvements. Since PRI-T is a structured probabilistic model, it is easy to incorporate other sources of information about the user's target in a principled way, for example a decoded click signal in addition to decoded cursor velocity. We have included a further analysis of our "Click PRI-T" variant in simulation (added as part of an updated Supplementary Figure 7), which demonstrates a performance improvement in low SNR/sample size regimes:

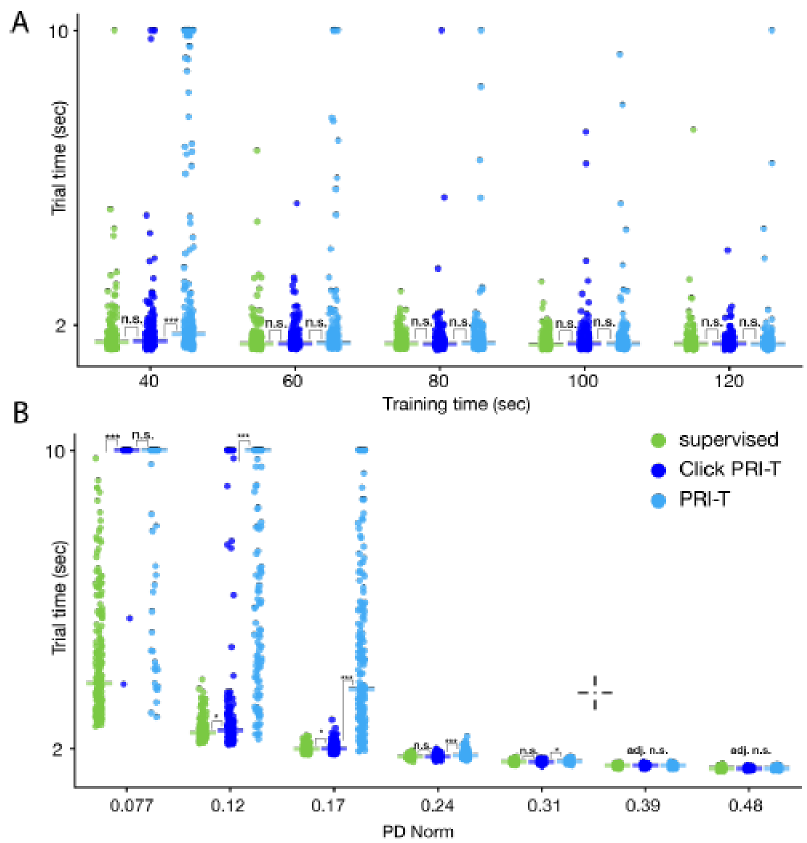

Supplementary Fig. 8 | Adding click to PRI-T. a, Simulated data efficiency comparison of supervised recalibration, PRI-T, and PRI-T with an additional click signal integrated into its observation model. **a,** Average trial times across 200 independent runs (per method and recalibration block size) are measured at 30 days out. two-sided Wilcoxon rank-sum test. **b,** Again simulating performance at 30 days out while varying the population SNR (norm of encoding matrix, see Methods). Recalibration blocks are fixed at 400 seconds long.

5.Comparisons between recalibration methods in closed-loop simulations (Figure 4) currently show both "chained" and non-chained forms of the Stabilizer method where the non-chained appears to use the decoder from day 0 and recalibrate with data from day N while the chained compounds recalibrations over each consecutive day. However, it appears that only the equivalent "chained" forms of RTI and PRI-T methods are used here (as indicated by line 816 where decoders are recalibrated from the immediate prior day rather than only from day 0). While showing the non-chained FA Stabilizer method gives insight into how the method may fail over long time scales, there is then a question of whether the other two self-training methods (RTI, PRI-T) would also fail if non-chained. Analyses with non-chained RTI and PRI-T should be included and discussed.

Thank you for raising this point. We have now included an analysis of the chained and non-chained variants of RTI and PRI-T in our simulation model as well. The non-chained ("static") variants become increasingly unstable as time progresses, which is consistent with our offline analyses in Fig. 3 that demonstrates the need for sufficient decoder overlap to enable self-training on a new day. The new panel for Fig. 4 is reproduced below:

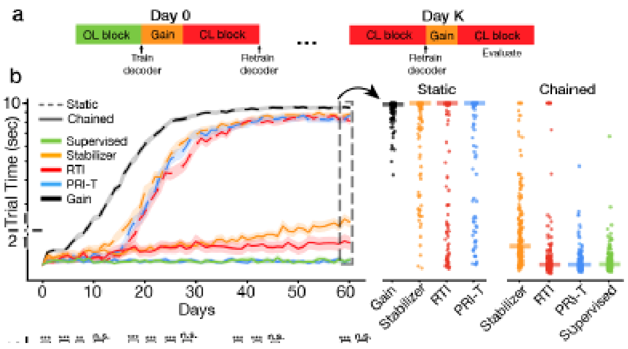

We have modified the Results and Discussion sections accordingly:

Simulations show that only target labeling strategies maintain long-term control.

… We also simulated three unsupervised recalibration procedures: PRI-T, RTI, and FA stabilization (with hyperparameter searches for all, see Supplementary Fig. 2). We found that all procedures FA stabilization were prone to failure ~~noise~~ as time passed between the reference and new day's data, causing poor performance, ~~eventually yielding median performance similar to gain optimization alone. The (with trial times of 8.73 2.52 seconds (p = 0.57, Wilcoxon rank-sum).~~ at the 60 day mark (8.58 2.62 seconds for stabilizer in dotted orange, 8.37 3.09 seconds for RTI in dotted red, and 7.91 3.36 SD seconds for PRI-T in dotted blue). Median performance was statistically significant compared to gain optimization for RTI ($p < 0.05$, Wilcoxon rank-sum) but ~~Median performance was~~ similar ~~to gain optimization alone~~ not for FA stabilization ($p = 0.57$) or PRI-T ($p = 0.42$).

Despite stability improvements, chained subspace stabilization still showed overall high variability, with only chained target inference approaches (RTI and PRI-T) enabling consistent long-term control in simulation.

We have updated the Methods with new hyperparameter sweeps for non-chained RTI and PRI-T as well as the following text:

Simulator hyperparameter sweeps. We tested three unsupervised recalibration methods in simulation (PRI-T, FA stabilization, and RTI) as well as a simple gain recalibration baseline where decoder weights were fixed and only the gain was optimized. For the three unsupervised methods, we separately examined iterative or "chained" recalibration strategies versus a static approach where the reference decoder is fixed to the very first day.

6.In Figure 3a, there seems to be a persistent block structure in the heatmaps (specifically one block around ~500 to ~1200 days, and one block from ~1200 to the last day). One could interpret this as the recalibration methods improving decoding performance within each block but suddenly and abruptly failing when moving to the other block (e.g., around day 1200). Why is that? Could this be due to some unknown confounding variable that is shared within but not between blocks? This adds difficulty in assessing whether recalibration methods fail due to the variable time or due to some other variable that has not been mentioned (e.g., what is happening right around the end of one block?). Additional information and some discussion on why this pattern is there would be helpful.

We apologize for the lack of clarity in the heatmaps. As discussed in the beginning of this reply letter, the days have uneven sampling which leads to a block-ish appearance in the performance matrices. We've updated Figure 3 to make it clear that the discontinuous blocks appear to be caused by large gaps of time between the days.

Other Comments

1.In the dataset size sweeps in the simulations as shown in Figure 4f, PRI-T is shown to outperform RTI with less data. However, inherently RTI uses less data to train since it discards timesteps for various conditions like being outside the lookback window. It would be interesting to see how PRI-T performs with respect to a modified RTI that uses all timesteps (in other words a varying look back window that extends from prior target to current target selection). In this way, both methods train on the same number of timesteps. This analysis could be helpful in showing if PRI-T is better than RTI due to more accurate estimates of targets and/or the use of the confidence

estimates. This could be explicitly tested for as well by also comparing to PRI-T with equal confidence weights as was done in Supp Fig 5a/b but now with sweeps of data size.

Thank you for this suggestion. We have included an updated RTI hyperparameter search (Fig. 4) that includes a look back window for 10 seconds (equal to the maximum trial time). This modified RTI ("long lookback") appears more capable under low dataset settings but nevertheless lags behind PRI-T in performance (Fig. 4B). This modified RTI is also sensitive to low SNR days, as evidenced by our SNR sweep results in Fig. 4D.

We have added some new language in the Results section concerning this:

> ***In-silico* data efficiency robustness of PRI-T and RTI.** As RTI and PRI-T were close in performance, we then looked at the data efficiency of each method by measuring performance after 30 days of unsupervised recalibration while varying the amount of training data provided
>
> (Fig. 4B). Both self-training methods were far more robust compared to subspace stabilization, with significantly faster trial times across all tested dataset sizes ($p < 0.001$ for 60-100 seconds, Wilcoxon rank-sum test), except for the smallest dataset size. At this point, the stabilizer showed higher performance than RTI ($p < 0.001$) but was still worse than PRI-T ($p < 0.001$). We reasoned that this may be due to RTI's lookback window being too small, resulting in insufficient data for retraining. We hence introduced a modified RTI with a larger lookback window ("RTI (long lookback)" in dark red; see Methods) and found that it outperformed subspace stabilization ($p < 0.001$) but still fared worse than PRI-T at 40 seconds ($p < 0.01$).

2.The results for the offline comparisons in lines 177-210, Figure 3, and Supp. Fig 3 and 4 do not contain statistical comparisons between methods. While comparisons of median and IQR can be helpful, it is important to know if differences in methods are statistically significant. At the very least, P-values should be added to the pairwise comparisons in the Supplementary Figures, and indicators of statistically significant differences should be added to Figure 3b.

We have added p-values to Figure 3B, reproduced below alongside the updated Results:

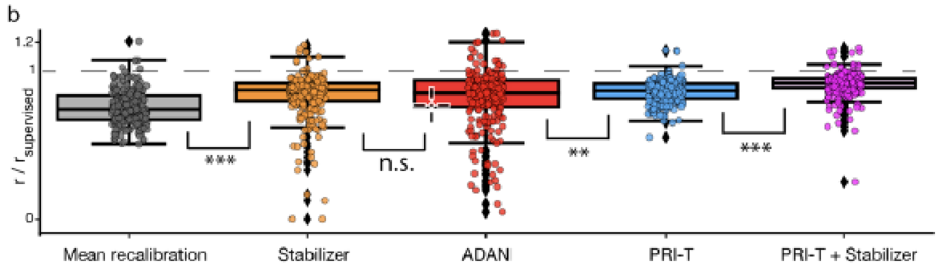

Fig. 3 | All tested methods fail with sufficient tuning drift between sessions. … b, Box plots showing relative performance compared to supervised recalibration (values at dotted line are equivalent to supervised). Center line, median; box limits, upper and lower quartiles; whiskers, 1.5x interquartile range; diamond point, outliers. Pairwise comparisons performed with Wilcoxon rank-sum test.

Sensitivity of existing recalibration strategies to long-term PD changes. To benchmark PRI-T against existing approaches, we examined robustness across multiple timescales via offline analysis of session pairs. Since neural nonstationarities generally increase with larger gaps between sessions (Fig. 1E,F), it is possible that recalibration systems might perform well in the short-term, when such changes are small, but fail in the long-term.

We analyzed this possibility using a cursor control dataset from participant T5 spanning roughly five years and generated pairs of sessions (see Methods) for evaluating recalibration performance. For each pair, a decoder was trained on the earlier session and tested on the newer session after separately applying five methods: supervised recalibration, mean recalibration, FA stabilization, ADAN, and PRI-T. Plotting performance across pairs of sessions as a heatmap (Fig. 3A - note uneven sampling of days along axes; an additional heatmap on the right shows time between session indexes), we observed similar overall performance patterns across approaches. For a more direct comparison, we normalized scores against supervised recalibration's performance

and plotted the data side-by-side (Fig. 3B). Here we subselected days where mean recalibration (normalized *r2* 0.78, [0.70- 0.87]; median, IQR) alone had some baseline performance (see Methods). All methods generally outperformed mean recalibration ($p < 0.001$ Wilcoxon rank-sum test), implying that all methods were successfully accounting for some of the PD changes as well as mean changes on this subset of days. FA stabilization, ADAN, and PRI-T all clustered around 0.89-0.91 in median scores but with variable spread. Both latent space methods tended to have much larger IQRs appeared to have more variability at the extremes, albeit with similar IQRs ([0.83 - 0.96] normalized *r2* for stabilizer; [0.89 - 0.98] normalized *r2* for ADAN; [0.85 - 0.96] compared to normalized *r2* for PRI-T). PRI-T and FA stabilization had similar median performance ($p = 0.069$, Wilcoxon rank-sum test). We also tested a simple ensembling model (0.96, [0.93 - 0.99] normalized *r2* median and IQR; Fig. 3B), which averages decoder predictions from PRI-T and FA stabilization, that provided the highest relative performance across methods ($p < 0.001$ all, Wilcoxon rank-sum test) in this single recalibration setting. Pairwise comparisons across methods had similar findings, with all unsupervised methods outperforming mean recalibration and otherwise appearing equivalent aside for the ensembled approach (Methods; Supplementary Fig. 3). These results were consistent when examining R2 as well (Supplementary Fig. 4).

We have also added p-values to the supplementary figures:

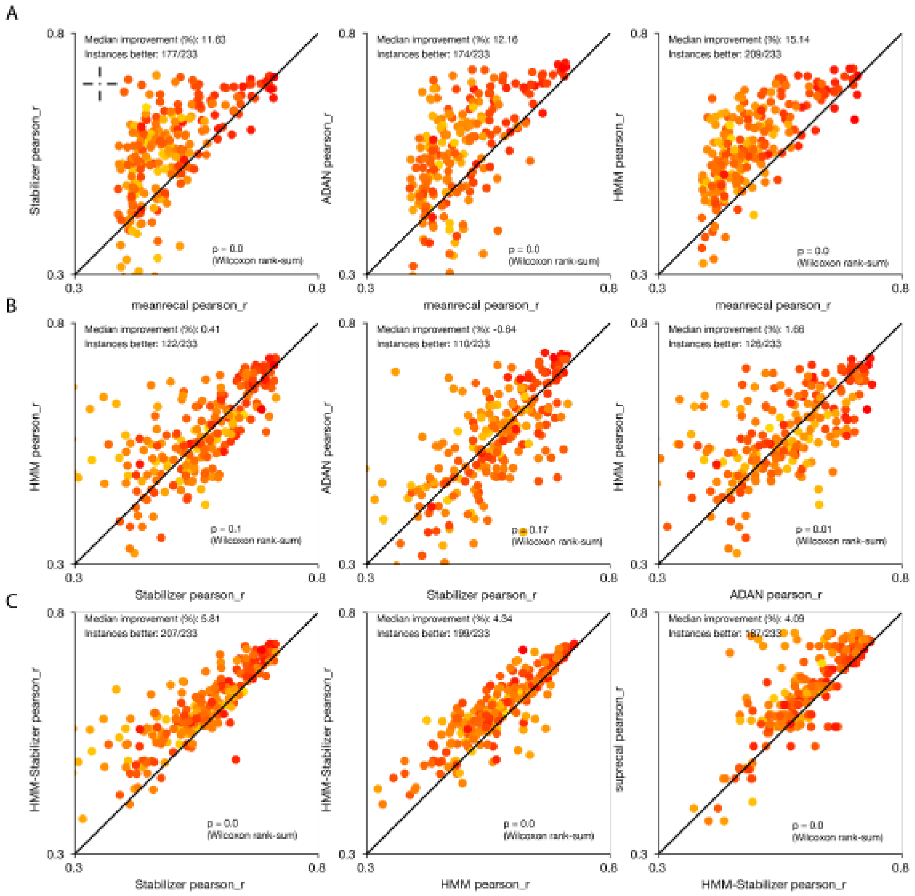

Supplementary Fig. 3 | Pairwise offline results (Pearson correlation). a, Pairwise comparisons plotted for PRI-T, FA stabilizer, ADAN against mean recalibration. PRI-T, stabilizer, and ADAN all generally outperform mean recalibration (> 75% of cases for all three, $p < 0.001$ all Wilcoxon rank-sum test). **b,** Comparisons among PRI-T, ADAN, and FA stabilizer. Methods are all roughly equivalent. **c,** Combined approach outperforms versus PRI-T and stabilizer individually (>85% of the time in both cases). Combined decoder is slightly worse than supervised recalibration (supervised outperforms in ~80% of cases with a median 4% improvement).

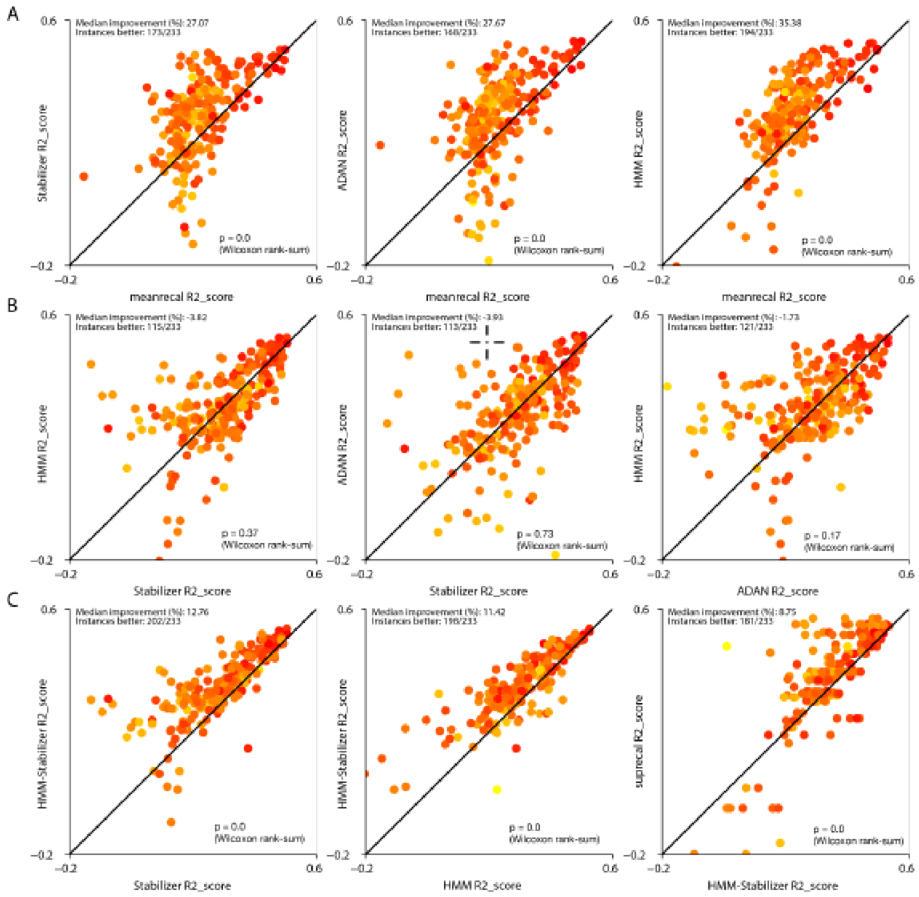

Supplementary Fig. 4 | Pairwise offline results (R2). Same as Supplementary Fig. 3 but with R2 score**. a,** Pairwise comparisons plotted for PRI-T, FA stabilizer, ADAN against mean recalibration. PRI-T, stabilizer, and ADAN all generally outperform mean recalibration (> 72% of cases for all three, $p < 0.001$ Wilcoxon rank-sum test). **b,** Comparisons among PRI-T, ADAN, and FA stabilizer. Methods are all roughly equivalent. **c,** Combined approach outperforms versus PRI-T and stabilizer individually (>84% of the time in both cases). Combined decoder is worse than supervised recalibration (supervised outperforms in ~77% of cases with a median 9% improvement).

3.On line 299, the authors state that "our offline, in-silico, and online results demonstrate an unsupervised recalibration system [PRI-T] that outperforms prior state-of-the-art [...]." Authors could be clearer with this claim, for example to specifically say the name of the method (FA stabilizer) and avoid vague statements about state-of-the-art method. Any change should keep line 27 in mind as it uses a similar sentence.

We have modified the language on lines 27 and 299:

Leveraging an offline dataset spanning five years of iBCI recordings, we further show how recently proposed data distribution-matching approaches to recalibration fail over long time scales. Only target-inference methods appear capable of enabling long-term unsupervised recalibration, while distribution-matching methods appear to accumulate compounding error over time.

Our offline, *in-silico*, and online results demonstrate an unsupervised recalibration system that outperforms RTI and subspace stabilization and helps tackle one of the remaining technical roadblocks for clinical translation of cursor BCI systems.

4.On line 104, the authors say “PRI-T appears similar to existing approaches in pairwise recalibrations but outperforms in iterative, longer-term settings.” As PRI-T is not compared to RTI in real data, this statement should be clarified. The authors are referring to distribution alignment methods?

We are referring to distribution alignment methods. We have updated the sentence below to correct for the ambiguity:

Hence, PRI-T appears similar to existing distribution alignment approaches in pairwise recalibrations but outperforms in iterative, longer-term settings. 5.It would be helpful to see the heatmaps that are shown in Figure 3 but for the supervised method to give some intuition on the expected upper-bound of performance.

Thank you for this suggestion. To see how the performance of the unsupervised methods compares to supervised training, we normalized the heatmaps by the within-day supervised performance (see below; colorbar is [0, 1]). Note that one day had a large electrical artifact in the recordings that affected the supervised performance baseline (hence the yellow streaking, where unsupervised methods outperform the supervised baseline). The plot shows very similar patterns as before, as such we decided to keep the original heatmaps in Figure 3. Note that Figure 3b shows the overall performance of each method relative to supervised performance, giving an idea of how much worse unsupervised calibration is relative to supervised calibration.

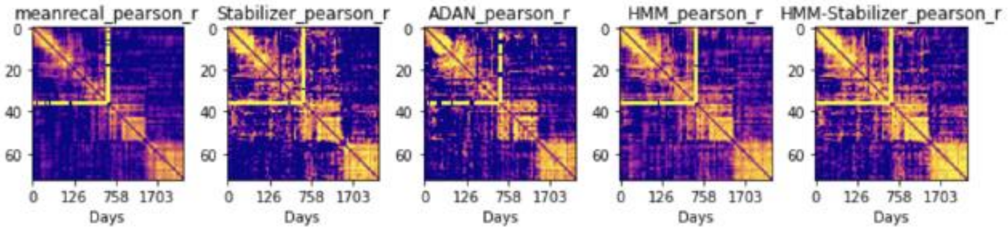

6.While this seems to be implicit, the authors should explicitly clarify how they recalibrate the decoder after the inferred target is obtained from the HMM, write the equation for the linear decoder, and how the weighted least squares is done for recalibration.

We have added the following section to the PRI-T portion of the Methods.

Decoder update. By running PRI-T on past cursor behavior, we obtain cursor-to-target intention estimates

$\hat{y}_t = \hat{H}_t - p_t$ and confidence estimates. $w_t = \max_h P(H_t = h|O_1, \ldots, O_n)^2$. Associated with these timeseries are corresponding neural data $x_t \in \mathbb{R}^{193}$, containing the firing rate of each channel at timestep *t* and an extra constant term in the first position (for the intercept). We then perform weighted least squares, where we find linear regression weights $\hat{D} \in \mathbb{R}^{2\times193}$ that minimize the (weighted) MSE loss:

$$\hat{D} = \min_D \sum_t (w_t(\hat{y}_t - D^T x_t))^2$$

This expression can be evaluated in closed-form or with standard, out-of-the-box numerical solvers (we use scipy’s linalg.lstq routine, which calls a LAPACK driver [#]).

#: Anderson, E. et al., 1999. *LAPACK Users' Guide* Third., Philadelphia, PA: Society for Industrial and Applied Mathematics.

Typos 1.There’s a handful of sentences with incorrect capitalization that should be fixed. (e.g. but not limited to line 308) 2.In line 274, Supplementary Figure 4A is probably instead referring to Supplementary Figure 7.

Thank you for catching these mistakes and our apologies for not spotting them earlier. We have corrected them in the manuscript.

Dear Mr Wilson,

Thank you again for submitting to Nature Biomedical Engineering your manuscript, "Long-term unsupervised recalibration of cursor BCIs". The manuscript has been seen by 2 experts, whose reports you will find at the end of this message. You will see that the reviewers appreciate the work, and that they raise a number of technical criticisms that we hope you will be able to address. In particular, we would expect that a revised version of the manuscript provides:

*Clarification of the specific improvements conferred by PRI-T.

*Clarification of aspects of the methodology and the conclusions with regard to the data in Supplementary Figure 5, Figure 3 and Figure 4.

*Clarification on the use of specific statistical tests and an additional information regarding multiple comparisons.

*A discussion of the limitations of PRI-T vs RTI.

*Clarification on the hyperparameter selection methodology and application of a independent validation set.

When you are ready to resubmit your manuscript, please upload the revised files, a point-by-point rebuttal to the comments from all reviewers, the reporting summary, and a cover letter that explains the main improvements included in the revision and responds to any points highlighted in this decision.

Please follow the following recommendations:

* Clearly highlight any amendments to the text and figures to help the reviewers and editors find and understand the changes (yet keep in mind that excessive marking can hinder readability).

* If you and your co-authors disagree with a criticism, provide the arguments to the reviewer (optionally, indicate the relevant points in the cover letter).

* If a criticism or suggestion is not addressed, please indicate so in the rebuttal to the reviewer comments and explain the reason(s).

* Consider including responses to any criticisms raised by more than one reviewer at the beginning of the rebuttal, in a section addressed to all reviewers.

* The rebuttal should include the reviewer comments in point-by-point format (please note that we provide all reviewers will the reports as they appear at the end of this message).

* Provide the rebuttal to the reviewer comments and the cover letter as separate files.

We hope that you will be able to resubmit the manuscript within 4 weeks from the receipt of this message. If this is the case, you will be protected against potential scooping. Otherwise, we will be happy to consider a revised manuscript as long as the significance of the work is not compromised by work published elsewhere or accepted for publication at Nature Biomedical Engineering.

We hope that you will find the referee reports helpful when revising the work, which we look forward to receive. Please do not hesitate to contact me should you have any questions.

Best wishes,

Michelle

__
Dr Michelle Korda
Senior Editor, Nature Biomedical Engineering

Reply to Reviewers

Note: reviewers' comments appear in **black text**. Our replies appear in **blue text**, and revised manuscript text appears indented (with old text shown in **black** and new edits in **red**).

We thank the reviewers for their feedback and further comments on the manuscript, which we believe have improved the quality of the work significantly. As Reviewer #1 (R1) is now fully satisfied, we focus below on the main points brought up by Reviewer #2 (R2).

Many of the concerns raised were centered around specific claims (e.g. what is considered "state of the art") and statistical testing. We have made efforts to address each of R2's points, which we believe has refined our manuscript in clarity while also improving the interpretation of results.

R1 - fully satisfied

Thank you for your time and effort. We are glad to have satisfied your concerns with the manuscript and believe that your feedback significantly improved the quality of the work.

R2:

I thank the authors for performing several new analyses that address important comments. The authors have added a simulation, showing that self-training methods are superior to distribution/subspace alignment methods (Figure 4), with the latter accumulating error over time leading to failure. They have also added 3 sessions of data from another participant to show that self-training works when using personal use data for training, which is also important. I believe the manuscript has in fact improved with these revisions. Regarding the new analyses and results, I have the following comments for the authors to address for better precision and clarity.

Thank you. We agree that the quality of the manuscript has improved with reviewer feedback and hope to further improve it based upon your comments below.

1.One main comment is regarding claims added about PRI-T outperforming "state of the art", which the authors have now added to the abstract: "Finally, we show offline that our approach also outperforms the state of the art on freeform datasets of a person using a home computer with an iBCI." I presume "state of the art" implies RTI here, as that is what the authors compare with in the 3 sessions from participant T11. However, I believe this claim is too broad and not sufficiently precise, and thus should either be removed from the abstract (and elsewhere) or made much more specific. Here are the reasons. First, PRI-T and RTI perform similarly in simulations as the authors state on line 277 "Both approaches had similar performance". Only for short training data of less than 2 minutes PRI-T does better in simulations (Fig 4c,e). As I mentioned before, I don't think this data efficiency is that critical because the data is free-use data and thus abundant -- we expect a user to use their BCI freely for more than 2 minutes per day for their daily tasks, indeed we expect hours of free-use.

Thank you for this point. We initially meant “state of the art” to refer to both RTI and FA stabilization. However, we agree that this phrase lacks precision, both in the context of the manuscript (mixing our comparison results together) and the larger field (alternatives have been tested offline and online, in humans and monkeys, with no clear definition or agreement yet on what should be considered proper SOTA). We have consequently modified the language in the Abstract:

> Our approach outperforms ~~the state of the art~~ existing strategies in large-scale, closed-loop simulations over two months, and in closed-loop with a human iBCI user over one month. Leveraging an offline dataset spanning five years of iBCI recordings, we further show how recently proposed data distribution-matching approaches to recalibration fail over long time scales. Only target-inference methods appear capable of enabling long-term unsupervised recalibration, while distribution-matching methods appear to accumulate compounding error over time. Finally, we show offline that our approach also performs well ~~outperforms the state of the art~~ on freeform datasets of a person using a home computer with an iBCI.

We agree that, in a high-channel count regime with sufficient training data, data efficiency concerns are likely minimal; in this setting, auto-calibration strategies can simply run through the entire day to account for accruing neural changes. During the start of the day, however, users must contend with a larger set of changes due to overnight shifts. In this scenario, one could

imagine the frustration of having to recalibrate e.g. your smartphone every morning before checking texts or emails. An added benefit of course is that we no longer need active selections, making the approach more suited for personal use settings.

Second, the data from T11 is from 3 sessions, and in 2 of 3 sessions there is no significant difference with sufficient data (session 665 and 672) and in 1 of the 3 sessions there is no difference even for short data (Fig. 6D, left panel, session 665). Similarly, the authors should be more specific in the discussion line 378 what they mean by PRI-T outperforming RTI. If the authors mean improved data efficiency, they should specifically say that and clarify for how much data and how often they see this improvement. A discussion of when such data efficiency is important would also be helpful. I think what is convincingly shown in this manuscript is the general advantage of self-training methods (PRI-T, RTI) over distribution alignment ones, which is an important finding for the field.

We agree that the main impact of this work is its implications for self-training versus distribution alignment based approaches. We have consequently modified the Discussion to center this issue more clearly:

> Our offline, *in-silico*, and online results demonstrate an unsupervised recalibration system that outperforms ~~RTI and~~ subspace stabilization and helps tackle one of the remaining technical roadblocks for clinical translation of cursor BCI systems. By providing consistent control without supervision - despite compounding neural nonstationarities - these findings demonstrate a way for BCI users to maintain robust control without frequent pauses for supervised recalibration. Our work also demonstrates that iterative or "chaining" based strategies may be necessary to compensate for long-term signal drift and that, at least in simulation, only target-inference methods such as PRI-T and RTI appear capable of indefinite chaining.

2.Related to the above, there are other instances of "state of the art" in the abstract and elsewhere. For example, in the abstract, it is stated "Our approach outperforms the state of the art in large-scale, closed-loop simulations over two months, and in closed-loop with a human iBCI user over one month", where "state of the art" this time likely refers to FA stabilization and not RTI (since RTI was not compared with online). As such the usage of "state of the art" seems inconsistent in the manuscript/abstract. In general, I don't believe there exists an agreed upon unsupervised "state of the art" for recalibration, because BCIs have largely used supervised recalibration thus far and most unsupervised methods are simply tested offline. For precision, I believe the authors should remove such notions of "state of the art" and instead be specific about which methods they are referring to (distribution alignment or self-supervision/RTI) and what the specific improvement is and in what regime it is observed. This will make the conclusions clearer and the manuscript more precise.

Apologies for the unclear language – we hope our response to point (1) addresses this concern.

3.The authors use 3 separate sessions with participant “T11”. Is there a reason more sessions were not used to provide more general conclusions (see also comment 1 above)? How were these 3 sessions chosen? Also, why not compare with subspace alignment (FA stabilization) on this data offline?

These data were obtained from collaborators based on a few requirements. First, we needed sessions that contained both personal use and target acquisition task data on the same day (which removed a large number of days). Second, we required a sufficiently high SNR from these data such that reasonable control was achieved throughout the session; T11's control is more intermittent compared to T5, and decent control was not achieved consistently on all days. The three selected sessions contained the longest recordings with both target acquisition data and personal use data on the same day, with the highest SNR, and thus were the most relevant sessions for testing PRI-T and RTI. We have modified the Methods to include this note:

> During these sessions, T11 was using his iBCI system for personal use, performing various self-prompted tasks such as email typing, video calls, and web searches (Fig. 6A,B). T11 used a set of discrete gestures to perform multiple selections. We selected these specific sessions based on several criteria: 1) sufficiently high SNR such that T11's control was reasonable throughout the session, 2) inclusion of both personal use and target acquisition task blocks, and 3) long recording times.

An exciting direction would be to fully characterize personal use much as we did here with T5's recordings. However, a full study of unsupervised retraining in this personal use setting is in our opinion outside of the scope of work presented here but nevertheless an exciting (and indeed, crucial) next step.

We did not compare against FA stabilization as that technique requires a pair of days - the first is used to fit the original decoder, and the second day's activity is aligned to the first. Here, we are only examining days individually, in order to test whether target inference methods can successfully infer the target in unstructured tasks. Comparing the performance of FA stabilization in unstructured tasks is an interesting direction for future work, but 1) would require more close-in-time session days and 2) in our opinion is outside the scope of this work.

4.In new Figure 4, what is the PD norm (SNR) value that best matches the human data? I assume it corresponds to panels a-c which are the primary simulations? Please clarify. In Figure 4, the advantage over RTI is at one of the SNR levels (PD norm 0.24), is this a realistic SNR based on human data?

We simulate a distribution of PD norm (SNR) values that is designed to closely approximate T5's (see Supplementary Fig. 6). In panels a-c, we sample from this distribution on each new day.

In Figure 4, T5's median SNR corresponds to a PD norm of 0.58 (high end). T5's worst days would map to around 0.3, and hence the unstable regime around 0.24 is unlikely for his

recordings. Note that T5 has generally higher performance than other clinical trial participants, so this setting is likely more relevant for participants with worse control or alternative modalities (e.g. ECoG, EEG).

We have added a clarification to the Discussion to contextualize this result:

> We also considered how robust each model was to the simulated neural population's SNR (Fig. 4C). We fixed the population tuning matrix norm at different strengths (see Methods) while running the simulator for 30 days, then measuring performance on the last day as before. Interestingly, at a critical point RTI worsens significantly compared to subspace stabilization and PRI-T (PD-norm of 0.24). This reveals a possible failure mode of RTI: As SNR drops, the number of dwell-based clicks drops due to a lack of successful trials. At this point, subspace stabilization becomes preferable to RTI ($p < 0.001$ up until the smallest SNR setting). However, since PRI-T uses velocity information it is much more robust to this issue and maintains a lead over subspace stabilization ($p < 0.001$ all). Note that T5's median SNR corresponds to a PD norm here of approximately 0.58; hence these robustness benefits are likely more relevant for participants with worse control or in less performant signal modalities.

5.I am having difficulty understanding the statistical comparisons in the new Figure 6 and new Supplementary Fig 8. The authors mention "Encouragingly, PRI-T had similar accuracy to the oracle when provided sufficient data (Fig. 6D, right panels)." First, the green and cyan lines seem to be different, how is lack of significance assessed? What is N in these tests and what does each data point (N) represent?

Our apologies for this lack of clarity. The statistical unit here was the correlation during a 30-second segment, and significance was assessed by using the Wilcoxon signed-rank test over the independent 30-second segments. We have modified the supplementary description as follows to clarify this point:

> As in the offline T5 analysis, we use decoder output correlations with the point-at-target vector as a performance metric. We split test blocks into 30 second segments to obtain multiple independent measures of performance for each recalibration method (enabling variance quantification and significance testing), yielding N = 38, 18, 19 distinct segments for days 665, 672, and 702 respectively. We used a Wilcoxon signed-rank test to evaluate significant differences between the approaches. We removed the first 60 seconds of each test block from evaluation to reduce the impact of mean shifts and initial participant adjustment to the task.

Second, how does RTI compare with oracle? Is that in Supplementary Fig. 8?

RTI generally underperformed compared to the oracle. See Supplementary Figure 8 panels B,D,F (bottom right comparisons).

Third, the authors seem to be performing repeated comparisons for each point on the x-axis, which thus requires a correction for multiple comparisons. It does not seem like this correction is applied, is it? Please apply a correction such as Benjamini-Hochberg or False discovery rate control.

We have updated Figure 6 to include FDR-corrected p bevalues along the x-axis (Benjamini-Yekutieli, as tests are not independent). During this process, we found a bug in the analysis code that yielded incorrect p-values. After correcting the mistake, the main takeaways are largely the same (PRI-T is generally more data-efficient than RTI at smaller dataset sizes, and click PRI-T does not have a convincing improvement over PRI-T). However, PRI-T does appear to have a statistically significant lower performance compared to the oracle baseline. We have updated the Results accordingly:

> Encouragingly, PRI-T's performance gap with the oracle tended to lessen – but not disappear – when provided increasing amounts of data (Fig. 6D, right panels)

The relevant panel is reproduced below:

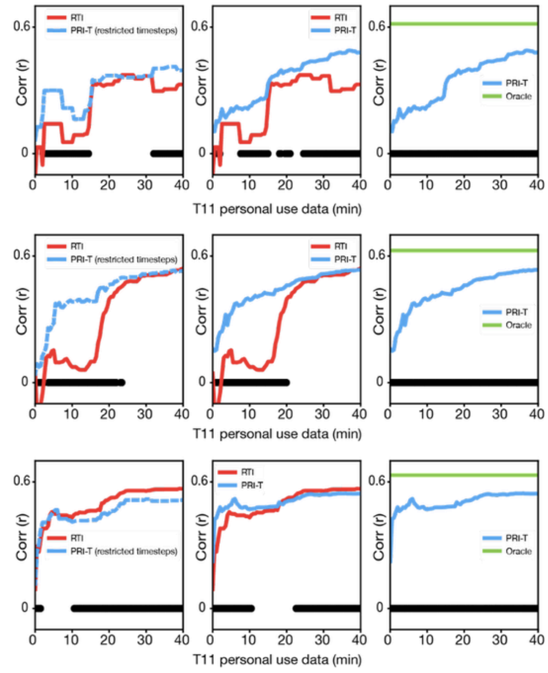

We have modified the Methods to include this step:

> Statistical significance at each dataset size was evaluated using a Wilcoxon signed-rank test (false discovery rate controlled via Benjamini-Yekutieli).

Finally, when statistical comparisons are pair-wise, why do the authors use the Wilcoxon rank-sum test rather than the signed rank test?

Thank you for this comment and our apologies. We mislabeled the tests in Supplementary Figures 3 and 4 as Wilcoxon rank-sum when they were in fact done with signed-rank. The images have been updated alongside their captions (e.g. S3's below):

> **Supplementary Fig. 3 | Pairwise offline results (Pearson correlation). a,** Pairwise comparisons plotted for PRI-T, FA stabilizer, ADAN against mean recalibration. PRI-T, stabilizer, and ADAN all generally outperform mean recalibration (> 75% of cases for all three, $p < 0.001$ all Wilcoxon signed-rank test)...

6.I thank the authors for providing the RTI with longer look back, which is interesting. One question I had in my previous comments was whether PRI-T is better than RTI due to more accurate estimates of targets and/or the use of the confidence estimates. As I mentioned previously, this could be explicitly tested for by also comparing to PRI-T with equal confidence weights as was done in previous Supp Fig 5a/b but now with sweeps of data size. I am not sure what the conclusion was regarding that?

We have added an analysis of T11's personal use data in supplementary figure 8 where we compare PRI-T with and without timestep weighting (reproduced below). Unlike the offline, structured task results with T5 (Supplementary Fig. 5), which suggested that confidence weighting improved PRI-T's performance relative to no weighting (discrete prob threshold = None), the T11 data show a strong improvement without the use of weighting:

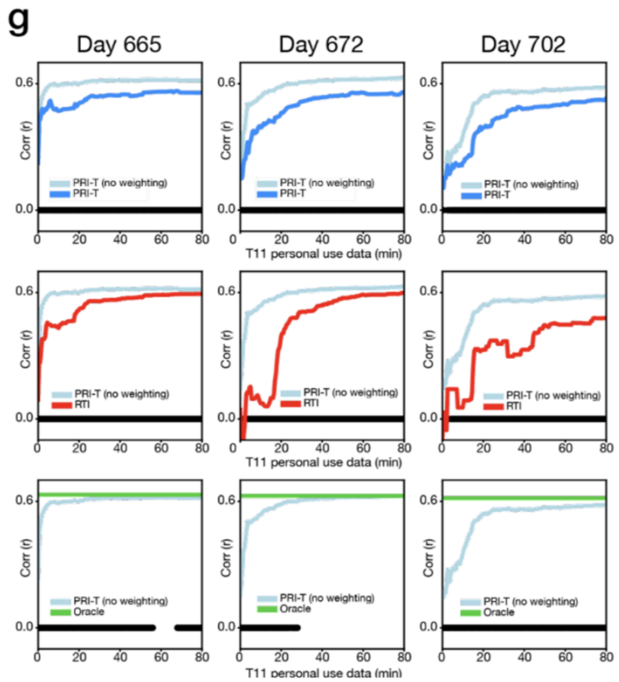

This may be due to a number of factors (for instance, differences between structured and unstructured task settings; differences in cursor behavior between T5 and T11) although it is

difficult to explore fully with just a few personal use sessions. We have modified the Results section accordingly:

> Encouragingly, PRI-T's performance gap with the oracle tended to lessen – but not disappear – when provided increasing amounts of data (Fig. 6D, right panels) or when using a variant without any timestep weighting (Supplementary Fig. 8G), which also generally outperformed RTI across a range of dataset sizes. These results imply that, with sufficient retraining and parameter tuning, PRI-T could be able to achieve a degree of iBCI cursor control competitive with supervised retraining in an everyday setting.

Fully disentangling the contribution of timestep weighting on personal use data will require a more comprehensive dataset than the preliminary one here. We believe that adapting PRI-T for robust control across multiple clinical trial participants is an interesting and important direction for future work.

7.The authors discuss the advantages of PRI-T vs. RTI, but what are the limitations of PRI-T vs RTI? Is fitting the probabilistic model prone to errors for example if a lot of grid points for target location are needed? Or if the task environment is more general or tasks are more complex? To me one advantage of PRI-T which may be more important than data efficiency is the fact that the user does not need to be actively making selections. Please discuss pros and cons of both methods.

We have added a section on benefits and drawbacks to the Discussion:

> By extending RTI's self-training approach to incorporate cursor information, PRI-T enables unsupervised recalibration during periods with few active selections, such as during drop down menu usage and hovering, while also increasing data efficiency. This latter benefit may be especially useful after periods of inactivity (e.g. at the start of the day), when neural changes have accrued enough to impair high-performance control. This benefit comes at the cost of increased complexity however, with additional hyperparameters compared to RTI and an increased compute overhead to perform a Viterbi search.

8.The authors perform a new nice simulation in which they let errors accumulate with the subspace stabilization and then apply different recalibration methods, showing that the self-training methods can remove the error but subspace stabilization cannot (Fig. 4e). But in Figure 3C, they conclude that "Results show that PRI-T, ADAN, and FA stabilization all perform poorly in the face of large rotations of the subspace ..., and seem to fail completely for rotations larger than roughly 90 degrees (albeit with differing variability; Fig. 3C)." Are these two conclusions in Fig. 3 vs Fig. 4 consistent? If large rotations lead to failure, how could RTI and PRI-T both correct for it in Figure 4 after error accumulation and thus presumably large rotations? Please discuss how these are consistent results.

Thank you for raising this point. Offline, it appears that all methods have some alignment error that's proportional to the encoding angle change (Figure 3C). Online, target inference methods

appear to be able to drive the error to zero given enough data, even in the face of a large encoding angle change (Figure 4E).

We believe that the reason lies in a crucial difference between the offline and closed-loop settings. When a user is driving a misaligned decoder in closed-loop, the user will implicitly correct for any decoding errors that happen using visual feedback control. With these corrections, the decoder outputs become more informative of the user's intended target locations (after all, the cursor is eventually driven to the correct target given enough time, as long as control is above chance). But in the offline testbed setting, the user was originally driving a good decoder, but target inference methods are only given access to the misaligned decoder outputs which do not contain user corrections. We have added the following paragraph to the results section to clarify this point:

> The above results also raise an apparent contradiction - how is it that RTI and PRI-T have poor performance in the offline setting when large errors in the decoder have accumulated (Fig. 3C), but are able to drive errors to zero in the closed-loop setting even in the face of initially large decoder errors (Fig. 4E)? We believe the answer lies in a crucial difference present in the closed-loop setting. In closed-loop, the user is driving the cursor towards the target with the poorly-aligned decoder, while using visual feedback to correct for any decoding errors which occur due to misalignment. These user corrections make the decoder output more informative of the true target location, allowing target inference methods to more accurately estimate the user's target. As long as control quality is reasonable enough, the user will eventually reach the intended target, which can then be inferred from the decoder output. However, in the offline testbed setting, these corrections cannot be applied (given that target inference methods are only given access to the misaligned decoder outputs), leading to worse target inferences.

9.In terms of hyperparameter selection, it seems the author do so based on performance on test data? For example, the authors state on line 764 that "We select the combination that yields the highest performance on the holdout task blocks (see T11 section)". If so, doing the selection based on test data is prone to overfitting to test data. Instead hyperparameters should be selected based on a validation set that is not used in either training or test, please clarify.

Thank you for pointing out this lack of clarity in the methods. In our view, since we're only optimizing Click PRI-T and RTI's parameters in this way on the T11 data (and *not* optimizing PRI-T parameters), any overfitting should only make click PRI-T/RTI better relative to PRI-T. But still, we find that RTI lags behind PRI-T in data efficiency (and that click PRI-T is largely identical). Overfitting to the test set was indeed our intention here, in order to give these approaches the best chance possible to outperform PRI-T, and was simpler and more straightforward than defining an additional validation set (which may not be large given the limited amount of data we had access to here). That being said, In practice this will likely not significantly influence the results as RTI only has 3 parameters.

We have modified the Methods section to clarify this point:

We select the combination that yields the highest performance on the holdout task blocks (see T11 section). Here we explicitly give click PRI-T the best possible chance of outperforming the standard PRI-T integration, but still find that the two are largely the same.

…

We used T5's PRI-T hyperparameters here for T11. For click PRI-T and RTI, we swept hyperparameters while measuring mean performance across 30-second folds on the test data. Sweep settings and results are replicated below (see Supplementary Fig. 8 as well). Selecting RTI and click PRI-T parameters based on held-out task performance gives them the best chance against PRI-T; nevertheless, we still found that PRI-T outperformed RTI in terms of data efficiency while rivaling click PRI-T.

10. I still find the pattern in Figure 3 a bit confusing even now that days apart are shown. For example, the pattern after around day 500 (lower right yellow square) is different from that between day 1-200 (upper left square). What does this imply or mean? It seems to mean that correlations across days suddenly increases after day 500? If so is there a hypothesized reason for that?

Those sessions after day 500 are much closer together, as we were collecting closed-loop sessions frequently during that time; thus, they were more correlated. The upper left box is from T5's early trial days, when we were also collecting data frequently (just not as much).

Reviewer #2 (Report for the authors (Required)):

I thank the authors for addressing my comments. I think the paper has improved and I recommend publication after the below points are addressed:

1) One technical distinction of PRI-T vs. RTI is the confidence weighting as the authors state on line 180. But the new analyses (supp fig 8g) show that this weighting does not necessarily help and can actually hurt performance on the personal use data. This implies that it can be better to use PRI-T without this confidence weighting in some tasks. In particular, the authors state in their response: "Unlike the offline, structured task results with T5 (Supplementary Fig. 5), which suggested that confidence weighting improved PRI-T's performance relative to no weighting ..., the T11 data show a strong improvement without the use of weighting". Currently the methods Line 817 still states that confidence weighting helps and refers to Supplementary Fig. 5A. These statements need to be corrected/amended in light of the new results so that readers know that confidence weighting in some cases can be hurtful and so that they can test for themselves whether they need it or are better off not using it. Also, the current discussion section does not discuss this topic. It should be made clear in discussions and method that confidence weighting is not necessarily helpful and users should compare their PRI-T approach with and without it, with a reference to the new Supplementary figure 8g.

Thank you for this comment, we have now fully updated the manuscript with these new findings. We have modified the Methods section:

> During hyperparameter sweeps, we found that using weighted least squares for incorporating timepoints based on how confident the model is about the target location was superior to using all timepoints equally or applying a discrete cutoff in T5's offline data (Supplementary Fig. 5A). Note however that confidence weighting was strictly worse in T11's personal use data (Supplementary Fig. 8G), implying that this setting may be participant or task-dependent and should be evaluated in future PRI-T implementations.

We have also modified the Discussion to highlight this point:

> This latter benefit may be especially useful after periods of inactivity (e.g. at the start of the day), when neural changes have accrued enough to impair high-performance control. This benefit comes at the cost of increased complexity however, with additional hyperparameters (including whether to use confidence weights, see Methods and Supplementary Fig. 8G) compared to RTI and an increased compute overhead to perform a Viterbi search.

2) In the abstract, it appears by "existing strategies" authors are referring to their closed-loop results in which they compare with distribution-matching methods. Please revise to clarify this.

We have modified the Abstract accordingly:

> Our approach outperforms ~~existing strategies~~ distribution alignment methods in large-scale, closed-loop simulations over two months…

3) Please clarify superior performance is over FA in this section title: “PRI-T demonstrates superior performance during one month of closed-loop control with T5.”

We have modified the section title accordingly:

> **PRI-T demonstrates superior performance to FA stabilization during one month of closed-loop control with T5**

4) On line 456 where pros and cons versus RTI are discussed, please put a discussion of how the number of grid points for target location can be determined for PRI-T and how the increased number of grid points depending on task complexity could potentially affect performance or complexity of method. These are important points for users to know.

We have added an additional note to the paragraph recommending that users begin with our settings and then incrementing upwards:

> To optimize the number of target grid points, we recommend beginning with a 20 x 20 arrangement (as in this work with a standard display) and then scaling based on held-out performance and compute constraints.
